# Conformational Diversity-Driven Crystallization of Daptomycin: A Multi-Scale Approach with Experimental Validation

**DOI:** 10.3390/pharmaceutics18060657

**Published:** 2026-05-27

**Authors:** Qingshi Wen, Ke Zhang, Li Huang, Shuyang Zhou, Hanjie Ying, Pengpeng Yang

**Affiliations:** College of Biotechnology and Pharmaceutical Engineering, National Engineering Technique Research Center for Biotechnology, Nanjing Tech University, No. 30, Puzhu South Road, Nanjing 211816, China; wenqingshi2024@163.com (Q.W.); yans163z@163.com (K.Z.); 19850062217@163.com (L.H.); zhouyang05162021@163.com (S.Z.);

**Keywords:** daptomycin, conformational diversity, COSMO-RS, molecular dynamics, solubility

## Abstract

**Background:** Daptomycin, a lipopeptide antibiotic with critical clinical applications, presents a formidable crystallization challenge due to its high conformational flexibility and complex ionization equilibrium. Current literature lacks reports on single crystals or highly crystalline powders of this molecule. This study aims to elucidate the thermodynamic and kinetic mechanisms governing daptomycin solubility and crystallization to establish a rational screening pathway. **Methods:** In this study, the solubility of daptomycin was systematically measured across eight pure solvents using a static gravimetric method. Molecular-level insights were obtained by integrating experimental data with the Conductor-like Screening Model for Real Solvents (COSMO-RS) and molecular dynamics (MD) simulations. **Results:** Solubility trends correlated strongly with solvent electrostatic and hydrogen-bonding capabilities. MD simulations revealed that the solvent’s ability to modulate conformational diversity—quantified by the number of dominant conformational clusters—is the decisive factor governing crystallization. For instance, aqueous systems exhibited strong Coulombic stabilization (−1126.61 kJ/mol). Crucially, solvents like acetone restricted daptomycin to a limited number of conformers (12 clusters), significantly lowering the nucleation barrier and yielding crystalline powders with distinct PXRD peaks. Conversely, solvents like methanol induced high conformational diversity (53 clusters), resulting exclusively in amorphous precipitates. **Conclusions:** The “number of conformational clusters” serves as a robust descriptor for rapidly screening crystallization solvents, effectively bridging thermodynamics and kinetics. This strategy provides a rational, reduced-trial-and-error framework for crystallizing complex, flexible macromolecules with multiple dissociation sites, moving beyond traditional trial-and-error approaches.

## 1. Introduction

Crystallization, a critical step in controlling the solid-state form, purity, stability, and bioavailability of drugs, remains a central focus in pharmaceutical development. While crystallization processes for small-molecule drugs are relatively well-established, the crystallization of biomacromolecules (proteins, peptides) and structurally complex synthetic drugs still presents significant challenges [1]. These challenges primarily stem from the inherent high conformational flexibility, complex surface charge distribution, and diverse intermolecular interactions characteristic of such molecules, which collectively contribute to elevated nucleation energy barriers, narrow crystallization condition windows, and low predictability [2,3,4].

The crystallization of biomacromolecules is governed by a complex interplay of thermodynamic and kinetic factors. From a thermodynamic perspective, the high conformational entropy at the molecular surface presents a major barrier to the formation of an ordered crystal lattice. Studies have shown that surface-exposed flexible residues (lysine, glutamic acid) act as an “entropic shield” due to their high conformational entropy, thereby inhibiting the formation of crystal contact interfaces [5]. To address this, the Surface Entropy Reduction (SER) strategy was developed. This approach involves the site-directed mutagenesis of high-entropy residues to smaller side-chain residues like alanine, thereby reducing local conformational entropy. It has been successfully applied to the rational design and crystallization optimization of various recalcitrant proteins [6,7,8].

At the methodological level, classical physicochemical techniques driven by solution supersaturation remain fundamental to crystallization screening. Vapor diffusion methods (hanging-drop, sitting-drop) and liquid diffusion methods (free-interface diffusion, gel diffusion, dialysis) gently induce supersaturation by slowly altering the chemical potential, and they remain the mainstream techniques for obtaining protein and peptide single crystals. In contrast, methods like anti-solvent crystallization and cooling crystallization induce nucleation by rapidly changing solvent composition or system temperature, and are commonly used for preparing microcrystals or amorphous solid dispersions of peptides or poorly soluble drugs. To significantly enhance screening efficiency, automated high-throughput screening (HTS) has become the standard workflow. Utilizing automated liquid-handling workstations, thousands of conditions—comprising different precipitants (polyethylene glycol, salts), buffer systems, pH values, and additives—can be tested in parallel in 96-, 384-, or even 1536-well plates. This enables the systematic and rapid exploration of the vast “crystallization condition space” [9,10]. Furthermore, to address specific challenges, a range of auxiliary crystallization strategies have been developed and widely implemented. For instance, metal-ion-assisted crystallization (utilizing ions like calcium, magnesium, zinc) stabilizes specific molecular conformations or mediates intermolecular crosslinking via coordination bonds [11]. Additive engineering (using polyols, amino acids, cyclodextrins, detergents) allows for fine-tuning of the solution’s physicochemical environment and intermolecular interactions [12]. External field-assisted crystallization (using ultrasound, electric fields) can influence nucleation kinetics and induce molecular alignment [13]. Meanwhile, co-crystallization techniques provide an innovative pathway for structural determination of challenging molecules (liquids or oils) by employing “crystallization chaperones” to form host–guest or supramolecular complexes with the target molecule [14]. The common core of these strategies lies in addressing the high conformational diversity and poor packing propensity of target molecules. By introducing external media or physical fields, they facilitate the transition of the solute from a high-entropy, disordered solution state to a low-entropy, ordered crystalline state, thereby lowering the nucleation barrier and enhancing crystallization success rates.

In terms of specialized crystallization strategies, a variety of techniques have been developed to provide targeted solutions for challenging systems. Supramolecular gel crystallization utilizes the three-dimensional networks formed by the self-assembly of low-molecular-weight gelators as intelligent media. The spatial confinement effects of these gels can modulate crystallization kinetics, improve crystal quality, and influence polymorph selection, thereby opening new avenues for the crystallization of hydrophobic drugs [15]. For membrane proteins, the lipidic cubic phase (LCP) crystallization method has emerged as a key technology for structural determination. This technique embeds proteins within a lipid bilayer structure that mimics the cell membrane, providing the essential hydrophobic environment [16]. Mesoporous material-templated crystallization employs nanochannels (silica with pore sizes of 2–50 nm) as confined spaces, promoting the ordered arrangement of flexible molecules within these restricted environments [17]. Furthermore, covalent or non-covalent immobilized crystallization involves tethering target molecules to solid supports (resins, magnetic beads). This approach reduces disordered collisions in solution, which can favor the formation of crystal nuclei [18].

To understand and prospectively control crystallization processes, it is essential to establish a multiscale research framework spanning from the macroscopic to the microscopic level. At the macroscopic scale, precise solubility measurements are indispensable for constructing solid–liquid phase diagrams and optimizing crystallization processes. At the mesoscopic and microscopic scales, computational tools are playing an increasingly pivotal role. The Conductor-like Screening Model for Real Solvents (COSMO-RS), based on quantum chemical calculations, can efficiently predict key thermodynamic properties of molecules in different solvents, such as solubility and activity coefficients. It has been successfully applied in areas like rational solvent screening and co-crystal design [19,20,21]. Molecular Dynamics (MD) simulations can, at atomic resolution, reveal the conformational ensemble of solute molecules in solution, their solvation structures, the dynamics of hydrogen-bond networks, and the evolution of pre-nucleation clusters. This provides a unique perspective for understanding molecular flexibility, solvent effects, and conformational diversity, and can be employed for the preliminary assessment and screening of crystallization systems. Studies have shown that analyzing protein conformational entropy and surface properties through MD simulations can effectively predict crystallization propensity and guide rational design [22]. In terms of characterization techniques, X-ray Diffraction (XRD) is the gold standard for determining the atomic-level structure of crystals, with the width and intensity of its diffraction peaks directly reflecting the crystal’s long-range order. Small-Angle X-ray Scattering (SAXS), as a powerful solution-state characterization technique, can obtain information on the overall shape, size (radius of gyration, Rg), oligomeric state, and conformational flexibility of biomacromolecules in near-native, pre-crystallization conditions. It is particularly suitable for studying flexible or dynamic systems that are difficult to crystallize. SAXS and MD simulations, from the perspectives of experimental characterization and theoretical computation respectively, provide critical microscopic evidence and mechanistic insights for the initial screening of solvents and optimization of conditions during the early stages of crystallization [23].

**Figure 1:**
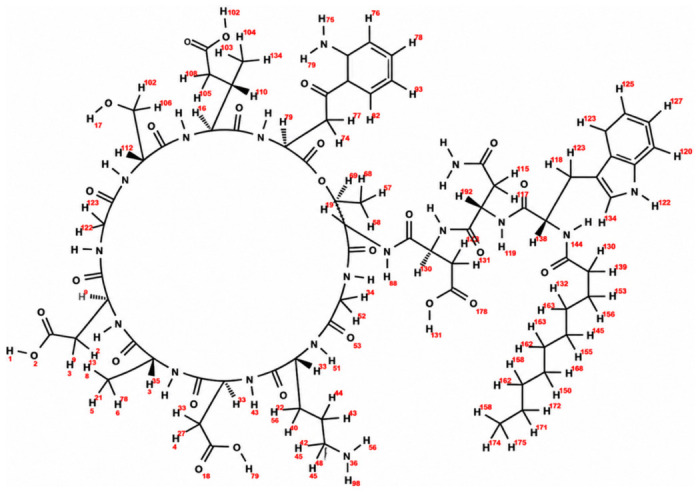
Chemical structure of daptomycin.

**Figure 2:**
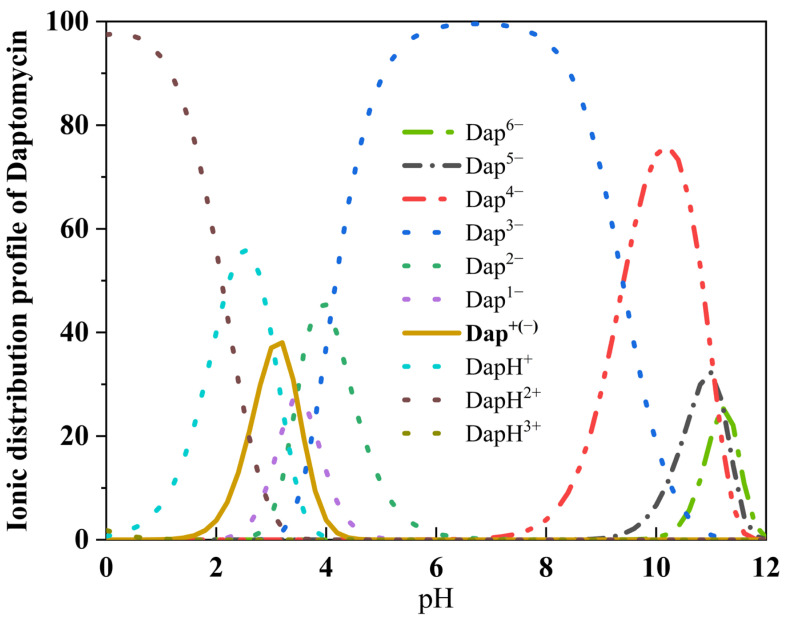
Ionic distribution profile of daptomycin vs. pH in aqueous solution (Dap = daptomycin; superscripts indicate net charge).

Daptomycin (Figure 1) exemplifies the challenges described above. As a cyclic lipopeptide antibiotic (C_72_H_101_N_17_O_26_, molecular weight: 1620.67 g/mol, CAS No. 103060-53-3) produced by Streptomyces roseosporus, its unique antibacterial mechanism makes it a vital drug against Gram-positive pathogens. However, its molecular structure is complex, comprising a 13-membered cyclic peptide, an exocyclic tail, and a decanoyl side chain, which collectively confer exceptionally high conformational flexibility. Furthermore, the presence of multiple ionizable groups on the molecular surface results in a strong pH-dependent charge state. As shown in Figure 2 (an ion distribution profile at different pH values, predicted using MarvinSketch 24.3.2 based on dissociable groups), the proportion of the neutral molecular species of daptomycin (indicated by the solid line in the figure) does not exceed 40%, even near its isoelectric point (pI ~ 3.5). This indicates that the concentration of “crystallographically active species” available in solution for ordered lattice packing is consistently low. The combination of these three factors—high flexibility, complex charge distribution, and low effective concentration of crystallizable species—makes the crystallization of daptomycin exceptionally difficult. To date, no reports of high-quality single crystals or highly crystalline powders of daptomycin have been published, significantly hindering in-depth research into its solid-state properties, polymorph control, and process optimization [24,25].

**Figure 3:**
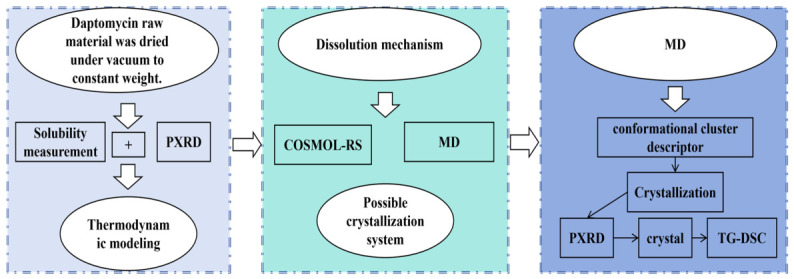
Research Workflow.

To address the crystallization challenge of daptomycin, this study aims to establish and implement a closed-loop, multiscale research strategy. We propose the central hypothesis: by rationally selecting solvent environments that simultaneously restrict daptomycin’s conformational flexibility and optimize the intermolecular interactions of its neutral form, we can significantly promote its crystallization. Through qualitative investigations of daptomycin’s conformational space, dynamic flexibility, and solvation structure in different solvents, this work seeks to reveal, at the molecular level, how solvents influence crystallization propensity by limiting conformational freedom. Our approach provides new insights into the underlying relationship between solvent, conformation, and crystallizability. The detailed research methodology is illustrated in Figure 3.

## 2. Experimental Section

### 2.1. Materials

Daptomycin (≥98% purity, initial water content ~8%) was obtained from Hubei Enzerpharm Medical Technology Co., Ltd. (Wuhan, China) and dried to a water content below 2% (*w*/*w*) (the sample was dried in a vacuum oven at 50 °C and −0.09 MPa until a constant weight was achieved, and the residual moisture content was determined to be below 2% by Karl Fischer titration) before all experiments. Ultrapure water (resistivity ≥ 18.2 MΩ·cm at 293.15 K) was generated using an EPED-30TH purification system. All other solvents and chemicals were of analytical reagent (AR) grade (Sinopharm Chemical Reagent Co., Ltd., Shanghai, China) and used without further purification. The solubility of daptomycin was measured in triplicate via the static equilibrium method across the specified temperature range. Detailed specifications of all materials are provided in Table 1 [26].

### 2.2. Solubility Measurement

The solubilities of daptomycin were experimentally evaluated in eight pure solvents: water, methanol, ethanol, 1-propanol, 2-propanol, acetone, acetonitrile, and n-hexane. To prevent solvent evaporation during the equilibrium process, especially at elevated temperatures, all samples were sealed in glass vials equipped with polytetrafluoroethylene (PTFE)-lined caps. The experimental procedure was conducted as follows: First, 3 mL of each solvent was transferred into a clean, dry 10 mL glass vial, which had been pre-equilibrated in a thermostatic bath for 20 min. The temperature was maintained within ±0.1 K of the target value using a PID control system. Under isothermal conditions, an excess amount of solid daptomycin was added incrementally to each vial until a persistently undissolved solid phase was observed. The mixture was then magnetically stirred at 400 rpm for 8 h under precise temperature control to ensure solid–liquid equilibrium. Subsequently, the solution was allowed to settle undisturbed for 4 h at the same controlled temperature (±0.1 K) to achieve complete phase separation before sampling.

After sedimentation was complete, the supernatant was sampled using a syringe maintained at the same temperature as the equilibration temperature (±0.1 K), and then filtered through a 0.22 μm organic-phase filter membrane to obtain a saturated daptomycin solution. The filtrate was then diluted to a concentration within the linear detection range of high-performance liquid chromatography (HPLC) and quantified using a validated HPLC-UV calibration curve. For the determination of mole fraction solubility (x_1_), 1.00 mL of the saturated solution was accurately transferred into a pre-weighed sample vial (initial mass, m_0_), and the total mass (m_1_) was recorded. The vial was subsequently dried under vacuum at 323.15 K until the mass change was less than 0.01 mg per hour, and the final mass (m_2_) was recorded for solubility calculation [27].

(1) $$x_{1}=\frac{(m_{2}-m_{0})/M_{c}}{(m_{2}-m_{0})/M_{c}+(m_{1}-m_{2})/M_{s}}$$

The symbols used in the solubility calculations are defined as follows: *x*_1_ represents the mole fraction of daptomycin in the saturated solution, *m*_0_ is the mass of the empty sample vial (g), *m*_1_ denotes the total mass of the vial containing the saturated solution (g), and *m*_2_ signifies the mass of the vial containing the dried solute after solvent removal (g). The molar masses of daptomycin and the solvent are denoted by M_C_ and M_S_ (g·mol^−1^), respectively.

### 2.3. Powder X-Ray Diffraction (PXRD)

To assess potential polymorphic transformations of daptomycin in different solvents, the powder X-ray diffraction (PXRD) was performed on both the raw API (active pharmaceutical ingredient) powder and the undissolved solid residues recovered from the saturated solutions in each solvent. All solid samples were vacuum-dried at 323.15 K until the mass change was less than 0.01 mg per hour to ensure complete solvent removal. The dried solids were then gently ground in an agate mortar to produce a homogeneous fine powder and uniformly packed into the sample holder to minimize preferred orientation effects. PXRD patterns were collected using a Cu Kα radiation source (λ = 1.54056 Å) operating at 40 kV in continuous scan mode. Data were acquired over a 2θ range of 5° to 45° with a step size of 0.02° and a scanning speed of 10 °/min, using a 0.2 mm receiving slit [28].

### 2.4. Thermogravimetric Analyzer and Differential Scanning Calorimetry (TG-DSC)

The thermal properties of daptomycin powder, including melting point (T_m_), moisture content, and enthalpy of fusion (ΔH_fus_), were characterized using thermogravimetric analysis (TGA) and differential scanning calorimetry (DSC). The TA Instruments Q500 (TA instruments, New Castle, DE, USA) analyzer was employed for TGA, and the TA Instruments Q2000 calorimeter was used for DSC. Samples weighing between 5.0 mg and 10.0 ± 0.1 mg were placed in aluminum crucibles and heated from 293 K to 673 K at a constant rate of 10 K/min under a dynamic nitrogen atmosphere with a flow rate of 15 mL/min. In TGA measurements, the observed mass change is primarily attributed to the loss of volatile components (such as adsorbed water and residual solvent) and the thermal decomposition of the sample during heating. The melting point, moisture content, and enthalpy of fusion were determined from the resulting TGA and DSC curves using Universal Analysis 2000 software.

### 2.5. COSMO-RS Analysis

COSMO-RS (Conductor-like Screening Model for Real Solvents) is a predictive thermodynamic method based on quantum chemistry and statistical thermodynamics. It can estimate phase equilibria and thermodynamic properties of fluids and liquid mixtures without relying on experimental data [29]. Its core is the COSMO dielectric continuum solvation model, which places a molecule in a virtual conductor environment with an infinite dielectric constant. Through dielectric polarization, this induces a “screening charge density” on the molecular surface, ultimately described by the σ-profile (sigma-profile) that characterizes local polarity. In this study, the COSMO-RS method was used to predict the dissolution trends of daptomycin in eight pure solvents. All calculations were performed using COSMOtherm 2023. A conformational search for daptomycin was first conducted using COSMOconf 2023. Although gas-phase single-point energy calculations at the BP86/TZVP level were performed on all identified conformers to assess relative stability, only the lowest-energy conformer was selected for subsequent COSMO-RS analyses. The σ-profiles were generated based on the COSMO-RS solvation model using this selected conformer. For the solvent molecule, σ-profiles were directly retrieved from the built-in TZVP-parameterized database. Finally, hydrogen bonding moments, reflecting the hydrogen-bonding capabilities of daptomycin and the solvents, were calculated using the Mixture module in COSMOtherm 2023 [30].

### 2.6. Molecular Dynamics Analysis

Simulations were performed using the GROMACS 2025.3 software package, employing the well-validated Generalized Amber Force Field (GAFF) to describe all intramolecular and intermolecular interactions within the system. The total potential energy of this force field encompasses bonded terms (harmonic potentials for bond stretching and angle bending, Fourier series for dihedral torsions) and non-bonded terms (Lennard–Jones potential for van der Waals forces and Coulomb’s law for electrostatic interactions). Its parameters are derived from the systematic fitting of experimental and quantum chemical calculation data, making it suitable for simulating such complex organic molecular systems. The specific workflow was as follows: The topology file for daptomycin was generated using the sobtoptool, which calculated atomic charges via the AM1-BCC method and assigned GAFF atom types. Organic solvent molecules were also modeled with the GAFF force field; their atomic partial charges were obtained as RESP charges fitted from quantum chemical calculations performed at the B3LYP/6-311G+(d,p) level. Water molecules were represented using the SPC/E model. A single daptomycin molecule was placed at the center of a cubic simulation box. Consistent with recent systematic benchmarking studies on protein simulations, which demonstrate that a minimum solute-to-boundary distance of 1.0 nm is sufficient to prevent finite-size artifacts [31], we selected a conservative offset of 2.5 nm for our calculations. This specific cutoff ensures that no spurious short-range interactions occur between periodic images of the cyclic lipopeptide, thereby guaranteeing the physical validity of the derived thermodynamic properties.

The system first underwent energy minimization (using the steepest descent algorithm followed by the conjugate gradient method, for a total of 10,000 steps) until the maximum force fell below 100 kJ·mol^−1^·nm^−1^. This was followed by a two-step equilibration protocol: 1. NVT equilibration: A 10 ns simulation at 298.15 K, with temperature controlled using the V-rescale thermostat. 2. NPT equilibration: A subsequent 10 ns simulation at 1 bar, with pressure controlled using the Berendsen barostat (isothermal compressibility set to 4.5 × 10^−5^ bar^−1^). Following equilibration, a 100 ns NPT production simulation was conducted using the Verlet integration algorithm (2 fs timestep). A cutoff radius of 1.0 nm was applied for non-bonded interactions, and long-range electrostatic interactions were treated using the Particle Mesh Ewald (PME) method. All bonds involving hydrogen atoms were constrained using the LINCS algorithm (detailed parameters are provided in Supplementary Materials Tables S3–S5). Trajectory analysis included: Hydrogen bond analysis, performed using the GROMACS’s gmx hbondmodule with geometric criteria (donor–acceptor distance ≤ 0.35 nm and donor–hydrogen–acceptor angle ≥ 135°), and Conformational cluster analysis, aimed at identifying dominant conformational states. The latter involved preprocessing the trajectory (removing overall translation and rotation, sampling one frame every 20 ps), followed by constructing a root-mean-square deviation (RMSD) matrix based on backbone heavy atoms (C, N, O). Conformational states were classified using the GROMOS hierarchical clustering algorithm with a cutoff distance of 0.2 nm. This specific threshold was selected because iterative testing demonstrated that 0.2 nm provides optimal resolution for distinguishing relevant conformational substates. Furthermore, this value aligns with the official documentation recommendations for this analysis protocol. In contrast, a larger cutoff of 0.5 nm was empirically found to be insufficient, failing to resolve distinct conformational differences between systems. Using this 0.2 nm cutoff, we quantified the regulatory effect of the solvent environment on the conformational distribution [32,33,34,35,36].

### 2.7. Crystallization Procedure

The crystallization was performed by adjusting the pH of an aqueous daptomycin solution to ~3.5, concentrating it to ~700 g/L, cooling it to 4 °C at 5 °C/h, and then adding the antisolvent (1.0 vol. relative to the initial solution) dropwise at 0.1 vol./h. The resulting precipitate was isolated via filtration, washed, and vacuum-dried at 50 °C to constant weight.

## 3. Thermodynamic Models

The experimentally obtained solubility data were correlated using three thermodynamic models: (1) the modified Apelblat equation, an empirical model describing the temperature dependence of solubility; (2) the Yaws model, a semi-empirical relationship based on a polynomial function; (3) the van’t Hoff equation, which applies to ideal or nearly ideal solutions and expresses the relationship between the mole fraction solubility and temperature (See Supplementary Materials: S1. Thermodynamic models section). To evaluate the accuracy of each model and identify the optimal one, the average relative deviation (ARD) and the root-mean-square deviation (RMSD) were selected as evaluation indicators, calculated according to Equations (2) and (3), respectively [37,38,39,40].

(2) ARD=∑i=1N|x1−x1calx1|N

(3) RMSD=∑i=1N|x1−x1calx1|N

Here, *x*_1_^exp^ denotes the experimentally measured mole fraction of daptomycin, *x*_1_^cal^ denotes the corresponding model-calculated mole fraction from nonlinear regression, and N denotes the number of temperature points per solvent (N = 8 in this study).

## 4. Results and Discussion

### 4.1. Solid–Liquid Equilibrium Data

**Figure 4:**
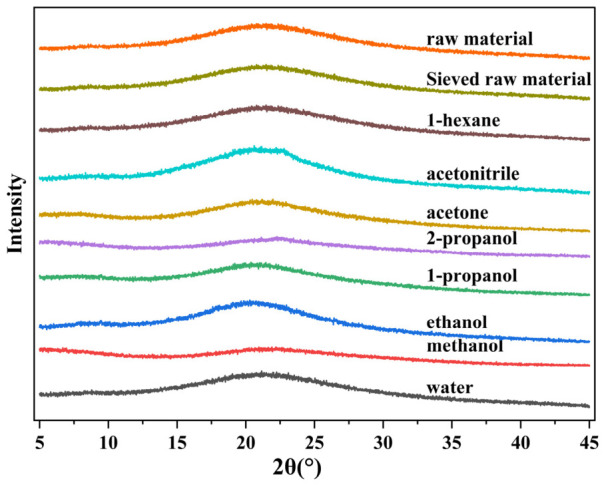
PXRD patterns of daptomycin and samples treated with different solvents during solid–liquid equilibrium.

PXRD analysis was conducted to assess whether solid form transformations occurred in daptomycin during the solid–liquid equilibrium (Figure 4). To evaluate whether gentle grinding treatment could induce a solid-state phase transformation, this study selected two representative solid samples (the as-received drug substance screened powder and the drug substance powder after grinding) and compared their PXRD (Powder X-ray Diffraction) patterns before and after grinding. The results showed that the characteristic diffraction peak positions and intensities remained unchanged. Thus, the gentle grinding procedure used in this study did not induce a significant polymorphic transition. A comparison of the patterns from the post-equilibrium solid residues and the untreated raw material confirmed no detectable polymorphic transitions. The patterns were consistent with a stable amorphous state, showing no evidence of crystalline phase changes that could alter the solid-state structure [41,42].

**Figure 5:**
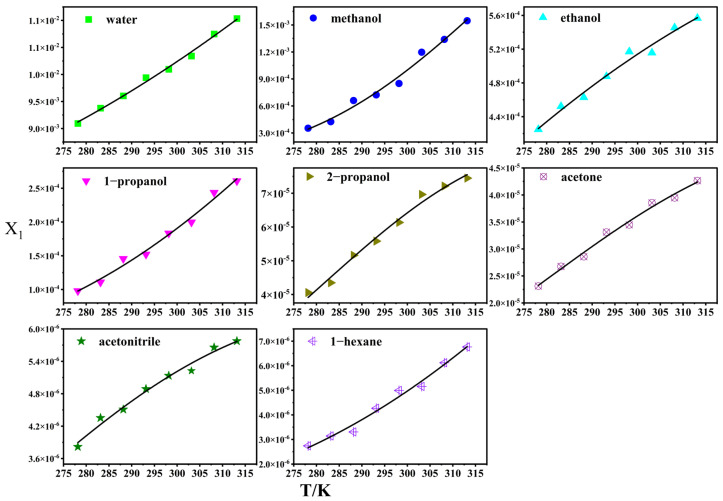
Solubility profiles of daptomycin in various solvents across temperatures (solid lines represent Yaws model correlations).

The equilibrium solubility of daptomycin was experimentally determined in eight pure solvents (water, methanol, ethanol, n-propanol, isopropanol, acetone, acetonitrile, and n-hexane) over the temperature range of 278.15 K to 313.15 K at atmospheric pressure, with measurements taken at 5 K intervals. As illustrated in Figure 5, the solubility of daptomycin increased with rising temperature in all solvents studied, indicating that the dissolution process is endothermic. The solubility values, ranked in descending order, were as follows: water > methanol > ethanol > n-propanol > isopropanol > acetone > acetonitrile > n-hexane.

The experimental solubility data were correlated using three thermodynamic models: the van’t Hoff equation, the modified Apelblat model, and the Yaws model. The corresponding fitting parameters and calculation results are summarized in the support information. The coefficients of determination (R^2^) for the fits of the three models to the experimental data are provided in the Supplementary Information. Most R^2^ values are above 0.98, with many systems exceeding 0.99. This provides statistically robust evidence that all three thermodynamic models exhibit high accuracy in describing the temperature dependence of daptomycin solubility. To evaluate the accuracy of each model, the average relative deviation (ARD) and the root-mean-square deviation (RMSD × 10^5^) were employed as assessment metrics. The Average Relative Deviation (ARD) quantifies the average level of relative error between the predicted values of a model and the experimental data. A smaller ARD value indicates higher average predictive accuracy of the model. Typically, an ARD below 5% is considered to reflect good model fitting and predictive capability. The Root-Mean-Square Deviation (RMSD) reflects the absolute dispersion of prediction errors and is more sensitive to larger deviations. A smaller RMSD signifies higher overall goodness of fit and greater stability in the model’s predictions. The ARD values, in ascending order, were as follows: Yaws (1.56%) < modified Apelblat (1.81%) < van’t Hoff (1.99%). The RMSD values, also in ascending order, were: Yaws (0.79) < van’t Hoff (1.01) < modified Apelblat (2.15). All ARD values of the models are significantly below 5%, indicating that all three models correlate well with the experimental data. Among them, the Yaws model exhibits the smallest deviations in both metrics, with an ARD value of 1.56%, demonstrating that the average relative deviation between its predicted values and the experimental data is extremely low, confirming its excellent fitting accuracy. Based on these two indicators, the Yaws model demonstrated the highest predictive accuracy for solubilities of daptomycin, suggesting that it is appropriate for optimizing industrial crystallization processes (detailed parameters are provided in Supplementary Materials Tables S1 and S2) [43].

### 4.2. COSMO-RS Analysis of Dissolution Mechanism

**Figure 6:**
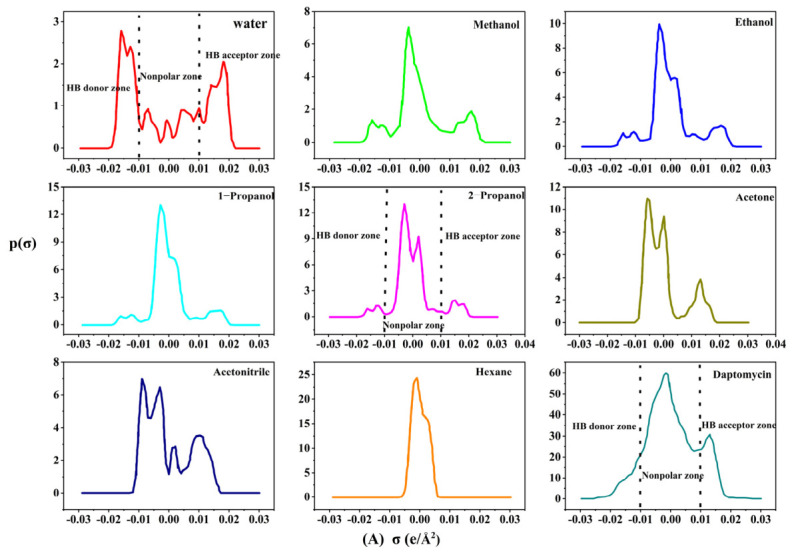

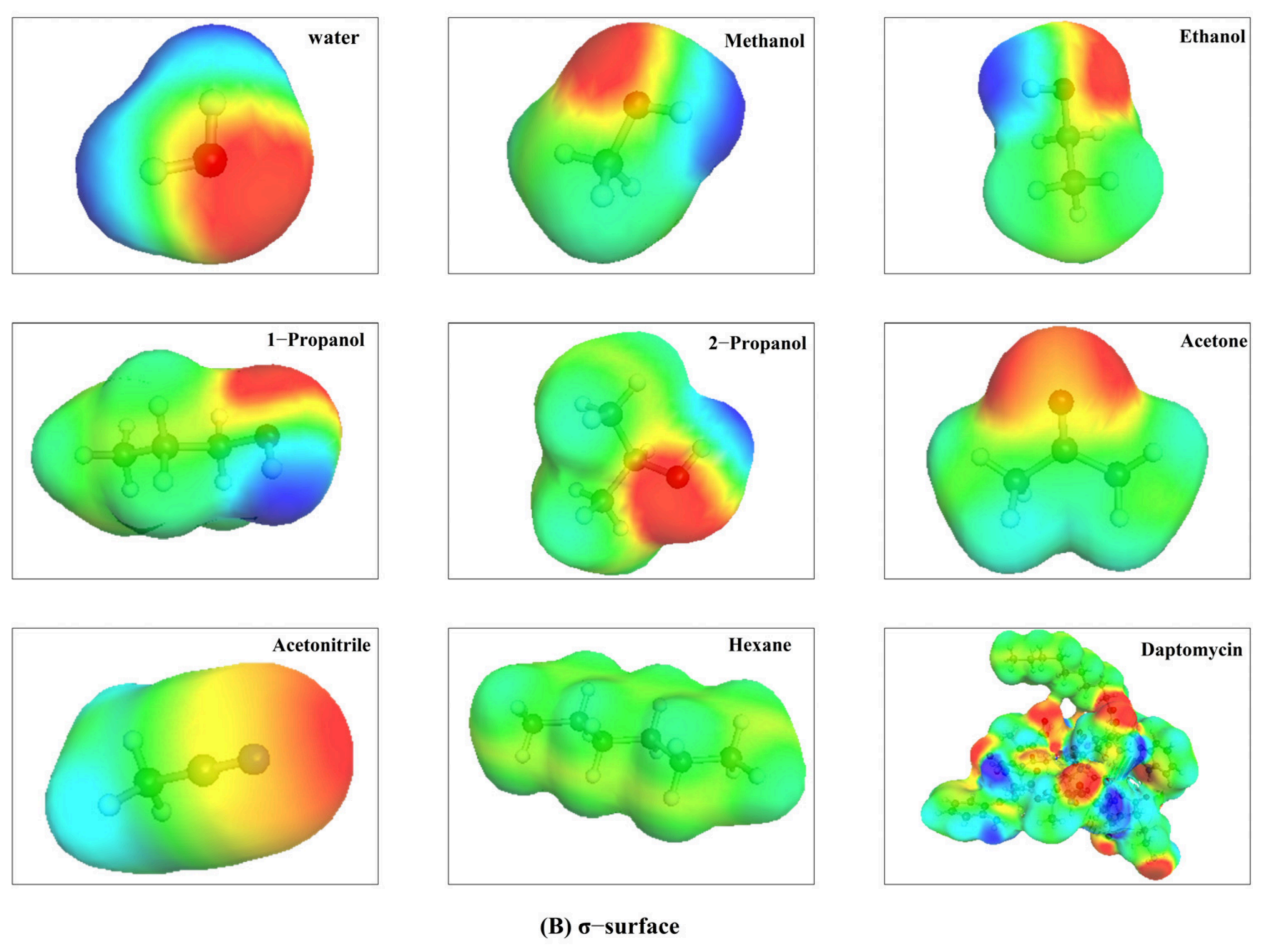
(**A**) σ-Profiles of solvents and daptomycin; (**B**) σ-surfaces of solvents and daptomycin.

The σ-profile and σ-surface of daptomycin were computationally characterized to elucidate the molecular interactions governing its solubility behavior. Quantum chemical calculations were performed using BIOVIA TmoleX (version 23.0.0) at the TZVP level to generate the necessary electronic structure data of daptomycin. The resulting σ-profile, which maps the probability distribution of the screening charge density (σ), is shown in Figure 6. This profile is divided into three key interaction regions: the hydrogen-bond donor region (σ < −0.01 e·Å^−2^), the hydrogen-bond acceptor region (σ > 0.01 e·Å^−2^), and the nonpolar region (−0.01 ≤ σ ≤ 0.01 e·Å^−2^). Analysis of the σ-profile reveals a pronounced peak in the hydrogen-bond acceptor region, reflecting the abundance of electronegative atoms (carbonyl oxygens and carboxylates) on the daptomycin surface. Although the hydrogen-bond donor region does not exhibit a sharp peak, it possesses a distinct integral area under the curve, which corresponds to the presence of amide N-H groups. This pattern indicates a highly polar molecular surface, consistent with the molecular structure of daptomycin, which contains 43 hydrogen-bond acceptors (17 nitrogen and 26 oxygen atoms) and 22 hydrogen-bond donors. The corresponding σ-surface visualization, generated as an isosurface at σ = ±0.01 e·Å^−2^, provides a spatial representation of the screening charge density. The surface exhibits a distinct polarity partitioning, with blue areas (representing local positive charge, hydrogen-bond donors) and red areas (representing local negative charge, hydrogen-bond acceptors) prominently distributed across the molecular surface. This visualization further corroborates the strongly polar character inferred from the σ-profile [44].

COSMOtherm 2023’s Mixture module quantified hydrogen-bonding strengths (Moment_HBacc: acceptor moment, Moment_HBdon: donor moment) for 1:1 daptomycin–solvent systems (Table 2). Since Σ(Moment_HBacc + Moment_HBdon) for all solvents was lower than the value for daptomycin (53.51), solvents were ranked by Σ(Moment_HBacc + Moment_HBdon), yielding the following order: water > methanol > ethanol > 1-propanol > 2-propanol > acetone > acetonitrile > hexane. This computational ranking is in strong concordance with experimental solubility data under identical conditions. From the perspective of hydrogen bonding, acetone acts as a strong hydrogen-bond acceptor. Coupled with its inherent non-polar character and absence of intramolecular hydrogen bonding, it effectively stabilizes the conformation of daptomycin.

### 4.3. Molecular Dynamics (MD) Analysis

#### 4.3.1. Force Field Validation by Matching Simulated and Experimental Densities

**Figure 7:**
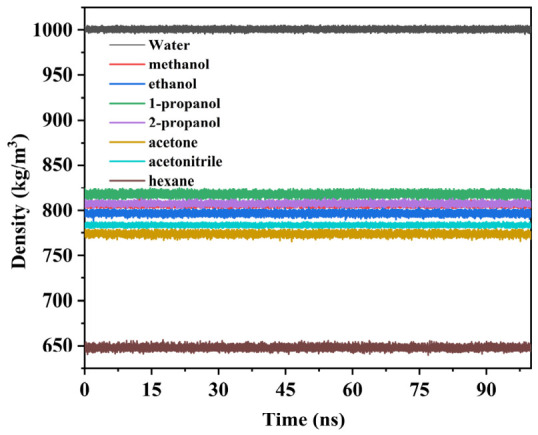
Density fluctuation profile of the daptomycin simulated system in different pure solvents box.

Molecular dynamics (MD) simulations of eight pure solvents at 298.15 K yielded equilibrium densities in excellent agreement with experimental values, with absolute deviations generally below 3% (Table 3, Figure 7). Specific deviations were notably small for water (−0.08%), acetone (−1.26%), and acetonitrile (−0.29%), while a slightly higher deviation was observed for 2-propanol (2.87%). The close correlation validates the GAFF force field and the simulation parameters for these solvents, confirming that the simulations reliably reproduce liquid-phase behavior. The minor deviations for branched alcohols may stem from complexities in modeling their geometry and van der Waals interactions, but remain acceptable. This accuracy ensures subsequent structural and dynamic analyses (hydrogen-bonding, spatial distributions) are physically meaningful and provides a solid basis for interpreting daptomycin solvation at the molecular level [45].

#### 4.3.2. Analysis of Electrostatic Interaction Between Daptomycin and Solvents

Molecular dynamics simulations reveal a correlation between the calculated Coulomb interaction energies of daptomycin and its experimental solubility order across eight solvents: water > methanol > ethanol > n-propanol > isopropanol > acetone > acetonitrile > n-hexane. Table 4 presents the interaction energy per mole of daptomycin with the solvent in different solvents. Here, the Error Estimate refers to the statistical error in calculating the Coulomb interaction energy, where a smaller value indicates a more reliable result; the RMSD of the Energy represents the fluctuation amplitude of the energy value during the simulation, with a smaller value indicating more stable energy sampling, and the Total Drift denotes the cumulative shift in energy over simulation time, where a smaller absolute value suggests better simulation energy conservation and, consequently, more credible results. Water exhibits the strongest interaction energy (−1126.61 kJ/mol), consistent with its highest solubility, attributable to its amphoteric nature, enabling effective solvation of daptomycin’s polar groups. The interaction energy decreases with increasing alkyl chain length in alcohols due to enhanced steric hindrance. The negligible Coulomb interaction in n-hexane (−2.17 kJ/mol) explains its extremely low solubility [46].

#### 4.3.3. Hydrogen Bonding of Daptomycin with Solvents

**Figure 8:**
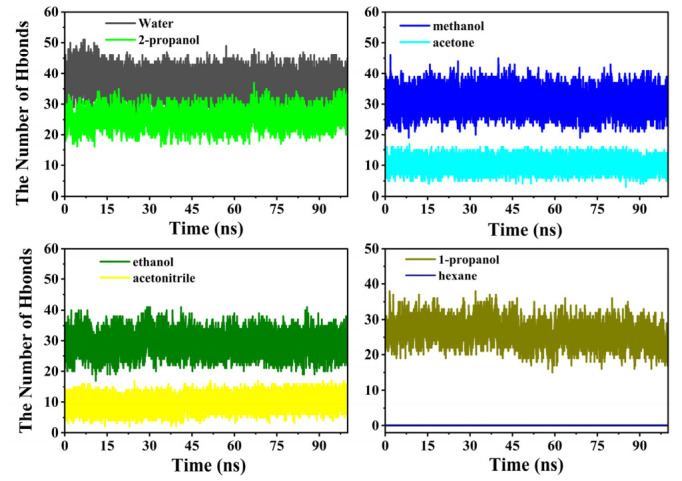
Time-Dependent Hydrogen Bond Number of Daptomycin in Various Solvents.

Hydrogen bonding, as a type of electrostatic interaction, critically governs the solubility, stability, and conformational maintenance of solutes in solvents. The number and dynamic behavior of hydrogen bonds directly reflect the strength of solvent–solute interactions. To verify the convergence of the hydrogen-bond analysis, we monitored the evolution of the hydrogen-bond count as a function of simulation time. As shown in Figure 8, in all solvent systems the number of hydrogen bonds reached a stable plateau rapidly upon initiation and subsequently exhibited only minor fluctuations about the equilibrium value. The standard deviations of the hydrogen-bond counts were all below 4, which further corroborates the convergence of the system.

As illustrated in Figure 8 and summarized in Table 5, daptomycin exhibits significantly distinct hydrogen-bonding patterns across different solvents, which correlate strongly with solvent polarity. In aqueous solution, the number of hydrogen bonds remains high (approximately 30–40) with relatively mild fluctuations, indicating a dynamically stable hydrogen-bonding network. As solvent polarity decreases, both the average number and stability of hydrogen bonds decline. In aprotic polar solvents such as acetone and acetonitrile, a pronounced reduction in hydrogen bond count (around 10) is observed, attributable to their inability to act as hydrogen bond donors. In n-hexane, hydrogen bonding is virtually negligible. These findings confirm that the solubility and conformational stability of daptomycin are highly dependent on the dynamic persistence of hydrogen bonds with solvent molecules [47].

#### 4.3.4. Computational Screening of Antisolvent Crystallization Solvents Using Daptomycin Conformational Clustering

**Figure 9:**
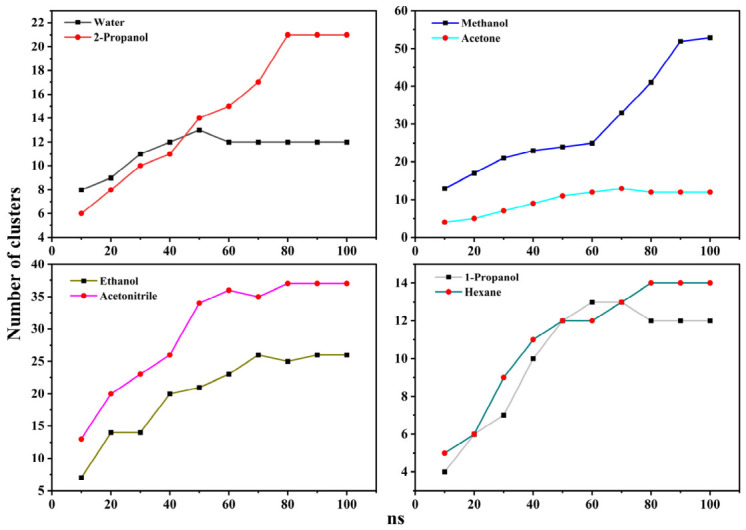
Cumulative number of conformational clusters for daptomycin across different solvents over simulation time.

Figure 9 illustrates the relationship between the number of conformational clusters and simulation time. With the exception of daptomycin in methanol, which did not fully reach a convergence plateau, the cluster counts for all other systems remained stable after 80 ns. Consequently, the number of clusters in the stable region was employed as the primary criterion for selecting crystallization solvents. Although the methanol system had not fully converged, its cluster count was the highest among all solvents tested; thus, it does not affect the relative ranking of solvents regarding their ability to restrict conformational diversity, supporting the validity of this descriptor for identifying suitable crystallization systems.

Conformational clustering analysis, which groups structurally similar conformations from molecular dynamics (MD) trajectories into representative clusters, serves as a powerful tool for deciphering the structural landscape of highly flexible molecules such as daptomycin. This approach elucidates the conformational preferences of the molecule across different solvents. The number of distinct clusters directly reflects the degree of conformational diversity: a lower count indicates fewer dominant conformational states—a phenomenon termed “conformational pre-organization”—signifying a more concentrated distribution and reduced diversity. This pre-organization minimizes the entropic penalty associated with the transition from a disordered to an ordered crystalline state, thereby facilitating nucleation, crystal growth, and enhancing the stability of the species (Table 6, with the NPT output conformation used as the reference structure). Details regarding the RMSD variation in daptomycin during the simulations are provided in Supplementary Information, Figure S1 [48].

To validate this analysis as a predictive tool for antisolvent selection, we systematically screened six solvents. The primary goal was to correlate the computed conformational distribution of daptomycin (indicated by cluster counts) with its experimental crystallization outcome. Solvent performance was rigorously evaluated via PXRD, with well-defined diffraction patterns as the key indicator of successful crystal formation. This approach directly tested the central hypothesis: whether solvents promoting conformational pre-organization (lower cluster counts) are more likely to yield high-quality crystalline material.

**Figure 10:**
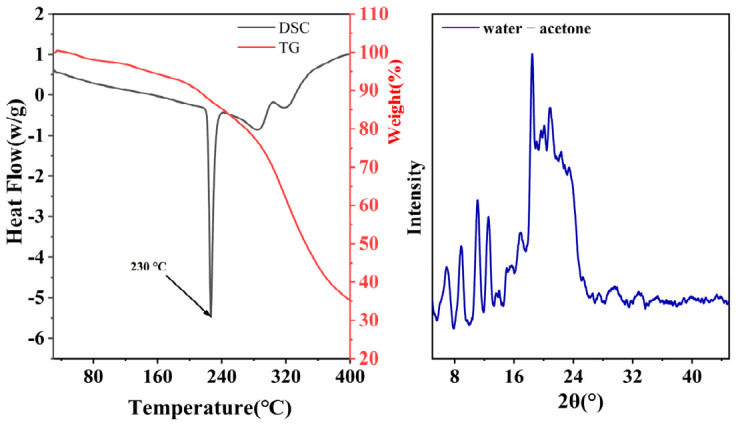
TG-DSC and PXRD patterns of daptomycin crystalline powder obtained from the aqueous solution crystallization using acetone as the antisolvent.

**Figure 11:**
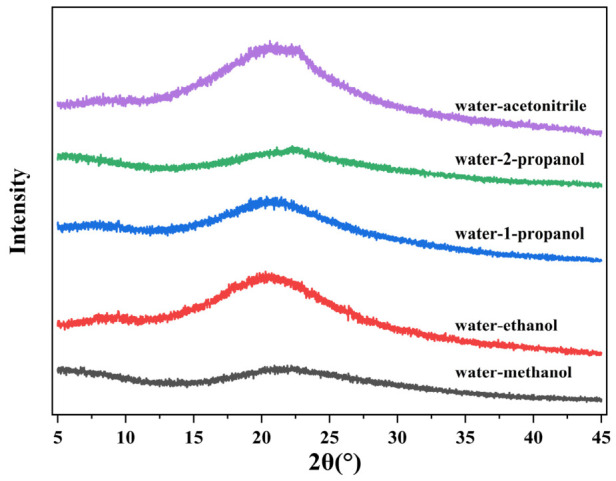
PXRD patterns of daptomycin powder obtained from the aqueous solution crystallization using other antisolvents.

Of the solvents tested, acetone demonstrated unique crystallization-inducing efficacy, being the only solvent that yielded a powder with a well-defined PXRD pattern. This marks the first successful crystallization of daptomycin in an acetone–water system. Its moderate polarity minimizes disruption to the solute’s native conformation, while its good miscibility with water facilitates a solution environment conducive to controlled supersaturation and orderly molecular assembly. PXRD analysis of the solid product (Figure 10) revealed distinct diffraction peaks at approximately 2θ = 6.98°, 8.95°, 11.04°, 12.58°, and 18.46°, indicating significant crystallization compared to the amorphous material (Figure 11). Based on Bragg’s law, the corresponding interplanar spacings (d-spacings) calculated from these characteristic peak positions are 12.66 Å, 9.88 Å, 8.01 Å, 7.03 Å, and 4.8 Å, respectively, providing partial basis for inferring possible unit cell parameters and molecular packing modes. However, as evident from the PXRD patterns, peak overlap and aggregation are observed within the 2θ range of 16°to 24°, indicating that the material prepared via this process is, strictly speaking, partially crystalline. Further studies are still required to obtain fully ordered, crystalline daptomycin. Concurrent TG-DSC analysis (Figure 10) revealed a sharp endothermic peak near 230 °C, characteristic of a crystalline phase transition. This transition, however, was accompanied by significant mass loss, attributed to the evaporation of residual water and the onset of thermal decomposition. The concurrence of these events indicates limited thermal stability of the obtained solid form [41]. Collectively, these results validate the solvent-modulated conformational diversity strategy from both crystallographic and thermodynamic perspectives, offering key experimental evidence for the solid-form development of complex, flexible molecules (Figure 10 and Figure 11).

Clustering analysis revealed a clear trend: solvents that failed to produce crystalline powders were associated with higher conformational cluster counts, supporting the hypothesis that greater conformational dispersion impedes the molecular ordering required for crystallization. Although 1-propanol constituted an exception—yielding a low cluster count (12) comparable to the successful solvent acetone, yet without forming crystalline material—this anomaly may be attributed to specific crystallization kinetics or limitations in the clustering resolution. Overall, the strong correlation demonstrates that MD simulations combined with conformational clustering provide valuable insight into a solute’s conformational behavior across solvents. This integrated computational approach shows significant potential as a tool for preliminary assessment of how solvents influence a molecule’s propensity for ordered assembly, offering a rational strategy for initial antisolvent screening in crystallization process development.

## 5. Conclusions

This study systematically elucidated the crystallization mechanism of daptomycin by integrating macroscopic solubility measurements, thermodynamic modeling, and multi-scale simulations. Experimentally measured solubility in eight solvents increased monotonically with temperature, confirming the endothermic nature of the dissolution process. The solubility trend (water > methanol > ethanol > n-propanol > isopropanol > acetone > acetonitrile > n-hexane) indicated that solvent polarity and hydrogen-bonding capacity are the key interactions governing its dissolution, providing a molecular-level qualitative explanation for the macroscopic dissolution phenomenon. Among the models tested, the Yaws model achieved the most accurate solubility prediction (average relative deviation ARD = 1.56%), validating its reliability for guiding crystallization process design.

Molecular dynamics simulations further revealed that solvent-modulated conformational flexibility is the core factor determining crystallization success. In acetone, which successfully induced crystallization, daptomycin was confined to a limited number of dominant conformational states (12 clusters). This “conformational pre-organization” effectively reduced the entropic barrier for the ordered molecular packing required for nucleation, thereby promoting crystal formation. In contrast, in solvents like methanol, which yielded only amorphous precipitates, the molecules exhibited higher conformational flexibility (e.g., 53 clusters), and the substantial conformational entropy impeded ordered assembly. Notably, n-propanol constituted a critical exception—although it similarly restricted conformations to 12 clusters, it failed to induce crystallization. This phenomenon clearly demonstrates that a “low number of conformational clusters” (i.e., restricted conformational flexibility) is a necessary but not sufficient condition for promoting crystallization. This exception suggests that, beyond conformational flexibility, kinetic factors such as solvent steric hindrance, interfacial adsorption characteristics, and solution viscosity may also play crucial roles in the eventual realization of crystallization. This points the way for future research: moving beyond single descriptors to conduct comprehensive multi-parameter assessments.

Based on the mechanistic insights above, we constructed a rational and efficient solvent screening framework, with the core steps comprising the following: (1) Thermodynamic Preliminary Screening: Combining experimental solubility and COSMO-RS predictions to rapidly assess the dissolution capacity and interaction nature of solvent systems, thereby identifying a pool of potential antisolvent candidates. (2) Screening via Kinetic Descriptor: Utilizing molecular dynamics simulations to extract the “number of conformational clusters” as a key descriptor, enabling the rapid identification of preferred antisolvents (such as acetone) that significantly restrict conformational flexibility and are expected to lower the nucleation barrier. The final process conditions are then determined through experimental validation.

This strategy provides a rational screening pathway from microscopic mechanisms to macroscopic processes for the crystallization development of complex macromolecules with high flexibility and multiple dissociation sites. It can significantly reduce the blindness and resource consumption associated with traditional trial-and-error approaches.

## Figures and Tables

**Table 1:** Description of the chemicals used in this paper.

Chemicals	CAS NO.	Molecular Formula	Molecular Weight (g/mol)	Mass Fraction Purity (%)
Daptomycin	103060-53-3	C_72_H_101_N_17_O_26_	1620.67	≥98
Methanol	67-56-1	CH_4_O	32.04	≥99.7
Ethanol	64-17-5	C_2_H_6_O	46.07	≥99.7
1-Propanol	71-23-8	C_3_H_8_O	60.10	≥99.7
2-Propanol	67-63-0	C_3_H_8_O	60.10	≥99.7
Acetonitrile	75-05-8	C_2_H_3_N	41.05	≥99.7
n-hexane	110-54-3	C_6_H_14_	86.18	≥99.7
Acetone	67-64-1	C_3_H_6_O	58.08	≥99.7

**Table 2:** Calculated hydrogen bond (HB) donor and acceptor moment indices for solvent-daptomycin interactions.

Solvent	Moment_HBacc	Moment_HBdon	Moment_HBacc + Moment_HBdon
Water	5.70	3.85	9.55
Methanol	4.17	1.88	6.05
Ethanol	4.07	1.65	5.72
1-Propanol	3.79	1.61	5.40
2-Propanol	3.96	1.29	5.25
Acetone	2.77	0.00	2.77
Acetonitrile	1.34	0.00	1.34
n-hexane	0.00	0.00	0.00

**Table 3:** Validation of Force Field via Simulated vs. Experimental Density.

Pure Solvent	Theoretical Simulated Density @25 °C	Experimental Density @25 °C	Deviation
Water	0.999	1	−0.08%
Methanol	0.807	0.791	2.02%
Ethanol	0.794	0.785	1.19%
1-Propanol	0.818	0.803	1.82%
2-Propanol	0.808	0.785	2.87%
Acetone	0.774	0.784	−1.26%
Acetonitrile	0.784	0.786	−0.29%
n-hexane	0.650	0.6594	−1.39%

**Table 4:** Intermolecular Electrostatic Interactions of Daptomycin with Solvents.

Solvent	Coulombic Short-Range Interaction Energy (KJ/mol)	Error Estimate	Root Mean Square Deviation (RMSD)	Total Drift
Daptomycin@Water	−1126.61	5.00	72.79	24.71
Daptomycin@Methanol	−932.51	4.20	75.40	0.16
Daptomycin@ Ethanol	−846.26	11.00	73.67	78.66
Daptomycin@1-Propanol	−834.85	14.00	75.23	100.12
Daptomycin@2-Propanol	−762.33	7.30	63.31	−44.05
Daptomycin@ Acetone	−369.15	2.30	33.07	−12.81
Daptomycin@Acetonitrile	−348.40	5.30	33.15	−24.53
Daptomycin@ n-hexane	−2.17	0.04	1.96	0.12

**Table 5:** Hydrogen Bonding in Daptomycin–Solvent Molecular Systems.

Solvent	Average Number of Hydrogen Bonds	Standard Deviation of Hydrogen Bonds
Water	36.67	3.11
Methanol	31.12	3.02
Ethanol	29.24	2.93
1-Propanol	26.35	2.86
2-Propanol	25.42	2.52
Acetone	10.45	1.70
Acetonitrile	9.86	2.00
n-hexane	0	0

**Table 6:** Conformational fluctuation range and the number of conformational clusters of daptomycin in different solvents.

Solvent	RMSD Fluctuation Range (nm)	Number of Conformational Clusters
Water	0.0313~ 0.5931	16
Methanol	0.0340~ 0.6633	53
Ethanol	0.0308~ 0.5300	26
1-Propanol	0.0299~ 0.6121	12
2-Propanol	0.0298~ 0.5809	21
Acetone	0.0342~ 0.4958	12
Acetonitrile	0.0291~ 0.6624	37
n-hexane	0.0316~ 0.4726	14

## Data Availability

The original contributions presented in this study are included in the article/Supplementary Materials. Further inquiries can be directed to the corresponding author.

## Supplementary Materials

The following supporting information can be downloaded at: https://www.mdpi.com/article/10.3390/pharmaceutics18060657/s1, Figure S1: Temporal evolution of RMSD for daptomycin across different solvent conditions; Table S1: Experimental and calculated mole fraction solubility (×1) of daptomycin in various solvents; Table S2: Correlation model-specific parameters for daptomycin solubility in selected solvents; Table S3: The topology file for daptomycin; Table S4: The MDP file for the NVT ensemble; Table S5: The MDP file for the NPT ensemble.

## References

[1] Mcpherson A., Cudney B. (2006). Searching for silver bullets: An alternative strategy for crystallizing macromolecules. J. Struct. Biol..

[2] Vekilov P.G. (2010). The two-step mechanism of nucleation of crystals in solution. Nanoscale.

[3] van de Streek J., Dietrich H., Firaha D., Ludwig M., Ovchinnikov A., Sasikumar K., DiPasquale A.G., Iuzzolino L., Kelly A.W., Lafarguette P. (2025). A Conceptual Framework for the Crystallizability of Organic Compounds. J. Am. Chem. Soc..

[4] Rychkov D.A., Hunter S., Kovalskii V.Y., Lomzov A.A., Pulham C.R., Boldyreva E.V. (2016). Towards an understanding of crystallization from solution. DFT studies of multi-component serotonin crystals. Comput. Theor. Chem..

[5] Derewenda Z.S. (2004). Rational protein crystallization by mutational surface engineering. Structure.

[6] Asherie N. (2004). Protein crystallization and phase diagrams. Methods.

[7] Goldschmidt L., Cooper D.R., Derewenda Z.S., Eisenberg D. (2007). Toward rational protein crystallization: A Web server for the design of crystallizable protein variants. Protein Sci..

[8] Moon A.F., Mueller G.A., Zhong X., Pedersen L.C. (2010). A synergistic approach to protein crystallization: Combination of a fixed-arm carrier with surface entropy reduction. Protein Sci..

[9] McPherson A. (2004). Introduction to protein crystallization. Methods.

[10] Chayen N.E., Saridakis E. (2008). Protein crystallization: From purified protein to diffraction-quality crystal. Nat. Methods.

[11] Ho S.W., Jung D., Calhoun J.R., Lear J.D., Okon M., Scott W.R.P., Hancock R.E.W., Straus S.K. (2008). Effect of divalent cations on the structure of the antibiotic daptomycin. Eur. Biophys. J..

[12] Yunxia S., Rui H., Junbo H.G. (2024). Practical techniques for protein crystallization: Additive assistance and external field intensification. CrystEngComm.

[13] Savchenko M., Hurtado M., Lopez-Lopez M.T., Rus G., de Cienfuegos L.Á., Melchor J., Gavira J.A. (2022). Lysozyme crystallization in hydrogel media under ultrasound irradiation. Ultrason. Sonochem..

[14] Jiao J., Li H., Xie W., Zhao Y., Lin C., Jiang J., Wang L. (2023). Host–guest system of a phosphorylated macrocycle assisting structure determination of oily molecules in single-crystal form. Chem. Sci..

[15] Sharma H., Kalita B.K., Pathak D., Sarma B. (2024). Low Molecular Weight Supramolecular Gels as a Crystallization Matrix. Cryst. Growth Des..

[16] Båth P., Banacore A., Börjesson P., Bosman R., Wickstrand C., Safari C., Dods R., Ghosh S., Dahl P., Ortolani G. (2022). Lipidic cubic phase serial femtosecond crystallography structure of a photosynthetic reaction centre. Acta Crystallogr. Sect. D Struct. Biol..

[17] Mccue C., Girard H.L., Varanasi K.K. (2023). Enhancing Protein Crystal Nucleation Using In Situ Templating on Bioconjugate-Functionalized Nanoparticles and Machine Learning. ACS Appl. Mater. Interfaces.

[18] Santos R.D., Romo M.J., Roque A.C.A., Carvalho A.L. (2021). Magnetic Particles used in a New Approach for Designed Protein Crystallization. CrystEngComm.

[19] Jones H.G., Wrapp D., Gilman M.S.A., Battles M.B., Wang N., Sacerdote S., Chuang G.-Y., Kwong P.D., McLellan J.S. (2019). Iterative screen optimization maximizes the efficiency of macromolecular crystallization. Acta Crystallogr. Sect. F Struct. Biol. Commun..

[20] Zaykovskaya A., Amano B., Louhi-Kultanen M. (2024). Influence of Viscosity on Variously Scaled Batch Cooling Crystallization from Aqueous Erythritol, Glucose, Xylitol, and Xylose Solutions. Cryst. Growth Des..

[21] Ticonachambi J., Choquesillolazarte D., Cuffini S.L., Infantes L. (2025). Comparative Assessment of Statistical and Thermodynamic Prediction Methods for Solvate Formation: A Case Study with Curcumin and Its Derivatives. Cryst. Growth Des..

[22] Madani M., Tarakanova A. (2024). Protein dynamics inform protein structure: An interdisciplinary investigation of protein crystallinity. Matter.

[23] Brown S.J., Ryan T.M., Drummond C.J., Greaves T.L., Han Q. (2024). Lysozyme aggregation and unfolding in ionic liquid solvents: Insights from small angle X-ray scattering and high throughput screening. J. Colloid Interface Sci..

[24] Taylor S.D., Moreira R. (2025). Daptomycin: Mechanism of action, mechanisms of resistance, synthesis and structure-activity relationships. Prog. Mol. Biol. Transl. Sci..

[25] Fiore M., Alfieri A., Fiore D., Iuliano P., Spatola F.G., Limone A., Pezone I., Leone S. (2025). Use of Daptomycin to Manage Severe MRSA Infections in Humans. Antibiotics.

[26] Tanaka R., Kai M., Goto K., Ohchi Y., Yasuda N., Tatsuta R., Kitano T., Itoh H. (2021). High-throughput and wide-range simultaneous determination of linezolid, daptomycin, and tedizolid in human plasma using ultra-performance liquid chromatography coupled to tandem mass spectrometry. J. Pharm. Biomed. Anal..

[27] Li M., Gao Z., Li Z., Wang Z., Zhou R., Wang B. (2020). Determination and correlation of solubility of 4,4-difluorobenzophenone in pure and binary mixed solvents and thermodynamic properties of solution. J. Mol. Liq..

[28] Reinle-Schmitt M., Jung D.Š., Morin M., Costa F., Casati N., Gozzo F. (2023). Exploring high-throughput synchrotron X-Ray powder diffraction for the structural analysis of pharmaceuticals. Int. J. Pharm. X.

[29] Klamt A., Eckert F., Hornig M., Beck M.E., Bürger T. (2002). Prediction of aqueous solubility of drugs and pesticides with COSMO-RS. J. Comput. Chem..

[30] Klamt A., Eckert F., Arlt W. (2010). COSMO-RS: An alternative to simulation for calculating thermodynamic properties of liquid mixture. Annu. Rev. Chem. Biomol. Eng..

[31] Kordonskaya Y.V., Garipov I.F., Timofeev V.I., Marchenkova M.A., Dyakova Y.A., Pisarevsky Y.V., Kovalchuk M.V. (2024). Minimum Acceptable Simulation Box Size Based on a Comparison of the Stability of Lysozyme Oligomers Using Molecular Dynamics. Nanobiotechnol. Rep..

[32] Abraham M.J., Murtola T., Schulz R., Páll S., Smith J.C., Hess B., Lindahl E. (2015). GROMACS: High-performance molecular simulations through multi-level parallelism from laptops to supercomputers. SoftwareX.

[33] Kutzner C., Páll S., Fechner M., Esztermann A., de Groot B.L., Grubmüller H. (2019). More Bang for Your Buck: Improved use of GPU Nodes for GROMACS 2018. J. Comput. Chem..

[34] Lu T., Chen F. (2012). Multiwfn: A multifunctional wavefunction analyzer. J. Comput. Chem..

[35] Lu T. Sobtop, Version 1.0(dev5). http://sobereva.com/soft/Sobtop.

[36] Wang J., Wolf R.M., Caldwell J.W., Kollman P.A., Case D.A. (2004). Development and Testing of a General AMBER Force Field. J. Comput. Chem..

[37] Cui C., Wu H., Sadowski G., Ji Y. (2024). Solubility Measurement and Thermodynamic Modeling of Bifendate in 13 Pure Solvents at Temperatures from 293.15 to 333.15 K. J. Chem. Eng. Data.

[38] Apelblat A., Manzurola E. (1999). Solubilities of o-acetylsalicylic, 4-aminosalicylic, 3,5-dinitrosalicylic, and p-toluic acid, and magnesium-DL-aspartate in water from T = (278 to 348) K. J. Chem. Thermodyn..

[39] Wang L., Chen D., Yu Y., Li H. (2020). Solubility of Octahydro-1,3,5,7-tetranitro-1,3,5,7-tetrazocine in 12 Pure Organic Solvents from 298.15 to 358.15 K. J. Chem. Eng. Data.

[40] Ji W.Q., Meng Q., Ding L., Wang F., Dong J., Zhou G., Wang B. (2016). Measurement and correlation of the solubility of caffeic acid in eight mono and water + ethanol mixed solvents at temperatures from (293.15 to 333.15) K. J. Mol. Liq..

[41] Lee H.E. (2014). A practical guide to pharmaceutical polymorph screening & selection. Asian J. Pharm. Sci..

[42] Wesolowski M., Leyk E. (2023). Coupled and Simultaneous Thermal Analysis Techniques in the Study of Pharmaceuticals. Pharmaceutics.

[43] Bouillot B., Spyriouni T., Teychené S., Biscans B. (2017). Solubility of pharmaceuticals: A comparison between SciPharma, a PC-SAFT-based approach, and NRTL-SAC. Eur. Phys. J. Spec. Top..

[44] Arrad M., Thomsen K., Müller S., Smirnova I. (2024). Thermodynamic modeling using extended UNIQUAC and COSMO-RS-ES models: Case study of the cesium nitrate-water system over a large range of temperatures. Fluid Phase Equilib..

[45] Li J., Amador C., Wilson M.R. (2024). Computational predictions of interfacial tension, surface tension, and surfactant adsorption isotherms. PCCP.

[46] Hao X., Xue X., Zhou J., Zhang X., Li X., Zeng Q., Ma Q., Zhao Y., Wang C., Luo S. (2025). Experimental Evidence for Double Intermolecular Coulombic Decay in Bio-Relevant Molecular Dimers. Phys. Rev. Lett..

[47] Li X., Xu D., Yang J., Yan Z., Luo T., Li X., Zhang Z., Wang X. (2021). Utilization of FBRM and PVM to analyze the effects of different additives on the crystallization of ammonium dihydrogen phosphate. J. Cryst. Growth.

[48] Wang D., Sang J., Wang M., Luo D., Han D., Zhang B., Wang J., Gong J. (2025). Direct crystallization resolution of chiral compounds assisted by chiral ionic liquids through conformational matching. Sep. Purif. Technol..

