# Supplementary material for: Conformational Diversity-Driven Crystallization of Daptomycin: A Multi-Scale Approach with Experimental Validation
Source: Pharmaceutics. 2026 May 27;18(6):657. doi: 10.3390/pharmaceutics18060657 (PMC13304980; doi:10.3390/pharmaceutics18060657)
Supplement: Supplementary file 1 [file pharmaceutics-18-00657-s001.zip › pharmaceutics-4285431-supplementary.pdf]

pharmaceutics

MDPI

Supplementary Materials: Conformational Diversity-Driven Crystallization of Daptomycin: A Multi-Scale Approach with Experimental Validation

Qingshi Wen, Ke Zhang, Li Huang, Shuyang Zhou, Hanjie Ying and Pengpeng Yang *

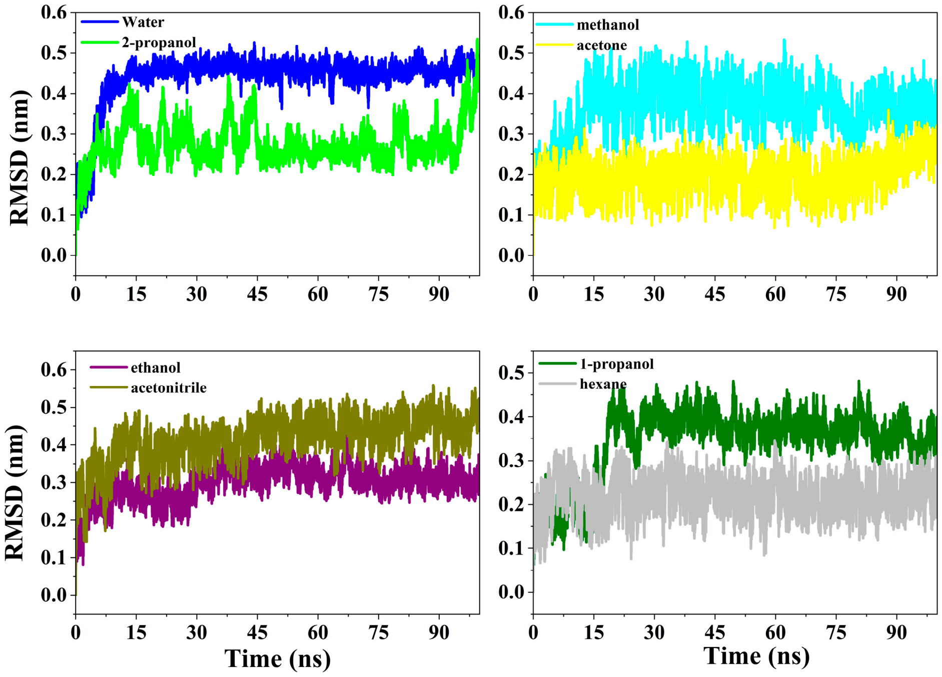

Figure S1. Temporal evolution of RMSD for daptomycin across different solvent conditions.

S1. Thermodynamic models

S1.1. Modified Apelblat model

The modified Apelblat equation, a semi-empirical model widely employed to correlate the temperature dependence of solid solubility in liquids, demonstrates high predictive accuracy in solid-liquid equilibrium systems. This model is expressed as follows:

$$\ln x_1 = A + \frac{B}{T} + C \ln T \quad (1)$$

Here, x_1 represents the mole fraction solubility of the solute, T is the absolute temperature in Kelvin, and A, B, and C are model parameters. Owing to its simple form, clearly interpretable parameters, and high correlation accuracy, the modified Apelblat equation has become an important tool for solubility modeling in solid-liquid equilibrium studies.

S1.2. Yaws model

From a thermodynamic perspective, the Yaws model captures the relationship between mole fraction solubility (x_1) and absolute temperature (T) for solids in equilibrium with their liquid solutions. This semi-empirical correlation, formulated in Equation (5), has proven effective for high-accuracy solubility prediction of organic compounds. The

 https://doi.org/10.3390/pharmaceutics18060657

model parameters A_1, B_1, and C_1 are unique to each solute-solvent system and collectively govern the quantitative dependence of solubility on temperature.

$$\ln x_1 = A_1 + \frac{B_1}{T} + \frac{C_1}{T^2} \quad (2)$$

S1.3. Van´t Hoff equation

$$\ln(x_1) = -\frac{\Delta H_{\text{sol}}^0}{R} \cdot \left(\frac{1}{T} - \frac{1}{T_a}\right) - \frac{\Delta G_{\text{sol}}^0}{RT_a} \quad (3)$$

$$T_a = \frac{N}{\sum_{i=1}^{N} 1/T} \quad (4)$$

$$\Delta G_{\text{sol}}^0 = -RT_a * int\,e\,rcept \quad (5)$$

$$\Delta S_{\text{sol}}^0 = \frac{\Delta H_{\text{sol}}^0 - \Delta G_{\text{sol}}^0}{T_a} \quad (6)$$

The dissolution behavior of daptomycin in relation to temperature was analyzed within a quantitative thermodynamic framework based on the Van't Hoff equation. This model is particularly suitable for systems exhibiting ideal or near-ideal solution behavior and establishes a linear relationship between the natural logarithm of the solute mole fraction solubility (ln x_1) and the reciprocal of the absolute temperature (1/T), as given in Equations (6) to (9). Within this model, x_1 represents the equilibrium mole fraction solubility of daptomycin. The apparent standard dissolution Gibbs energy (ΔG_{sol}^0), enthalpy (ΔH_{sol}^0), and entropy (ΔS_{sol}^0) are derived from the model parameters. The absolute temperature is denoted by T (K), while T_a is a reference temperature used in the harmonic approximation. The linear regression of ln x_1 versus (1/T−1/T_a) yields an intercept, and R is the universal gas constant (8.314 J·mol^{-1}·K^{-1}). This analysis provides insight into the thermodynamic driving forces governing the dissolution process.

Table S1. Experimental and calculated mole fraction solubility (x_1) of daptomycin in various solvents.

		Water		
T/K	$10^5x_1^{exp}$	$10^5x_1^{Modified}$	$10^5x_1^{Yaws}$	$10^5x_1^{Van't\ Hoff}$
278.15	909.3186	904.1126	912.0462	907.1566
283.15	937.6785	931.0496	935.5770	934.8685
288.15	960.4127	957.0219	960.3807	962.4214
293.15	994.1020	982.0121	986.4128	989.8052
298.15	1009.6858	1006.0081	1013.6337	1017.0107
303.15	1034.0765	1029.0024	1042.0084	1044.0296
308.15	1074.8919	1050.9923	1071.5053	1070.8549
313.15	1103.5024	1071.9785	1102.0960	1097.4799
		Methanol		
T/K	$10^5x_1^{exp}$	$10^5x_1^{Modified}$	$10^5x_1^{Yaws}$	$10^5x_1^{Van't\ Hoff}$

278.15	40.1229	40.8888	40.3474	42.2926
283.15	46.9901	46.1176	46.1286	46.7322
288.15	51.0849	51.4829	51.9339	51.4593
293.15	57.3764	56.9203	57.6544	56.4786
298.15	64.2412	62.3639	63.1885	61.7943
303.15	67.3861	67.7480	68.4446	67.4100
308.15	74.0998	73.0089	73.3445	73.3287
313.15	77.5686	78.0864	77.8238	79.5530

Ethanol

T/K	$10^5x_1^{exp}$	$10^5x_1^{Modified}$	$10^5x_1^{Yaws}$	$10^5x_1^{Van't\ Hoff}$
278.15	42.4873	42.5979	42.5910	43.0069
283.15	45.2223	44.7740	44.7790	44.8527
288.15	46.2857	46.8628	46.8701	46.7095
293.15	48.7680	48.8551	48.8586	48.5758
298.15	51.6970	50.7426	50.7402	50.4505
303.15	51.5709	52.5187	52.5124	52.3320
308.15	54.5434	54.1782	54.1737	54.2193
313.15	55.6763	55.7169	55.7239	56.1111

1-Propanol

T/K	$10^5x_1^{exp}$	$10^5x_1^{Modified}$	$10^5x_1^{Yaws}$	$10^5x_1^{Van't\ Hoff}$
278.15	9.7862	9.8881	9.7062	9.9726
283.15	10.8398	11.1826	11.1263	11.2167
288.15	12.7116	12.5725	12.6195	12.5647
293.15	14.2679	14.0570	14.1741	14.0202
298.15	16.2993	15.6348	15.7774	15.5869
303.15	16.7609	17.3037	17.4167	17.2683
308.15	19.1153	19.0611	19.0793	19.0676
313.15	20.8695	20.9040	20.7527	20.9878

2-Propanol

T/K	$10^5x_1^{exp}$	$10^5x_1^{Modified}$	$10^5x_1^{Yaws}$	$10^5x_1^{Van't\ Hoff}$
278.15	4.0588	3.9627	3.9131	4.1633
283.15	4.3491	4.4988	4.5152	4.5945
288.15	5.1603	5.0425	5.1121	5.0530
293.15	5.5835	5.5844	5.6888	5.5392
298.15	6.1346	6.1152	6.2316	6.0536
303.15	6.9668	6.6260	6.7287	6.5963
308.15	7.2122	7.1084	7.1706	7.1678
313.15	7.4492	7.5550	7.5502	7.7681

Acetone

https://doi.org/10.3390/pharmaceutics18060657

T/K	$10^5x_1^{exp}$	$10^5x_1^{Modified}$	$10^5x_1^{Yaws}$	$10^5x_1^{Van't\ Hoff}$
278.15	2.3135	2.4168	2.3257	2.4302
283.15	2.6764	2.6887	2.6322	2.6626
288.15	2.8578	2.9664	2.9359	2.9080
293.15	3.3114	3.2474	3.2311	3.1664
298.15	3.4475	3.5290	3.5130	3.4380
303.15	3.8557	3.8088	3.7771	3.7228
308.15	3.9465	4.0841	4.0197	4.0208
313.15	4.2640	4.3526	4.2381	4.3320

Acetonitrile

T/K	$10^5x_1^{exp}$	$10^5x_1^{Modified}$	$10^5x_1^{Yaws}$	$10^5x_1^{Van't\ Hoff}$
278.15	0.3817	0.3891	0.3895	0.4006
283.15	0.4353	0.4220	0.4231	0.4256
288.15	0.4509	0.4535	0.4550	0.4511
293.15	0.4888	0.4830	0.4847	0.4773
298.15	0.5135	0.5103	0.5119	0.5040
303.15	0.5225	0.5351	0.5364	0.5313
308.15	0.5656	0.5570	0.5582	0.5590
313.15	0.5775	0.5759	0.5770	0.5873

Hexane

T/K	$10^5x_1^{exp}$	$10^5x_1^{Modified}$	$10^5x_1^{Yaws}$	$10^5x_1^{Van't\ Hoff}$
278.15	0.2739	0.2765	0.2662	0.2705
283.15	0.3142	0.3153	0.3114	0.3131
288.15	0.3303	0.3594	0.3610	0.3605
293.15	0.4270	0.4095	0.4153	0.4130
298.15	0.4995	0.4663	0.4741	0.4711
303.15	0.5156	0.5308	0.5376	0.5350
308.15	0.6122	0.6039	0.6056	0.6051
313.15	0.6767	0.6867	0.6781	0.6817

Table S2. Correlation model-specific parameters for daptomycin solubility in selected solvents.

Model		Water	Methanol	Ethanol	1-Propanol	2-Propanol	Acetone	Acetoni-trile	Hexane
Apelbat	A	14.85 ± 27.21	131.29 ± 33.44	49.10± 37.91	32.41 ± 85.73	179.09 ± 42.89	94.98 ± 55.46	132.39 ± 34.07	-96.32 ± 116.28
	B	-1218.92 ± 1264.35	-7488.65 ± 2340.54	-3069.89 ± 1993.20	-3403.42 ± 4169.62	-9695.88 ± 3588.00	-5889.30 ± 3414.60	-7208.54 ± 2426.57	1778.35 ± 567.96
	C	-2.70 ± 4.04	-19.93 ± 5.07	-8.14 ± 5.63	-5.22 ± 2.81	-27.43 ± 6.52	-15.01 ± 8.29	-21.13 ± 5.07	13.70 ± 17.73
	R^2	0.9860	0.9945	0.9835	0.9912	0.9843	0.9876	0.9835	0.9821
	100RAD	1.0980	1.3673	0.8879	1.8174	2.0219	2.4762	1.4439	3.3304

https://doi.org/10.3390/pharmaceutics18060657

	10^5RMSD	15.1366	0.9238	0.5588	0.3413	0.1517	0.0883	0.0080	0.01823
Yaws	A_1	-0.6349 ± 1.5048	-18.04 ± 3.74	-9.44± 3.67	-11.25 ± 6.89	-25.26 ± 6.44	-20.79± 5.57	-20.01 ± 4.85	-9.20 ± 12.73
	B_1	-1871.73± 885.65	7883.70± 2232.77	1743.54 ± 2199.01	3318.70± 4118.58	10775.00 ± 3865.06	7666.97 ± 3353.43	5566.91 ± 2937.48	470.81 ± 7665.45
	C_1	206330.91 ± 133337.23	-1402149.48 ± 349114.97	-355363.56 ± 327792.42	-767448.36 ± 618882.32	-1828173.76 ± 619320.09	- 1349393.68± 523695.61	-964092.73 ± 451658.59	-412073.72 ± 1122360.24
	R^2	0.99478	0.9964	0.9834	0.9923	0.9881	0.9903	0.9842	0.9844
	100RAD	0.3627	1.1371	0.8890	1.5880	2.1452	1.7171	1.3707	3.2969
	10^5RMSD	4.5129	0.7462	0.5588	0.3207	0.1324	0.0624	0.0079	0.0170
Van′t Hoff	ΔG_{sol}^0 (J*mol^{-1})	11300.18 ± 5.57	18264.50 ± 25.82	18687.55 ± 12.88	21667.9967 ± 26.6200	23964.68 ± 37.85	25343.89 ± 29.32	30016.50 ± 22.65	30292.79 ± 44.17
	ΔH_{sol}^0 (J*mol^{-1})	3940.67 ± 142.26	13072.58 ± 644.46	5503.15 ± 326.08	15395.8587 ± 656.8817	12904.81 ± 939.62	11960.37 ± 728.87	7915.24± 565.52	19121.89 ± 1064.33
	R^2	0.9923	0.9868	0.9798	0.9902	0.9717	0.9798	0.9715	0.9840
	100RAD	0.4735	1.9621	1.0765	2.0610	2.8280	2.3616	1.9964	3.1667
	10^5RMSD	5.4813	1.4248	0.6175	0.3607	0.2037	0.0903	0.0105	0.0172

Table S3. The topology file for daptomycin.

[atomtypes]							
; name	at.num	mass	charge	ptype	sigma (nm)	epsilon (kJ/mol)	
ca	6	12.010736	0.000000	A	3.399670E-01	3.598240E-01	
na	7	14.006703	0.000000	A	3.249999E-01	7.112800E-01	
c2	6	12.010736	0.000000	A	3.399670E-01	3.598240E-01	
c3	6	12.010736	0.000000	A	3.399670E-01	4.577296E-01	
n	7	14.006703	0.000000	A	3.249999E-01	7.112800E-01	
c	6	12.010736	0.000000	A	3.399670E-01	3.598240E-01	
o	8	15.999405	0.000000	A	2.959922E-01	8.786400E-01	
os	8	15.999405	0.000000	A	3.000012E-01	7.112800E-01	
nh	7	14.006703	0.000000	A	3.249999E-01	7.112800E-01	
oh	8	15.999405	0.000000	A	3.066473E-01	8.803136E-01	
n3	7	14.006703	0.000000	A	3.249999E-01	7.112800E-01	
ha	1	1.007941	0.000000	A	2.599642E-01	6.276000E-02	
hn	1	1.007941	0.000000	A	1.069078E-01	6.568880E-02	
h4	1	1.007941	0.000000	A	2.510553E-01	6.276000E-02	
hc	1	1.007941	0.000000	A	2.649533E-01	6.568880E-02	
h1	1	1.007941	0.000000	A	2.471353E-01	6.568880E-02	
ho	1	1.007941	0.000000	A	0.000000E+00	0.000000E+00	
[moleculetype]							
; name	nrexcl						
dts	3						
[atoms]							
; Index	type	residue	resname	atom	cgnr	charge	mass
1	ca	1	MOL	C1	1	-0.1630	12.010736

2	ca	1	MOL	C2	2	-0.0850	12.010736
3	ca	1	MOL	C3	3	-0.0848	12.010736
4	ca	1	MOL	C4	4	-0.0562	12.010736
5	ca	1	MOL	C5	5	-0.1510	12.010736
6	ca	1	MOL	C6	6	-0.1180	12.010736
7	na	1	MOL	N7	7	-0.1944	14.006703
8	c2	1	MOL	C8	8	-0.0911	12.010736
9	c2	1	MOL	C9	9	-0.1594	12.010736
10	c3	1	MOL	C10	10	-0.013212.010736	
11	c3	1	MOL	C11	11	0.0537	12.010736
12	n	1	MOL	N12	12	-0.5739	14.006703
13	c	1	MOL	C13	13	0.6691	12.010736
14	o	1	MOL	O14	14	-0.6341	15.999405
15	c3	1	MOL	C15	15	-0.1464	12.010736
16	c3	1	MOL	C16	16	-0.0714	12.010736
17	c3	1	MOL	C17	17	-0.0784	12.010736
18	c3	1	MOL	C18	18	-0.0794	12.010736
19	c3	1	MOL	C19	19	-0.0784	12.010736
20	c3	1	MOL	C20	20	-0.0784	12.010736
21	c3	1	MOL	C21	21	-0.0774	12.010736
22	c3	1	MOL	C22	22	-0.0814	12.010736
23	c3	1	MOL	C23	23	-0.0921	12.010736
24	c	1	MOL	C24	24	0.6631	12.010736
25	n	1	MOL	N25	25	-0.5449	14.006703
26	c3	1	MOL	C26	26	0.0417	12.010736
27	c	1	MOL	C27	27	0.6631	12.010736
28	o	1	MOL	O28	28	-0.6371	15.999405
29	n	1	MOL	N29	29	-0.5589	14.006703
30	c3	1	MOL	C30	30	0.0607	12.010736
31	c	1	MOL	C31	31	0.6281	12.010736
32	n	1	MOL	N32	32	-0.5599	14.006703
33	c3	1	MOL	C33	33	0.0007	12.010736
34	c	1	MOL	C34	34	0.6291	12.010736
35	o	1	MOL	O35	35	-0.5851	15.999405
36	n	1	MOL	N36	36	-0.5359	14.006703
37	c3	1	MOL	C37	37	0.0180	12.010736
38	c	1	MOL	C38	38	0.6421	12.010736
39	n	1	MOL	N39	39	-0.5419	14.006703
40	c3	1	MOL	C40	40	0.0347	12.010736
41	c	1	MOL	C41	41	0.6521	12.010736
42	o	1	MOL	O42	42	-0.6441	15.999405
43	n	1	MOL	N43	43	-0.5389	14.006703

44	c3	1	MOL	C44	44	0.0527	12.010736
45	c	1	MOL	C45	45	0.6651	12.010736
46	n	1	MOL	N46	46	-0.5289	14.006703
47	c3	1	MOL	C47	47	0.0367	12.010736
48	c	1	MOL	C48	48	0.6231	12.010736
49	n	1	MOL	N49	49	-0.5499	14.006703
50	c3	1	MOL	C50	50	0.0627	12.010736
51	c	1	MOL	C51	51	0.6361	12.010736
52	n	1	MOL	N52	52	-0.5559	14.006703
53	c3	1	MOL	C53	53	0.0220	12.010736
54	c	1	MOL	C54	54	0.6481	12.010736
55	n	1	MOL	N55	55	-0.5389	14.006703
56	c3	1	MOL	C56	56	0.0007	12.010736
57	c	1	MOL	C57	57	0.6431	12.010736
58	n	1	MOL	N58	58	-0.5329	14.006703
59	c3	1	MOL	C59	59	0.0547	12.010736
60	c	1	MOL	C60	60	0.6601	12.010736
61	n	1	MOL	N61	61	-0.5669	14.006703
62	c3	1	MOL	C62	62	0.0857	12.010736
63	c	1	MOL	C63	63	0.6181	12.010736
64	os	1	MOL	O64	64	-0.4399	15.999405
65	c3	1	MOL	C65	65	0.1581	12.010736
66	c3	1	MOL	C66	66	-0.1161	12.010736
67	o	1	MOL	O67	67	-0.5460	15.999405
68	c3	1	MOL	C68	68	-0.1944	12.010736
69	c	1	MOL	C69	69	0.5987	12.010736
70	ca	1	MOL	C70	70	-0.2296	12.010736
71	ca	1	MOL	C71	71	0.1776	12.010736
72	ca	1	MOL	C72	72	-0.1860	12.010736
73	ca	1	MOL	C73	73	-0.0710	12.010736
74	ca	1	MOL	C74	74	-0.1700	12.010736
75	ca	1	MOL	C75	75	-0.0750	12.010736
76	nh	1	MOL	N76	76	-0.8272	14.006703
77	o	1	MOL	O77	77	-0.5211	15.999405
78	o	1	MOL	O78	78	-0.5871	15.999405
79	c3	1	MOL	C79	79	-0.0767	12.010736
80	c3	1	MOL	C80	80	-0.1171	12.010736
81	c3	1	MOL	C81	81	-0.1484	12.010736
82	c	1	MOL	C82	82	0.6401	12.010736
83	oh	1	MOL	O83	83	-0.6311	15.999405
84	o	1	MOL	O84	84	-0.5290	15.999405
85	o	1	MOL	O85	85	-0.5971	15.999405

86	c3	1	MOL	C86	86	0.0974	12.010736
87	oh	1	MOL	O87	87	-0.5898	15.999405
88	o	1	MOL	O88	88	-0.5871	15.999405
89	o	1	MOL	O89	89	-0.5761	15.999405
90	c3	1	MOL	C90	90	-0.2144	12.010736
91	c	1	MOL	C91	91	0.6591	12.010736
92	o	1	MOL	O92	92	-0.5430	15.999405
93	oh	1	MOL	O93	93	-0.5901	15.999405
94	o	1	MOL	O94	94	-0.5981	15.999405
95	c3	1	MOL	C95	95	-0.1041	12.010736
96	o	1	MOL	O96	96	-0.6201	15.999405
97	c3	1	MOL	C97	97	-0.2094	12.010736
98	c	1	MOL	C98	98	0.6441	12.010736
99	oh	1	MOL	O99	99	-0.5821	15.999405
100	o	1	MOL	O100	100	-0.5290	15.999405
101	c3	1	MOL	C101	101	-0.0834	12.010736
102	c3	1	MOL	C102	102	-0.1344	12.010736
103	c3	1	MOL	C103	103	0.1548	12.010736
104	n3	1	MOL	N104	104	-0.9058	14.006703
105	o	1	MOL	O105	105	-0.6761	15.999405
106	o	1	MOL	O106	106	-0.6331	15.999405
107	c3	1	MOL	C107	107	-0.1354	12.010736
108	c	1	MOL	C108	108	0.6421	12.010736
109	o	1	MOL	O109	109	-0.5940	15.999405
110	oh	1	MOL	O110	110	-0.5921	15.999405
111	c3	1	MOL	C111	111	-0.1674	12.010736
112	c	1	MOL	C112	112	0.6521	12.010736
113	n	1	MOL	N113	113	-0.6660	14.006703
114	o	1	MOL	O114	114	-0.6031	15.999405
115	o	1	MOL	O115	115	-0.6941	15.999405
116	ha	1	MOL	H116	116	0.1310	1.007941
117	ha	1	MOL	H117	117	0.1550	1.007941
118	ha	1	MOL	H118	118	0.1290	1.007941
119	ha	1	MOL	H119	119	0.1260	1.007941
120	hn	1	MOL	H120	120	0.2967	1.007941
121	h4	1	MOL	H121	121	0.1920	1.007941
122	hc	1	MOL	H122	122	0.0782	1.007941
123	hc	1	MOL	H123	123	0.0782	1.007941
124	h1	1	MOL	H124	124	0.1097	1.007941
125	hn	1	MOL	H125	125	0.3415	1.007941
126	hc	1	MOL	H126	126	0.0767	1.007941
127	hc	1	MOL	H127	127	0.0767	1.007941

128	hc	1	MOL	H128	128	0.0552	1.007941
129	hc	1	MOL	H129	129	0.0552	1.007941
130	hc	1	MOL	H130	130	0.0402	1.007941
131	hc	1	MOL	H131	131	0.0402	1.007941
132	hc	1	MOL	H132	132	0.0427	1.007941
133	hc	1	MOL	H133	133	0.0427	1.007941
134	hc	1	MOL	H134	134	0.0367	1.007941
135	hc	1	MOL	H135	135	0.0367	1.007941
136	hc	1	MOL	H136	136	0.0407	1.007941
137	hc	1	MOL	H137	137	0.0407	1.007941
138	hc	1	MOL	H138	138	0.0372	1.007941
139	hc	1	MOL	H139	139	0.0372	1.007941
140	hc	1	MOL	H140	140	0.0402	1.007941
141	hc	1	MOL	H141	141	0.0402	1.007941
142	hc	1	MOL	H142	142	0.0317	1.007941
143	hc	1	MOL	H143	143	0.0317	1.007941
144	hc	1	MOL	H144	144	0.0317	1.007941
145	hn	1	MOL	H145	145	0.3615	1.007941
146	h1	1	MOL	H146	146	0.1077	1.007941
147	hn	1	MOL	H147	147	0.3405	1.007941
148	h1	1	MOL	H148	148	0.1057 1.007941	
149	hn	1	MOL	H149	149	0.3535	1.007941
150	h1	1	MOL	H150	150	0.1217	1.007941
151	hn	1	MOL	H151	151	0.3275	1.007941
152	h1	1	MOL	H152	152	0.0927	1.007941
153	h1	1	MOL	H153	153	0.0927	1.007941
154	hn	1	MOL	H154	154	0.3555	1.007941
155	h1	1	MOL	H155	155	0.0827	1.007941
156	hn	1	MOL	H156	156	0.3545	1.007941
157	h1	1	MOL	H157	157	0.0887	1.007941
158	hn	1	MOL	H158	158	0.3265	1.007941
159	h1	1	MOL	H159	159	0.0987	1.007941
160	hn	1	MOL	H160	160	0.3525	1.007941
161	h1	1	MOL	H161	161	0.0727	1.007941
162	hn	1	MOL	H162	162	0.3455	1.007941
163	h1	1	MOL	H163	163	0.0712	1.007941
164	h1	1	MOL	H164	164	0.0712	1.007941
165	hn	1	MOL	H165	165	0.3335	1.007941
166	h1	1	MOL	H166	166	0.0877	1.007941
167	hn	1	MOL	H167	167	0.3245	1.007941
168	h1	1	MOL	H168	168	0.0847	1.007941
169	hn	1	MOL	H169	169	0.3265	1.007941

170	h1	1	MOL	H170	170	0.1087	1.007941
171	h1	1	MOL	H171	171	0.0877	1.007941
172	hc	1	MOL	H172	172	0.0667	1.007941
173	hc	1	MOL	H173	173	0.0667	1.007941
174	hc	1	MOL	H174	174	0.0667	1.007941
175	hc	1	MOL	H175	175	0.0907	1.007941
176	hc	1	MOL	H176	176	0.0907	1.007941
177	ha	1	MOL	H177	177	0.1430	1.007941
178	ha	1	MOL	H178	178	0.1410	1.007941
179	ha	1	MOL	H179	179	0.1420	1.007941
180	ha	1	MOL	H180	180	0.1310	1.007941
181	hn	1	MOL	H181	181	0.4003	1.007941
182	hn	1	MOL	H182	182	0.4003	1.007941
183	hc	1	MOL	H183	183	0.0767	1.007941
184	hc	1	MOL	H184	184	0.0587	1.007941
185	hc	1	MOL	H185	185	0.0587	1.007941
186	hc	1	MOL	H186	186	0.0587	1.007941
187	hc	1	MOL	H187	187	0.0982	1.007941
188	hc	1	MOL	H188	188	0.0982	1.007941
189	ho	1	MOL	H189	189	0.4540	1.007941
190	h1	1	MOL	H190	190	0.0642	1.007941
191	h1	1	MOL	H191	191	0.0642	1.007941
192	ho	1	MOL	H192	192	0.4280	1.007941
193	hc	1	MOL	H193	193	0.1077	1.007941
194	hc	1	MOL	H194	194	0.1077	1.007941
195	ho	1	MOL	H195	195	0.4610	1.007941
196	hc	1	MOL	H196	196	0.0534	1.007941
197	hc	1	MOL	H197	197	0.0534	1.007941
198	hc	1	MOL	H198	198	0.0534	1.007941
199	hc	1	MOL	H199	199	0.1167	1.007941
200	hc	1	MOL	H200	200	0.1167	1.007941
201	ho	1	MOL	H201	201	0.4600	1.007941
202	hc	1	MOL	H202	202	0.0652	1.007941
203	hc	1	MOL	H203	203	0.0652	1.007941
204	hc	1	MOL	H204	204	0.0612	1.007941
205	hc	1	MOL	H205	205	0.0612	1.007941
206	h1	1	MOL	H206	206	0.0457	1.007941
207	h1	1	MOL	H207	207	0.0457	1.007941
208	hn	1	MOL	H208	208	0.3503	1.007941
209	hn	1	MOL	H209	209	0.3503	1.007941
210	hc	1	MOL	H210	210	0.1092	1.007941
211	hc	1	MOL	H211	211	0.1092	1.007941

https://doi.org/10.3390/pharmaceutics18060657

212	ho	1	MOL	H212	212	0.4750	1.007941
213	hc	1	MOL	H213	213	0.0887	1.007941
214	hc	1	MOL	H214	214	0.0887	1.007941
215	hn	1	MOL	H215	215	0.3190	1.007941
216	hn	1	MOL	H216	216	0.3190	1.007941

[bonds]					
; atom_i	atom_j	functype	r0 (nm)	k (kJ/mol/nm^2)	
1	2	1	0.139840	3.858485E+05	; C1-C2, prebuilt ca-ca
1	6	1	0.139840	3.858485E+05	; C1-C6, prebuilt ca-ca
1	116	1	0.108600	2.893654E+05	; C1-H116, prebuilt ca-ha
2	3	1	0.139840	3.858485E+05	; C2-C3, prebuilt ca-ca
2	117	1	0.108600	2.893654E+05	; C2-H117, prebuilt ca-ha
3	4	1	0.139840	3.858485E+05	; C3-C4, prebuilt ca-ca
3	9	1	0.138460	4.034213E+05	; C3-C9, prebuilt c2-ca
4	5	1	0.139840	3.858485E+05	; C4-C5, prebuilt ca-ca
4	7	1	0.138400	3.518744E+05	; C4-N7, prebuilt ca-na
5	6	1	0.139840	3.858485E+05	; C5-C6, prebuilt ca-ca
5	118	1	0.108600	2.893654E+05	; C5-H118, prebuilt ca-ha
6	119	1	0.108600	2.893654E+05	; C6-H119, prebuilt ca-ha
7	8	1	0.140150	3.325443E+05	; N7-C8, prebuilt c2-na
7	120	1	0.101000	3.417491E+05	; N7-H120, prebuilt hn-na
8	9	1	0.133430	4.764739E+05	; C8-C9, prebuilt c2-c2
8	121	1	0.108680	2.883613E+05	; C8-H121, prebuilt c2-h4
9	10	1	0.150950	2.734662E+05	; C9-C10, prebuilt c2-c3
10	11	1	0.153750	2.517931E+05	; C10-C11, prebuilt c3-c3
10	122	1	0.109690	2.766461E+05	; C10-H122, prebuilt c3-hc
10	123	1	0.109690	2.766461E+05	; C10-H123, prebuilt c3-hc
11	12	1	0.146190	2.750562E+05	; C11-N12, prebuilt c3-n
11	24	1	0.152410	2.619184E+05	; C11-C24, prebuilt c-c3
11	124	1	0.109690	2.766461E+05	; C11-H124, prebuilt c3-h1
12	13	1	0.137890	3.578157E+05	; N12-C13, prebuilt c-n
12	125	1	0.101290	3.373978E+05	; N12-H125, prebuilt hn-n
13	14	1	0.121830	5.336274E+05	; C13-O14, prebuilt c-o
13	15	1	0.152410	2.619184E+05	; C13-C15, prebuilt c-c3
15	16	1	0.153750	2.517931E+05	; C15-C16, prebuilt c3-c3
15	126	1	0.109690	2.766461E+05	; C15-H126, prebuilt c3-hc
15	127	1	0.109690	2.766461E+05	; C15-H127, prebuilt c3-hc
16	17	1	0.153750	2.517931E+05	; C16-C17, prebuilt c3-c3
16	128	1	0.109690	2.766461E+05	; C16-H128, prebuilt c3-hc
16	129	1	0.109690	2.766461E+05	; C16-H129, prebuilt c3-hc
17	18	1	0.153750	2.517931E+05	; C17-C18, prebuilt c3-c3

17	130	1	0.109690	2.766461E+05	; C17-H130, prebuilt c3-hc
17	131	1	0.109690	2.766461E+05	; C17-H131, prebuilt c3-hc
18	19	1	0.153750	2.517931E+05	; C18-C19, prebuilt c3-c3
18	132	1	0.109690	2.766461E+05	; C18-H132, prebuilt c3-hc
18	133	1	0.109690	2.766461E+05	; C18-H133, prebuilt c3-hc
19	20	1	0.153750	2.517931E+05	; C19-C20, prebuilt c3-c3
19	134	1	0.109690	2.766461E+05	; C19-H134, prebuilt c3-hc
19	135	1	0.109690	2.766461E+05	; C19-H135, prebuilt c3-hc
20	21	1	0.153750	2.517931E+05	; C20-C21, prebuilt c3-c3
20	136	1	0.109690	2.766461E+05	; C20-H136, prebuilt c3-hc
20	137	1	0.109690	2.766461E+05	; C20-H137, prebuilt c3-hc
21	22	1	0.153750	2.517931E+05	; C21-C22, prebuilt c3-c3
21	138	1	0.109690	2.766461E+05	; C21-H138, prebuilt c3-hc
21	139	1	0.109690	2.766461E+05	; C21-H139, prebuilt c3-hc
22	23	1	0.153750	2.517931E+05	; C22-C23, prebuilt c3-c3
22	140	1	0.109690	2.766461E+05	; C22-H140, prebuilt c3-hc
22	141	1	0.109690	2.766461E+05	; C22-H141, prebuilt c3-hc
23	142	1	0.109690	2.766461E+05	; C23-H142, prebuilt c3-hc
23	143	1	0.109690	2.766461E+05	; C23-H143, prebuilt c3-hc
23	144	1	0.109690	2.766461E+05	; C23-H144, prebuilt c3-hc
24	25	1	0.137890	3.578157E+05	; C24-N25, prebuilt c-n
24	115	1	0.121830	5.336274E+05	; C24-O115, prebuilt c-o
25	26	1	0.146190	2.750562E+05	; N25-C26, prebuilt c3-n
25	145	1	0.101290	3.373978E+05	; N25-H145, prebuilt hn-n
26	27	1	0.152410	2.619184E+05	; C26-C27, prebuilt c-c3
26	111	1	0.153750	2.517931E+05	; C26-C111, prebuilt c3-c3
26	146	1	0.109690	2.766461E+05	; C26-H146, prebuilt c3-h1
27	28	1	0.121830	5.336274E+05	; C27-O28, prebuilt c-o
27	29	1	0.137890	3.578157E+05	; C27-N29, prebuilt c-n
29	30	1	0.146190	2.750562E+05	; N29-C30, prebuilt c3-n
29	147	1	0.101290	3.373978E+05	; N29-H147, prebuilt hn-n
30	31	1	0.152410	2.619184E+05	; C30-C31, prebuilt c-c3
30	107	1	0.153750	2.517931E+05	; C30-C107, prebuilt c3-c3
30	148	1	0.109690	2.766461E+05	; C30-H148, prebuilt c3-h1
31	32	1	0.137890	3.578157E+05	; C31-N32, prebuilt c-n
31	106	1	0.121830	5.336274E+05	; C31-O106, prebuilt c-o
32	33	1	0.146190	2.750562E+05	; N32-C33, prebuilt c3-n
32	149	1	0.101290	3.373978E+05	; N32-H149, prebuilt hn-n
33	34	1	0.152410	2.619184E+05	; C33-C34, prebuilt c-c3
33	65	1	0.153750	2.517931E+05	; C33-C65, prebuilt c3-c3
33	150	1	0.109690	2.766461E+05	; C33-H150, prebuilt c3-h1
34	35	1	0.121830	5.336274E+05	; C34-O35, prebuilt c-o

34	36	1	0.137890	3.578157E+05	; C34-N36, prebuilt c-n
36	37	1	0.146190	2.750562E+05	; N36-C37, prebuilt c3-n
36	151	1	0.101290	3.373978E+05	; N36-H151, prebuilt hn-n
37	38	1	0.152410	2.619184E+05	; C37-C38, prebuilt c-c3
37	152	1	0.109690	2.766461E+05	; C37-H152, prebuilt c3-h1
37	153	1	0.109690	2.766461E+05	; C37-H153, prebuilt c3-h1
38	39	1	0.137890	3.578157E+05	; C38-N39, prebuilt c-n
38	105	1	0.121830	5.336274E+05	; C38-O105, prebuilt c-o
39	40	1	0.146190	2.750562E+05	; N39-C40, prebuilt c3-n
39	154	1	0.101290	3.373978E+05	; N39-H154, prebuilt hn-n
40	41	1	0.152410	2.619184E+05	; C40-C41, prebuilt c-c3
40	101	1	0.153750	2.517931E+05	; C40-C101, prebuilt c3-c3
40	155	1	0.109690	2.766461E+05	; C40-H155, prebuilt c3-h1
41	42	1	0.121830	5.336274E+05	; C41-O42, prebuilt c-o
41	43	1	0.137890	3.578157E+05	; C41-N43, prebuilt c-n
43	44	1	0.146190	2.750562E+05	; N43-C44, prebuilt c3-n
43	156	1	0.101290	3.373978E+05	; N43-H156, prebuilt hn-n
44	45	1	0.152410	2.619184E+05	; C44-C45, prebuilt c-c3
44	97	1	0.153750	2.517931E+05	; C44-C97, prebuilt c3-c3
44	157	1	0.109690	2.766461E+05	; C44-H157, prebuilt c3-h1
45	46	1	0.137890	3.578157E+05	; C45-N46, prebuilt c-n
45	96	1	0.121830	5.336274E+05	; C45-O96, prebuilt c-o
46	47	1	0.146190	2.750562E+05	; N46-C47, prebuilt c3-n
46	158	1	0.101290	3.373978E+05	; N46-H158, prebuilt hn-n
47	48	1	0.152410	2.619184E+05	; C47-C48, prebuilt c-c3
47	95	1	0.153750	2.517931E+05	; C47-C95, prebuilt c3-c3
47	159	1	0.109690	2.766461E+05	; C47-H159, prebuilt c3-h1
48	49	1	0.137890	3.578157E+05	; C48-N49, prebuilt c-n
48	94	1	0.121830	5.336274E+05	; C48-O94, prebuilt c-o
49	50	1	0.146190	2.750562E+05	; N49-C50, prebuilt c3-n
49	160	1	0.101290	3.373978E+05	; N49-H160, prebuilt hn-n
50	51	1	0.152410	2.619184E+05	; C50-C51, prebuilt c-c3
50	90	1	0.153750	2.517931E+05	; C50-C90, prebuilt c3-c3
50	161	1	0.109690	2.766461E+05	; C50-H161, prebuilt c3-h1
51	52	1	0.137890	3.578157E+05	; C51-N52, prebuilt c-n
51	89	1	0.121830	5.336274E+05	; C51-O89, prebuilt c-o
52	53	1	0.146190	2.750562E+05	; N52-C53, prebuilt c3-n
52	162	1	0.101290	3.373978E+05	; N52-H162, prebuilt hn-n
53	54	1	0.152410	2.619184E+05	; C53-C54, prebuilt c-c3
53	163	1	0.109690	2.766461E+05	; C53-H163, prebuilt c3-h1
53	164	1	0.109690	2.766461E+05	; C53-H164, prebuilt c3-h1
54	55	1	0.137890	3.578157E+05	; C54-N55, prebuilt c-n

https://doi.org/10.3390/pharmaceutics18060657

54	88	1	0.121830	5.336274E+05	; C54-O88, prebuilt c-o
55	56	1	0.146190	2.750562E+05	; N55-C56, prebuilt c3-n
55	165	1	0.101290	3.373978E+05	; N55-H165, prebuilt hn-n
56	57	1	0.152410	2.619184E+05	; C56-C57, prebuilt c-c3
56	86	1	0.153750	2.517931E+05	; C56-C86, prebuilt c3-c3
56	166	1	0.109690	2.766461E+05	; C56-H166, prebuilt c3-h1
57	58	1	0.137890	3.578157E+05	; C57-N58, prebuilt c-n
57	85	1	0.121830	5.336274E+05	; C57-O85, prebuilt c-o
58	59	1	0.146190	2.750562E+05	; N58-C59, prebuilt c3-n
58	167	1	0.101290	3.373978E+05	; N58-H167, prebuilt hn-n
59	60	1	0.152410	2.619184E+05	; C59-C60, prebuilt c-c3
59	79	1	0.153750	2.517931E+05	; C59-C79, prebuilt c3-c3
59	168	1	0.109690	2.766461E+05	; C59-H168, prebuilt c3-h1
60	61	1	0.137890	3.578157E+05	; C60-N61, prebuilt c-n
60	78	1	0.121830	5.336274E+05	; C60-O78, prebuilt c-o
61	62	1	0.146190	2.750562E+05	; N61-C62, prebuilt c3-n
61	169	1	0.101290	3.373978E+05	; N61-H169, prebuilt hn-n
62	63	1	0.152410	2.619184E+05	; C62-C63, prebuilt c-c3
62	68	1	0.153750	2.517931E+05	; C62-C68, prebuilt c3-c3
62	170	1	0.109690	2.766461E+05	; C62-H170, prebuilt c3-h1
63	64	1	0.135840	3.270214E+05	; C63-O64, prebuilt c-os
63	67	1	0.121830	5.336274E+05	; C63-O67, prebuilt c-o
64	65	1	0.143160	2.582365E+05	; O64-C65, prebuilt c3-os
65	66	1	0.153750	2.517931E+05	; C65-C66, prebuilt c3-c3
65	171	1	0.109690	2.766461E+05	; C65-H171, prebuilt c3-h1
66	172	1	0.109690	2.766461E+05	; C66-H172, prebuilt c3-hc
66	173	1	0.109690	2.766461E+05	; C66-H173, prebuilt c3-hc
66	174	1	0.109690	2.766461E+05	; C66-H174, prebuilt c3-hc
68	69	1	0.152410	2.619184E+05	; C68-C69, prebuilt c-c3
68	175	1	0.109690	2.766461E+05	; C68-H175, prebuilt c3-hc
68	176	1	0.109690	2.766461E+05	; C68-H176, prebuilt c3-hc
69	70	1	0.149060	2.894491E+05	; C69-C70, prebuilt c-ca
69	77	1	0.121830	5.336274E+05	; C69-O77, prebuilt c-o
70	71	1	0.139840	3.858485E+05	; C70-C71, prebuilt ca-ca
70	75	1	0.139840	3.858485E+05	; C70-C75, prebuilt ca-ca
71	72	1	0.139840	3.858485E+05	; C71-C72, prebuilt ca-ca
71	76	1	0.138590	3.496987E+05	; C71-N76, prebuilt ca-nh
72	73	1	0.139840	3.858485E+05	; C72-C73, prebuilt ca-ca
72	177	1	0.108600	2.893654E+05	; C72-H177, prebuilt ca-ha
73	74	1	0.139840	3.858485E+05	; C73-C74, prebuilt ca-ca
73	178	1	0.108600	2.893654E+05	; C73-H178, prebuilt ca-ha
74	75	1	0.139840	3.858485E+05	; C74-C75, prebuilt ca-ca

https://doi.org/10.3390/pharmaceutics18060657

74	179	1	0.108600	2.893654E+05	; C74-H179, prebuilt ca-ha
75	180	1	0.108600	2.893654E+05	; C75-H180, prebuilt ca-ha
76	181	1	0.101210	3.385693E+05	; N76-H181, prebuilt hn-nh
76	182	1	0.101210	3.385693E+05	; N76-H182, prebuilt hn-nh
79	80	1	0.153750	2.517931E+05	; C79-C80, prebuilt c3-c3
79	81	1	0.153750	2.517931E+05	; C79-C81, prebuilt c3-c3
79	183	1	0.109690	2.766461E+05	; C79-H183, prebuilt c3-hc
80	184	1	0.109690	2.766461E+05	; C80-H184, prebuilt c3-hc
80	185	1	0.109690	2.766461E+05	; C80-H185, prebuilt c3-hc
80	186	1	0.109690	2.766461E+05	; C80-H186, prebuilt c3-hc
81	82	1	0.152410	2.619184E+05	; C81-C82, prebuilt c-c3
81	187	1	0.109690	2.766461E+05	; C81-H187, prebuilt c3-hc
81	188	1	0.109690	2.766461E+05	; C81-H188, prebuilt c3-hc
82	83	1	0.135130	3.348037E+05	; C82-O83, prebuilt c-oh
82	84	1	0.121830	5.336274E+05	; C82-O84, prebuilt c-o
83	189	1	0.097300	3.107875E+05	; O83-H189, prebuilt ho-oh
86	87	1	0.142330	2.650146E+05	; C86-O87, prebuilt c3-oh
86	190	1	0.109690	2.766461E+05	; C86-H190, prebuilt c3-h1
86	191	1	0.109690	2.766461E+05	; C86-H191, prebuilt c3-h1
87	192	1	0.097300	3.107875E+05	; O87-H192, prebuilt ho-oh
90	91	1	0.152410	2.619184E+05	; C90-C91, prebuilt c-c3
90	193	1	0.109690	2.766461E+05	; C90-H193, prebuilt c3-hc
90	194	1	0.109690	2.766461E+05	; C90-H194, prebuilt c3-hc
91	92	1	0.121830	5.336274E+05	; C91-O92, prebuilt c-o
91	93	1	0.135130	3.348037E+05	; C91-O93, prebuilt c-oh
93	195	1	0.097300	3.107875E+05	; O93-H195, prebuilt ho-oh
95	196	1	0.109690	2.766461E+05	; C95-H196, prebuilt c3-hc
95	197	1	0.109690	2.766461E+05	; C95-H197, prebuilt c3-hc
95	198	1	0.109690	2.766461E+05	; C95-H198, prebuilt c3-hc
97	98	1	0.152410	2.619184E+05	; C97-C98, prebuilt c-c3
97	199	1	0.109690	2.766461E+05	; C97-H199, prebuilt c3-hc
97	200	1	0.109690	2.766461E+05	; C97-H200, prebuilt c3-hc
98	99	1	0.135130	3.348037E+05	; C98-O99, prebuilt c-oh
98	100	1	0.121830	5.336274E+05	; C98-O100, prebuilt c-o
99	201	1	0.097300	3.107875E+05	; O99-H201, prebuilt ho-oh
101	102	1	0.153750	2.517931E+05	; C101-C102, prebuilt c3-c3
101	202	1	0.109690	2.766461E+05	; C101-H202, prebuilt c3-hc
101	203	1	0.109690	2.766461E+05	; C101-H203, prebuilt c3-hc
102	103	1	0.153750	2.517931E+05	; C102-C103, prebuilt c3-c3
102	204	1	0.109690	2.766461E+05	; C102-H204, prebuilt c3-hc
102	205	1	0.109690	2.766461E+05	; C102-H205, prebuilt c3-hc
103	104	1	0.146470	2.727131E+05	; C103-N104, prebuilt c3-n3

103	206	1	0.109690	2.766461E+05	; C103-H206, prebuilt c3-h1
103	207	1	0.109690	2.766461E+05	; C103-H207, prebuilt c3-h1
104	208	1	0.101900	3.283603E+05	; N104-H208, prebuilt hn-n3
104	209	1	0.101900	3.283603E+05	; N104-H209, prebuilt hn-n3
107	108	1	0.152410	2.619184E+05	; C107-C108, prebuilt c-c3
107	210	1	0.109690	2.766461E+05	; C107-H210, prebuilt c3-hc
107	211	1	0.109690	2.766461E+05	; C107-H211, prebuilt c3-hc
108	109	1	0.121830	5.336274E+05	; C108-O109, prebuilt c-o
108	110	1	0.135130	3.348037E+05	; C108-O110, prebuilt c-oh
110	212	1	0.097300	3.107875E+05	; O110-H212, prebuilt ho-oh
111	112	1	0.152410	2.619184E+05	; C111-C112, prebuilt c-c3
111	213	1	0.109690	2.766461E+05	; C111-H213, prebuilt c3-hc
111	214	1	0.109690	2.766461E+05	; C111-H214, prebuilt c3-hc
112	113	1	0.137890	3.578157E+05	; C112-N113, prebuilt c-n
112	114	1	0.121830	5.336274E+05	; C112-O114, prebuilt c-o
113	215	1	0.101290	3.373978E+05	; N113-H215, prebuilt hn-n
113	216	1	0.101290	3.373978E+05	; N113-H216, prebuilt hn-n

[angles]						
; atom_i	atom_j	atom_k	functype	a0 (Deg.)	k (kJ/mol/rad^2)	
2	1	6	1	120.020	5.573088E+02	; C2-C1-C6, prebuilt ca-ca-ca
2	1	116	1	119.880	4.033376E+02	; C2-C1-H116, prebuilt ca-ca-ha
6	1	116	1	119.880	4.033376E+02	; C6-C1-H116, prebuilt ca-ca-ha
1	2	3	1	120.020	5.573088E+02	; C1-C2-C3, prebuilt ca-ca-ca
1	2	117	1	119.880	4.033376E+02	; C1-C2-H117, prebuilt ca-ca-ha
3	2	117	1	119.880	4.033376E+02	; C3-C2-H117, prebuilt ca-ca-ha
2	3	4	1	120.020	5.573088E+02	; C2-C3-C4, prebuilt ca-ca-ca
2	3	9	1	120.600	5.589824E+02	; C2-C3-C9, prebuilt c2-ca-ca
4	3	9	1	120.600	5.589824E+02	; C4-C3-C9, prebuilt c2-ca-ca
3	4	5	1	120.020	5.573088E+02	; C3-C4-C5, prebuilt ca-ca-ca
3	4	7	1	118.340	5.782288E+02	; C3-C4-N7, prebuilt ca-ca-na
5	4	7	1	118.340	5.782288E+02	; C5-C4-N7, prebuilt ca-ca-na
4	5	6	1	120.020	5.573088E+02	; C4-C5-C6, prebuilt ca-ca-ca
4	5	118	1	119.880	4.033376E+02	; C4-C5-H118, prebuilt ca-ca-ha
6	5	118	1	119.880	4.033376E+02	; C6-C5-H118, prebuilt ca-ca-ha
1	6	5	1	120.020	5.573088E+02	; C1-C6-C5, prebuilt ca-ca-ca

1	6	119	1	119.880	4.033376E+02	; C1-C6-H119, prebuilt ca-ca-ha
5	6	119	1	119.880	4.033376E+02	; C5-C6-H119, prebuilt ca-ca-ha
4	7	8	1	124.970	5.322048E+02	; C4-N7-C8, prebuilt c2-na-ca
4	7	120	1	125.540	3.899488E+02	; C4-N7-H120, prebuilt ca-na-hn
8	7	120	1	119.280	3.958064E+02	; C8-N7-H120, prebuilt c2-na-hn
7	8	9	1	121.940	5.782288E+02	; N7-C8-C9, prebuilt c2-c2-na
7	8	121	1	112.970	4.250944E+02	; N7-C8-H121, prebuilt h4-c2-na
9	8	121	1	122.670	4.133792E+02	; C9-C8-H121, prebuilt c2-c2-h4
3	9	8	1	117.000	5.807392E+02	; C3-C9-C8, prebuilt c2-c2-ca
3	9	10	1	126.161	5.000000E+02	; C3-C9-C10 (ca-c2-c3) missing and simply guessed
8	9	10	1	123.630	5.363888E+02	; C8-C9-C10, prebuilt c2-c2-c3
9	10	11	1	111.560	5.305312E+02	; C9-C10-C11, prebuilt c2-c3-c3
9	10	122	1	110.360	3.932960E+02	; C9-C10-H122, prebuilt c2-c3-hc
9	10	123	1	110.360	3.932960E+02	; C9-C10-H123, prebuilt c2-c3-hc
11	10	122	1	109.800	3.874384E+02	; C11-C10-H122, prebuilt c3-c3-hc
11	10	123	1	109.800	3.874384E+02	; C11-C10-H123, prebuilt c3-c3-hc
122	10	123	1	107.580	3.296992E+02	; H122-C10-H123, prebuilt hc-c3-hc
10	11	12	1	111.610	5.514512E+02	; C10-C11-N12, prebuilt c3-c3-n
10	11	24	1	111.040	5.296944E+02	; C10-C11-C24, prebuilt c-c3-c3
10	11	124	1	109.560	3.882752E+02	; C10-C11-H124, prebuilt c3-c3-h1
12	11	24	1	109.060	5.606560E+02	; N12-C11-C24, prebuilt c-c3-n
12	11	124	1	108.880	4.167264E+02	; N12-C11-H124, prebuilt h1-c3-n
24	11	124	1	108.220	3.932960E+02	; C24-C11-H124, prebuilt c-c3-h1

11	12	13	1	120.690	5.305312E+02	; C11-N12-C13, prebuilt c-n-c3
11	12	125	1	117.680	3.832544E+02	; C11-N12-H125, prebuilt c3-n-hn
13	12	125	1	117.550	4.041744E+02	; C13-N12-H125, prebuilt c-n-hn
12	13	14	1	123.050	6.209056E+02	; N12-C13-O14, prebuilt n-c-o
12	13	15	1	115.180	5.589824E+02	; N12-C13-C15, prebuilt c3-c-n
14	13	15	1	123.200	5.640032E+02	; O14-C13-C15, prebuilt c3-c-o
13	15	16	1	111.040	5.296944E+02	; C13-C15-C16, prebuilt c-c3-c3
13	15	126	1	108.770	3.924592E+02	; C13-C15-H126, prebuilt c-c3-hc
13	15	127	1	108.770	3.924592E+02	; C13-C15-H127, prebuilt c-c3-hc
16	15	126	1	109.800	3.874384E+02	; C16-C15-H126, prebuilt c3-c3-hc
16	15	127	1	109.800	3.874384E+02	; C16-C15-H127, prebuilt c3-c3-hc
126	15	127	1	107.580	3.296992E+02	; H126-C15-H127, prebuilt hc-c3-hc
15	16	17	1	111.510	5.263472E+02	; C15-C16-C17, prebuilt c3-c3-c3
15	16	128	1	109.800	3.874384E+02	; C15-C16-H128, prebuilt c3-c3-hc
15	16	129	1	109.800	3.874384E+02	; C15-C16-H129, prebuilt c3-c3-hc
17	16	128	1	109.800	3.874384E+02	; C17-C16-H128, prebuilt c3-c3-hc
17	16	129	1	109.800	3.874384E+02	; C17-C16-H129, prebuilt c3-c3-hc
128	16	129	1	107.580	3.296992E+02	; H128-C16-H129, prebuilt hc-c3-hc
16	17	18	1	111.510	5.263472E+02	; C16-C17-C18, prebuilt c3-c3-c3
16	17	130	1	109.800	3.874384E+02	; C16-C17-H130, prebuilt c3-c3-hc
16	17	131	1	109.800	3.874384E+02	; C16-C17-H131, prebuilt c3-c3-hc

18	17	130	1	109.800	3.874384E+02	; C18-C17-H130, prebuilt c3-c3-hc
18	17	131	1	109.800	3.874384E+02	; C18-C17-H131, prebuilt c3-c3-hc
130	17	131	1	107.580	3.296992E+02	; H130-C17-H131, prebuilt hc-c3-hc
17	18	19	1	111.510	5.263472E+02	; C17-C18-C19, prebuilt c3-c3-c3
17	18	132	1	109.800	3.874384E+02	; C17-C18-H132, prebuilt c3-c3-hc
17	18	133	1	109.800	3.874384E+02	; C17-C18-H133, prebuilt c3-c3-hc
19	18	132	1	109.800	3.874384E+02	; C19-C18-H132, prebuilt c3-c3-hc
19	18	133	1	109.800	3.874384E+02	; C19-C18-H133, prebuilt c3-c3-hc
132	18	133	1	107.580	3.296992E+02	; H132-C18-H133, prebuilt hc-c3-hc
18	19	20	1	111.510	5.263472E+02	; C18-C19-C20, prebuilt c3-c3-c3
18	19	134	1	109.800	3.874384E+02	; C18-C19-H134, prebuilt c3-c3-hc
18	19	135	1	109.800	3.874384E+02	; C18-C19-H135, prebuilt c3-c3-hc
20	19	134	1	109.800	3.874384E+02	; C20-C19-H134, prebuilt c3-c3-hc
20	19	135	1	109.800	3.874384E+02	; C20-C19-H135, prebuilt c3-c3-hc
134	19	135	1	107.580	3.296992E+02	; H134-C19-H135, prebuilt hc-c3-hc
19	20	21	1	111.510	5.263472E+02	; C19-C20-C21, prebuilt c3-c3-c3
19	20	136	1	109.800	3.874384E+02	; C19-C20-H136, prebuilt c3-c3-hc
19	20	137	1	109.800	3.874384E+02	; C19-C20-H137, prebuilt c3-c3-hc
21	20	136	1	109.800	3.874384E+02	; C21-C20-H136, prebuilt c3-c3-hc
21	20	137	1	109.800	3.874384E+02	; C21-C20-H137, prebuilt c3-c3-hc
136	20	137	1	107.580	3.296992E+02	; H136-C20-H137, prebuilt hc-c3-hc

20	21	22	1	111.510	5.263472E+02	; C20-C21-C22, prebuilt c3-c3-c3
20	21	138	1	109.800	3.874384E+02	; C20-C21-H138, prebuilt c3-c3-hc
20	21	139	1	109.800	3.874384E+02	; C20-C21-H139, prebuilt c3-c3-hc
22	21	138	1	109.800	3.874384E+02	; C22-C21-H138, prebuilt c3-c3-hc
22	21	139	1	109.800	3.874384E+02	; C22-C21-H139, prebuilt c3-c3-hc
138	21	139	1	107.580	3.296992E+02	; H138-C21-H139, prebuilt hc-c3-hc
21	22	23	1	111.510	5.263472E+02	; C21-C22-C23, prebuilt c3-c3-c3
21	22	140	1	109.800	3.874384E+02	; C21-C22-H140, prebuilt c3-c3-hc
21	22	141	1	109.800	3.874384E+02	; C21-C22-H141, prebuilt c3-c3-hc
23	22	140	1	109.800	3.874384E+02	; C23-C22-H140, prebuilt c3-c3-hc
23	22	141	1	109.800	3.874384E+02	; C23-C22-H141, prebuilt c3-c3-hc
140	22	141	1	107.580	3.296992E+02	; H140-C22-H141, prebuilt hc-c3-hc
22	23	142	1	109.800	3.874384E+02	; C22-C23-H142, prebuilt c3-c3-hc
22	23	143	1	109.800	3.874384E+02	; C22-C23-H143, prebuilt c3-c3-hc
22	23	144	1	109.800	3.874384E+02	; C22-C23-H144, prebuilt c3-c3-hc
142	23	143	1	107.580	3.296992E+02	; H142-C23-H143, prebuilt hc-c3-hc
142	23	144	1	107.580	3.296992E+02	; H142-C23-H144, prebuilt hc-c3-hc
143	23	144	1	107.580	3.296992E+02	; H143-C23-H144, prebuilt hc-c3-hc
11	24	25	1	115.180	5.589824E+02	; C11-C24-N25, prebuilt c3-c-n
11	24	115	1	123.200	5.640032E+02	; C11-C24-O115, prebuilt c3-c-o
25	24	115	1	123.050	6.209056E+02	; N25-C24-O115, prebuilt n-c-o

24	25	26	1	120.690	5.305312E+02	; C24-N25-C26, prebuilt c-n-c3
24	25	145	1	117.550	4.041744E+02	; C24-N25-H145, prebuilt c-n-hn
26	25	145	1	117.680	3.832544E+02	; C26-N25-H145, prebuilt c3-n-hn
25	26	27	1	109.060	5.606560E+02	; N25-C26-C27, prebuilt c-c3-n
25	26	111	1	111.610	5.514512E+02	; N25-C26-C111, prebuilt c3-c3-n
25	26	146	1	108.880	4.167264E+02	; N25-C26-H146, prebuilt h1-c3-n
27	26	111	1	111.040	5.296944E+02	; C27-C26-C111, prebuilt c-c3-c3
27	26	146	1	108.220	3.932960E+02	; C27-C26-H146, prebuilt c-c3-h1
111	26	146	1	109.560	3.882752E+02	; C111-C26-H146, prebuilt c3-c3-h1
26	27	28	1	123.200	5.640032E+02	; C26-C27-O28, prebuilt c3-c-o
26	27	29	1	115.180	5.589824E+02	; C26-C27-N29, prebuilt c3-c-n
28	27	29	1	123.050	6.209056E+02	; O28-C27-N29, prebuilt n-c-o
27	29	30	1	120.690	5.305312E+02	; C27-N29-C30, prebuilt c-n-c3
27	29	147	1	117.550	4.041744E+02	; C27-N29-H147, prebuilt c-n-hn
30	29	147	1	117.680	3.832544E+02	; C30-N29-H147, prebuilt c3-n-hn
29	30	31	1	109.060	5.606560E+02	; N29-C30-C31, prebuilt c-c3-n
29	30	107	1	111.610	5.514512E+02	; N29-C30-C107, prebuilt c3-c3-n
29	30	148	1	108.880	4.167264E+02	; N29-C30-H148, prebuilt h1-c3-n
31	30	107	1	111.040	5.296944E+02	; C31-C30-C107, prebuilt c-c3-c3
31	30	148	1	108.220	3.932960E+02	; C31-C30-H148, prebuilt c-c3-h1
107	30	148	1	109.560	3.882752E+02	; C107-C30-H148, prebuilt c3-c3-h1

30	31	32	1	115.180	5.589824E+02	; C30-C31-N32, prebuilt c3-c-n
30	31	106	1	123.200	5.640032E+02	; C30-C31-O106, prebuilt c3-c-o
32	31	106	1	123.050	6.209056E+02	; N32-C31-O106, prebuilt n-c-o
31	32	33	1	120.690	5.305312E+02	; C31-N32-C33, prebuilt c-n-c3
31	32	149	1	117.550	4.041744E+02	; C31-N32-H149, prebuilt c-n-hn
33	32	149	1	117.680	3.832544E+02	; C33-N32-H149, prebuilt c3-n-hn
32	33	34	1	109.060	5.606560E+02	; N32-C33-C34, prebuilt c-c3-n
32	33	65	1	111.610	5.514512E+02	; N32-C33-C65, prebuilt c3-c3-n
32	33	150	1	108.880	4.167264E+02	; N32-C33-H150, prebuilt h1-c3-n
34	33	65	1	111.040	5.296944E+02	; C34-C33-C65, prebuilt c-c3-c3
34	33	150	1	108.220	3.932960E+02	; C34-C33-H150, prebuilt c-c3-h1
65	33	150	1	109.560	3.882752E+02	; C65-C33-H150, prebuilt c3-c3-h1
33	34	35	1	123.200	5.640032E+02	; C33-C34-O35, prebuilt c3-c-o
33	34	36	1	115.180	5.589824E+02	; C33-C34-N36, prebuilt c3-c-n
35	34	36	1	123.050	6.209056E+02	; O35-C34-N36, prebuilt n-c-o
34	36	37	1	120.690	5.305312E+02	; C34-N36-C37, prebuilt c-n-c3
34	36	151	1	117.550	4.041744E+02	; C34-N36-H151, prebuilt c-n-hn
37	36	151	1	117.680	3.832544E+02	; C37-N36-H151, prebuilt c3-n-hn
36	37	38	1	109.060	5.606560E+02	; N36-C37-C38, prebuilt c-c3-n
36	37	152	1	108.880	4.167264E+02	; N36-C37-H152, prebuilt h1-c3-n
36	37	153	1	108.880	4.167264E+02	; N36-C37-H153, prebuilt h1-c3-n

https://doi.org/10.3390/pharmaceutics18060657

38	37	152	1	108.220	3.932960E+02	; C38-C37-H152, prebuilt c-c3-h1
38	37	153	1	108.220	3.932960E+02	; C38-C37-H153, prebuilt c-c3-h1
152	37	153	1	108.460	3.280256E+02	; H152-C37-H153, prebuilt h1-c3-h1
37	38	39	1	115.180	5.589824E+02	; C37-C38-N39, prebuilt c3-c-n
37	38	105	1	123.200	5.640032E+02	; C37-C38-O105, prebuilt c3-c-o
39	38	105	1	123.050	6.209056E+02	; N39-C38-O105, prebuilt n-c-o
38	39	40	1	120.690	5.305312E+02	; C38-N39-C40, prebuilt c-n-c3
38	39	154	1	117.550	4.041744E+02	; C38-N39-H154, prebuilt c-n-hn
40	39	154	1	117.680	3.832544E+02	; C40-N39-H154, prebuilt c3-n-hn
39	40	41	1	109.060	5.606560E+02	; N39-C40-C41, prebuilt c-c3-n
39	40	101	1	111.610	5.514512E+02	; N39-C40-C101, prebuilt c3-c3-n
39	40	155	1	108.880	4.167264E+02	; N39-C40-H155, prebuilt h1-c3-n
41	40	101	1	111.040	5.296944E+02	; C41-C40-C101, prebuilt c-c3-c3
41	40	155	1	108.220	3.932960E+02	; C41-C40-H155, prebuilt c-c3-h1
101	40	155	1	109.560	3.882752E+02	; C101-C40-H155, prebuilt c3-c3-h1
40	41	42	1	123.200	5.640032E+02	; C40-C41-O42, prebuilt c3-c-o
40	41	43	1	115.180	5.589824E+02	; C40-C41-N43, prebuilt c3-c-n
42	41	43	1	123.050	6.209056E+02	; O42-C41-N43, prebuilt n-c-o
41	43	44	1	120.690	5.305312E+02	; C41-N43-C44, prebuilt c-n-c3
41	43	156	1	117.550	4.041744E+02	; C41-N43-H156, prebuilt c-n-hn
44	43	156	1	117.680	3.832544E+02	; C44-N43-H156, prebuilt c3-n-hn

43	44	45	1	109.060	5.606560E+02	; N43-C44-C45, prebuilt c-c3-n
43	44	97	1	111.610	5.514512E+02	; N43-C44-C97, prebuilt c3-c3-n
43	44	157	1	108.880	4.167264E+02	; N43-C44-H157, prebuilt h1-c3-n
45	44	97	1	111.040	5.296944E+02	; C45-C44-C97, prebuilt c-c3-c3
45	44	157	1	108.220	3.932960E+02	; C45-C44-H157, prebuilt c-c3-h1
97	44	157	1	109.560	3.882752E+02	; C97-C44-H157, prebuilt c3-c3-h1
44	45	46	1	115.180	5.589824E+02	; C44-C45-N46, prebuilt c3-c-n
44	45	96	1	123.200	5.640032E+02	; C44-C45-O96, prebuilt c3-c-o
46	45	96	1	123.050	6.209056E+02	; N46-C45-O96, prebuilt n-c-o
45	46	47	1	120.690	5.305312E+02	; C45-N46-C47, prebuilt c-n-c3
45	46	158	1	117.550	4.041744E+02	; C45-N46-H158, prebuilt c-n-hn
47	46	158	1	117.680	3.832544E+02	; C47-N46-H158, prebuilt c3-n-hn
46	47	48	1	109.060	5.606560E+02	; N46-C47-C48, prebuilt c-c3-n
46	47	95	1	111.610	5.514512E+02	; N46-C47-C95, prebuilt c3-c3-n
46	47	159	1	108.880	4.167264E+02	; N46-C47-H159, prebuilt h1-c3-n
48	47	95	1	111.040	5.296944E+02	; C48-C47-C95, prebuilt c-c3-c3
48	47	159	1	108.220	3.932960E+02	; C48-C47-H159, prebuilt c-c3-h1
95	47	159	1	109.560	3.882752E+02	; C95-C47-H159, prebuilt c3-c3-h1
47	48	49	1	115.180	5.589824E+02	; C47-C48-N49, prebuilt c3-c-n
47	48	94	1	123.200	5.640032E+02	; C47-C48-O94, prebuilt c3-c-o
49	48	94	1	123.050	6.209056E+02	; N49-C48-O94, prebuilt n-c-o

48	49	50	1	120.690	5.305312E+02	; C48-N49-C50, prebuilt c-n-c3
48	49	160	1	117.550	4.041744E+02	; C48-N49-H160, prebuilt c-n-hn
50	49	160	1	117.680	3.832544E+02	; C50-N49-H160, prebuilt c3-n-hn
49	50	51	1	109.060	5.606560E+02	; N49-C50-C51, prebuilt c-c3-n
49	50	90	1	111.610	5.514512E+02	; N49-C50-C90, prebuilt c3-c3-n
49	50	161	1	108.880	4.167264E+02	; N49-C50-H161, prebuilt h1-c3-n
51	50	90	1	111.040	5.296944E+02	; C51-C50-C90, prebuilt c-c3-c3
51	50	161	1	108.220	3.932960E+02	; C51-C50-H161, prebuilt c-c3-h1
90	50	161	1	109.560	3.882752E+02	; C90-C50-H161, prebuilt c3-c3-h1
50	51	52	1	115.180	5.589824E+02	; C50-C51-N52, prebuilt c3-c-n
50	51	89	1	123.200	5.640032E+02	; C50-C51-O89, prebuilt c3-c-o
52	51	89	1	123.050	6.209056E+02	; N52-C51-O89, prebuilt n-c-o
51	52	53	1	120.690	5.305312E+02	; C51-N52-C53, prebuilt c-n-c3
51	52	162	1	117.550	4.041744E+02	; C51-N52-H162, prebuilt c-n-hn
53	52	162	1	117.680	3.832544E+02	; C53-N52-H162, prebuilt c3-n-hn
52	53	54	1	109.060	5.606560E+02	; N52-C53-C54, prebuilt c-c3-n
52	53	163	1	108.880	4.167264E+02	; N52-C53-H163, prebuilt h1-c3-n
52	53	164	1	108.880	4.167264E+02	; N52-C53-H164, prebuilt h1-c3-n
54	53	163	1	108.220	3.932960E+02	; C54-C53-H163, prebuilt c-c3-h1
54	53	164	1	108.220	3.932960E+02	; C54-C53-H164, prebuilt c-c3-h1
163	53	164	1	108.460	3.280256E+02	; H163-C53-H164, prebuilt h1-c3-h1

53	54	55	1	115.180	5.589824E+02	; C53-C54-N55, prebuilt c3-c-n
53	54	88	1	123.200	5.640032E+02	; C53-C54-O88, prebuilt c3-c-o
55	54	88	1	123.050	6.209056E+02	; N55-C54-O88, prebuilt n-c-o
54	55	56	1	120.690	5.305312E+02	; C54-N55-C56, prebuilt c-n-c3
54	55	165	1	117.550	4.041744E+02	; C54-N55-H165, prebuilt c-n-hn
56	55	165	1	117.680	3.832544E+02	; C56-N55-H165, prebuilt c3-n-hn
55	56	57	1	109.060	5.606560E+02	; N55-C56-C57, prebuilt c-c3-n
55	56	86	1	111.610	5.514512E+02	; N55-C56-C86, prebuilt c3-c3-n
55	56	166	1	108.880	4.167264E+02	; N55-C56-H166, prebuilt h1-c3-n
57	56	86	1	111.040	5.296944E+02	; C57-C56-C86, prebuilt c-c3-c3
57	56	166	1	108.220	3.932960E+02	; C57-C56-H166, prebuilt c-c3-h1
86	56	166	1	109.560	3.882752E+02	; C86-C56-H166, prebuilt c3-c3-h1
56	57	58	1	115.180	5.589824E+02	; C56-C57-N58, prebuilt c3-c-n
56	57	85	1	123.200	5.640032E+02	; C56-C57-O85, prebuilt c3-c-o
58	57	85	1	123.050	6.209056E+02	; N58-C57-O85, prebuilt n-c-o
57	58	59	1	120.690	5.305312E+02	; C57-N58-C59, prebuilt c-n-c3
57	58	167	1	117.550	4.041744E+02	; C57-N58-H167, prebuilt c-n-hn
59	58	167	1	117.680	3.832544E+02	; C59-N58-H167, prebuilt c3-n-hn
58	59	60	1	109.060	5.606560E+02	; N58-C59-C60, prebuilt c-c3-n
58	59	79	1	111.610	5.514512E+02	; N58-C59-C79, prebuilt c3-c3-n
58	59	168	1	108.880	4.167264E+02	; N58-C59-H168, prebuilt h1-c3-n

60	59	79	1	111.040	5.296944E+02	; C60-C59-C79, prebuilt c-c3-c3
60	59	168	1	108.220	3.932960E+02	; C60-C59-H168, prebuilt c-c3-h1
79	59	168	1	109.560	3.882752E+02	; C79-C59-H168, prebuilt c3-c3-h1
59	60	61	1	115.180	5.589824E+02	; C59-C60-N61, prebuilt c3-c-n
59	60	78	1	123.200	5.640032E+02	; C59-C60-O78, prebuilt c3-c-o
61	60	78	1	123.050	6.209056E+02	; N61-C60-O78, prebuilt n-c-o
60	61	62	1	120.690	5.305312E+02	; C60-N61-C62, prebuilt c-n-c3
60	61	169	1	117.550	4.041744E+02	; C60-N61-H169, prebuilt c-n-hn
62	61	169	1	117.680	3.832544E+02	; C62-N61-H169, prebuilt c3-n-hn
61	62	63	1	109.060	5.606560E+02	; N61-C62-C63, prebuilt c-c3-n
61	62	68	1	111.610	5.514512E+02	; N61-C62-C68, prebuilt c3-c3-n
61	62	170	1	108.880	4.167264E+02	; N61-C62-H170, prebuilt h1-c3-n
63	62	68	1	111.040	5.296944E+02	; C63-C62-C68, prebuilt c-c3-c3
63	62	170	1	108.220	3.932960E+02	; C63-C62-H170, prebuilt c-c3-h1
68	62	170	1	109.560	3.882752E+02	; C68-C62-H170, prebuilt c3-c3-h1
62	63	64	1	110.720	5.765552E+02	; C62-C63-O64, prebuilt c3-c-os
62	63	67	1	123.200	5.640032E+02	; C62-C63-O67, prebuilt c3-c-o
64	63	67	1	123.250	6.301104E+02	; O64-C63-O67, prebuilt o-c-os
63	64	65	1	115.980	5.296944E+02	; C63-O64-C65, prebuilt c-os-c3
33	65	64	1	107.970	5.690240E+02	; C33-C65-O64, prebuilt c3-c3-os
33	65	66	1	111.510	5.263472E+02	; C33-C65-C66, prebuilt c3-c3-c3

33 c3-h1	65	171	1	109.560	3.882752E+02	; C33-C65-H171, prebuilt c3-
64 c3-os	65	66	1	107.970	5.690240E+02	; O64-C65-C66, prebuilt c3-
64 c3-os	65	171	1	109.780	4.250944E+02	; O64-C65-H171, prebuilt h1-
66 c3-h1	65	171	1	109.560	3.882752E+02	; C66-C65-H171, prebuilt c3-
65 c3-hc	66	172	1	109.800	3.874384E+02	; C65-C66-H172, prebuilt c3-
65 c3-hc	66	173	1	109.800	3.874384E+02	; C65-C66-H173, prebuilt c3-
65 c3-hc	66	174	1	109.800	3.874384E+02	; C65-C66-H174, prebuilt c3-
172 hc-c3-hc	66	173	1	107.580	3.296992E+02	; H172-C66-H173, prebuilt
172 hc-c3-hc	66	174	1	107.580	3.296992E+02	; H172-C66-H174, prebuilt
173 hc-c3-hc	66	174	1	107.580	3.296992E+02	; H173-C66-H174, prebuilt
62 c3	68	69	1	111.040	5.296944E+02	; C62-C68-C69, prebuilt c-c3-
62 c3-hc	68	175	1	109.800	3.874384E+02	; C62-C68-H175, prebuilt c3-
62 c3-hc	68	176	1	109.800	3.874384E+02	; C62-C68-H176, prebuilt c3-
69 c3-hc	68	175	1	108.770	3.924592E+02	; C69-C68-H175, prebuilt c-
69 c3-hc	68	176	1	108.770	3.924592E+02	; C69-C68-H176, prebuilt c-
175 hc-c3-hc	68	176	1	107.580	3.296992E+02	; H175-C68-H176, prebuilt
68 ca	69	70	1	118.400	5.204896E+02	; C68-C69-C70, prebuilt c3-c-
68 o	69	77	1	123.200	5.640032E+02	; C68-C69-O77, prebuilt c3-c-
70 o	69	77	1	122.600	5.748816E+02	; C70-C69-O77, prebuilt ca-c-
69 ca	70	71	1	120.330	5.380624E+02	; C69-C70-C71, prebuilt c-ca-
69 ca	70	75	1	120.330	5.380624E+02	; C69-C70-C75, prebuilt c-ca-

71<br>ca-ca	70	75	1	120.020	5.573088E+02	; C71-C70-C75, prebuilt ca-
70<br>ca-ca	71	72	1	120.020	5.573088E+02	; C70-C71-C72, prebuilt ca-
70<br>ca-nh	71	76	1	120.950	5.715344E+02	; C70-C71-N76, prebuilt ca-
72<br>ca-nh	71	76	1	120.950	5.715344E+02	; C72-C71-N76, prebuilt ca-
71<br>ca-ca	72	73	1	120.020	5.573088E+02	; C71-C72-C73, prebuilt ca-
71<br>ca-ha	72	177	1	119.880	4.033376E+02	; C71-C72-H177, prebuilt ca-
73<br>ca-ha	72	177	1	119.880	4.033376E+02	; C73-C72-H177, prebuilt ca-
72<br>ca-ca	73	74	1	120.020	5.573088E+02	; C72-C73-C74, prebuilt ca-
72<br>ca-ha	73	178	1	119.880	4.033376E+02	; C72-C73-H178, prebuilt ca-
74<br>ca-ha	73	178	1	119.880	4.033376E+02	; C74-C73-H178, prebuilt ca-
73<br>ca-ca	74	75	1	120.020	5.573088E+02	; C73-C74-C75, prebuilt ca-
73<br>ca-ha	74	179	1	119.880	4.033376E+02	; C73-C74-H179, prebuilt ca-
75<br>ca-ha	74	179	1	119.880	4.033376E+02	; C75-C74-H179, prebuilt ca-
70<br>ca-ca	75	74	1	120.020	5.573088E+02	; C70-C75-C74, prebuilt ca-
70<br>ca-ha	75	180	1	119.880	4.033376E+02	; C70-C75-H180, prebuilt ca-
74<br>ca-ha	75	180	1	119.880	4.033376E+02	; C74-C75-H180, prebuilt ca-
71<br>nh-hn	76	181	1	116.070	4.050112E+02	; C71-N76-H181, prebuilt ca-
71<br>nh-hn	76	182	1	116.070	4.050112E+02	; C71-N76-H182, prebuilt ca-
181<br>hn-nh-hn	76	182	1	115.120	3.355568E+02	; H181-N76-H182, prebuilt
59<br>c3-c3	79	80	1	111.510	5.263472E+02	; C59-C79-C80, prebuilt c3-
59<br>c3-c3	79	81	1	111.510	5.263472E+02	; C59-C79-C81, prebuilt c3-

59	79	183	1	109.800	3.874384E+02	; C59-C79-H183, prebuilt c3-c3-hc
80	79	81	1	111.510	5.263472E+02	; C80-C79-C81, prebuilt c3-c3-c3
80	79	183	1	109.800	3.874384E+02	; C80-C79-H183, prebuilt c3-c3-hc
81	79	183	1	109.800	3.874384E+02	; C81-C79-H183, prebuilt c3-c3-hc
79	80	184	1	109.800	3.874384E+02	; C79-C80-H184, prebuilt c3-c3-hc
79	80	185	1	109.800	3.874384E+02	; C79-C80-H185, prebuilt c3-c3-hc
79	80	186	1	109.800	3.874384E+02	; C79-C80-H186, prebuilt c3-c3-hc
184	80	185	1	107.580	3.296992E+02	; H184-C80-H185, prebuilt hc-c3-hc
184	80	186	1	107.580	3.296992E+02	; H184-C80-H186, prebuilt hc-c3-hc
185	80	186	1	107.580	3.296992E+02	; H185-C80-H186, prebuilt hc-c3-hc
79	81	82	1	111.040	5.296944E+02	; C79-C81-C82, prebuilt c-c3-c3
79	81	187	1	109.800	3.874384E+02	; C79-C81-H187, prebuilt c3-c3-hc
79	81	188	1	109.800	3.874384E+02	; C79-C81-H188, prebuilt c3-c3-hc
82	81	187	1	108.770	3.924592E+02	; C82-C81-H187, prebuilt c-c3-hc
82	81	188	1	108.770	3.924592E+02	; C82-C81-H188, prebuilt c-c3-hc
187	81	188	1	107.580	3.296992E+02	; H187-C81-H188, prebuilt hc-c3-hc
81	82	83	1	112.730	5.723712E+02	; C81-C82-O83, prebuilt c3-c-oh
81	82	84	1	123.200	5.640032E+02	; C81-C82-O84, prebuilt c3-c-o
83	82	84	1	122.100	6.351312E+02	; O83-C82-O84, prebuilt o-c-oh
82	83	189	1	106.550	4.175632E+02	; C82-O83-H189, prebuilt c-oh-ho
56	86	87	1	110.190	5.648400E+02	; C56-C86-O87, prebuilt c3-c3-oh

https://doi.org/10.3390/pharmaceutics18060657

56	86	190	1	109.560	3.882752E+02	; C56-C86-H190, prebuilt c3-c3-h1
56	86	191	1	109.560	3.882752E+02	; C56-C86-H191, prebuilt c3-c3-h1
87	86	190	1	110.260	4.259312E+02	; O87-C86-H190, prebuilt h1-c3-oh
87	86	191	1	110.260	4.259312E+02	; O87-C86-H191, prebuilt h1-c3-oh
190	86	191	1	108.460	3.280256E+02	; H190-C86-H191, prebuilt h1-c3-h1
86	87	192	1	107.260	3.966432E+02	; C86-O87-H192, prebuilt c3-oh-ho
50	90	91	1	111.040	5.296944E+02	; C50-C90-C91, prebuilt c-c3-c3
50	90	193	1	109.800	3.874384E+02	; C50-C90-H193, prebuilt c3-c3-hc
50	90	194	1	109.800	3.874384E+02	; C50-C90-H194, prebuilt c3-c3-hc
91	90	193	1	108.770	3.924592E+02	; C91-C90-H193, prebuilt c-c3-hc
91	90	194	1	108.770	3.924592E+02	; C91-C90-H194, prebuilt c-c3-hc
193	90	194	1	107.580	3.296992E+02	; H193-C90-H194, prebuilt hc-c3-hc
90	91	92	1	123.200	5.640032E+02	; C90-C91-O92, prebuilt c3-c-o
90	91	93	1	112.730	5.723712E+02	; C90-C91-O93, prebuilt c3-c-oh
92	91	93	1	122.100	6.351312E+02	; O92-C91-O93, prebuilt o-c-oh
91	93	195	1	106.550	4.175632E+02	; C91-O93-H195, prebuilt c-oh-ho
47	95	196	1	109.800	3.874384E+02	; C47-C95-H196, prebuilt c3-c3-hc
47	95	197	1	109.800	3.874384E+02	; C47-C95-H197, prebuilt c3-c3-hc
47	95	198	1	109.800	3.874384E+02	; C47-C95-H198, prebuilt c3-c3-hc
196	95	197	1	107.580	3.296992E+02	; H196-C95-H197, prebuilt hc-c3-hc
196	95	198	1	107.580	3.296992E+02	; H196-C95-H198, prebuilt hc-c3-hc

197<br>hc-c3-hc	95	198	1	107.580	3.296992E+02	; H197-C95-H198, prebuilt
44<br>c3	97	98	1	111.040	5.296944E+02	; C44-C97-C98, prebuilt c-c3-
44<br>c3-hc	97	199	1	109.800	3.874384E+02	; C44-C97-H199, prebuilt c3-
44<br>c3-hc	97	200	1	109.800	3.874384E+02	; C44-C97-H200, prebuilt c3-
98<br>c3-hc	97	199	1	108.770	3.924592E+02	; C98-C97-H199, prebuilt c-
98<br>c3-hc	97	200	1	108.770	3.924592E+02	; C98-C97-H200, prebuilt c-
199<br>hc-c3-hc	97	200	1	107.580	3.296992E+02	; H199-C97-H200, prebuilt
97<br>oh	98	99	1	112.730	5.723712E+02	; C97-C98-O99, prebuilt c3-c-
97<br>c-o	98	100	1	123.200	5.640032E+02	; C97-C98-O100, prebuilt c3-
99<br>oh	98	100	1	122.100	6.351312E+02	; O99-C98-O100, prebuilt o-c-
98<br>oh-ho	99	201	1	106.550	4.175632E+02	; C98-O99-H201, prebuilt c-
40<br>c3-c3	101	102	1	111.510	5.263472E+02	; C40-C101-C102, prebuilt c3-
40<br>c3-c3-hc	101	202	1	109.800	3.874384E+02	; C40-C101-H202, prebuilt
40<br>c3-c3-hc	101	203	1	109.800	3.874384E+02	; C40-C101-H203, prebuilt
102<br>c3-c3-hc	101	202	1	109.800	3.874384E+02	; C102-C101-H202, prebuilt
102<br>c3-c3-hc	101	203	1	109.800	3.874384E+02	; C102-C101-H203, prebuilt
202<br>hc-c3-hc	101	203	1	107.580	3.296992E+02	; H202-C101-H203, prebuilt
101<br>c3-c3-c3	102	103	1	111.510	5.263472E+02	; C101-C102-C103, prebuilt
101<br>c3-c3-hc	102	204	1	109.800	3.874384E+02	; C101-C102-H204, prebuilt
101<br>c3-c3-hc	102	205	1	109.800	3.874384E+02	; C101-C102-H205, prebuilt
103<br>c3-c3-hc	102	204	1	109.800	3.874384E+02	; C103-C102-H204, prebuilt

https://doi.org/10.3390/pharmaceutics18060657

103<br>c3-c3-hc	102	205	1	109.800	3.874384E+02	; C103-C102-H205, prebuilt
204<br>hc-c3-hc	102	205	1	107.580	3.296992E+02	; H204-C102-H205, prebuilt
102<br>c3-c3-n3	103	104	1	111.040	5.522880E+02	; C102-C103-N104, prebuilt
102<br>c3-c3-h1	103	206	1	109.560	3.882752E+02	; C102-C103-H206, prebuilt
102<br>c3-c3-h1	103	207	1	109.560	3.882752E+02	; C102-C103-H207, prebuilt
104<br>h1-c3-n3	103	206	1	109.880	4.142160E+02	; N104-C103-H206, prebuilt
104<br>h1-c3-n3	103	207	1	109.880	4.142160E+02	; N104-C103-H207, prebuilt
206<br>h1-c3-h1	103	207	1	108.460	3.280256E+02	; H206-C103-H207, prebuilt
103<br>c3-n3-hn	104	208	1	109.290	3.966432E+02	; C103-N104-H208, prebuilt
103<br>c3-n3-hn	104	209	1	109.290	3.966432E+02	; C103-N104-H209, prebuilt
208<br>hn-n3-hn	104	209	1	106.400	3.464352E+02	; H208-N104-H209, prebuilt
30<br>c3-c3	107	108	1	111.040	5.296944E+02	; C30-C107-C108, prebuilt c-
30<br>c3-c3-hc	107	210	1	109.800	3.874384E+02	; C30-C107-H210, prebuilt
30<br>c3-c3-hc	107	211	1	109.800	3.874384E+02	; C30-C107-H211, prebuilt
108<br>c-c3-hc	107	210	1	108.770	3.924592E+02	; C108-C107-H210, prebuilt
108<br>c-c3-hc	107	211	1	108.770	3.924592E+02	; C108-C107-H211, prebuilt
210<br>hc-c3-hc	107	211	1	107.580	3.296992E+02	; H210-C107-H211, prebuilt
107<br>c3-c-o	108	109	1	123.200	5.640032E+02	; C107-C108-O109, prebuilt
107<br>c3-c-oh	108	110	1	112.730	5.723712E+02	; C107-C108-O110, prebuilt
109<br>o-c-oh	108	110	1	122.100	6.351312E+02	; O109-C108-O110, prebuilt
108<br>c-oh-ho	110	212	1	106.550	4.175632E+02	; C108-O110-H212, prebuilt

https://doi.org/10.3390/pharmaceutics18060657

26	111	112	1	111.040	5.296944E+02	; C26-C111-C112, prebuilt c-c3-c3
26	111	213	1	109.800	3.874384E+02	; C26-C111-H213, prebuilt c3-c3-hc
26	111	214	1	109.800	3.874384E+02	; C26-C111-H214, prebuilt c3-c3-hc
112	111	213	1	108.770	3.924592E+02	; C112-C111-H213, prebuilt c-c3-hc
112	111	214	1	108.770	3.924592E+02	; C112-C111-H214, prebuilt c-c3-hc
213	111	214	1	107.580	3.296992E+02	; H213-C111-H214, prebuilt hc-c3-hc
111	112	113	1	115.180	5.589824E+02	; C111-C112-N113, prebuilt c3-c-n
111	112	114	1	123.200	5.640032E+02	; C111-C112-O114, prebuilt c3-c-o
113	112	114	1	123.050	6.209056E+02	; N113-C112-O114, prebuilt n-c-o
112	113	215	1	117.550	4.041744E+02	; C112-N113-H215, prebuilt c-n-hn
112	113	216	1	117.550	4.041744E+02	; C112-N113-H216, prebuilt c-n-hn
215	113	216	1	117.950	3.313728E+02	; H215-N113-H216, prebuilt hn-n-hn

[dihedrals] ; propers								
; atom_i	atom_j	atom_k	atom_l	functype	d0 (Deg.)	k (kJ/mol/rad^2)		; Case of functype=2
; atom_i	atom_j	atom_k	atom_l	functype	phase (Deg.)	kd (kJ/mol)	pn	; Case of functype=9
1	2	3	4	9	180.000	15.16700	2	; C1-C2-C3-C4, prebuilt X-ca-ca-X
1	2	3	9	9	180.000	15.16700	2	; C1-C2-C3-C9, prebuilt X-ca-ca-X
1	6	5	4	9	180.000	15.16700	2	; C1-C6-C5-C4, prebuilt X-ca-ca-X
1	6	5	118	9	180.000	15.16700	2	; C1-C6-C5-H118, prebuilt X-ca-ca-X
2	1	6	5	9	180.000	15.16700	2	; C2-C1-C6-C5, prebuilt X-ca-ca-X
2	1	6	119	9	180.000	15.16700	2	; C2-C1-C6-H119, prebuilt X-ca-ca-X
2	3	4	5	9	180.000	15.16700	2	; C2-C3-C4-C5, prebuilt X-ca-ca-X

2	3	4	7	9	180.000	15.16700	2	; C2-C3-C4-N7, prebuilt X-ca-ca-X
2	3	9	8	9	180.000	2.92880	2	; C2-C3-C9-C8, prebuilt X-c2-ca-X
2	3	9	10	9	180.000	2.92880	2	; C2-C3-C9-C10, prebuilt X-c2-ca-X
3	2	1	6	9	180.000	15.16700	2	; C3-C2-C1-C6, prebuilt X-ca-ca-X
3	2	1	116	9	180.000	15.16700	2	; C3-C2-C1-H116, prebuilt X-ca-ca-X
3	4	5	6	9	180.000	15.16700	2	; C3-C4-C5-C6, prebuilt X-ca-ca-X
3	4	5	118	9	180.000	15.16700	2	; C3-C4-C5-H118, prebuilt X-ca-ca-X
3	4	7	8	9	180.000	1.25520	2	; C3-C4-N7-C8, prebuilt X-ca-na-X
3	4	7	120	9	180.000	1.25520	2	; C3-C4-N7-H120, prebuilt X-ca-na-X
3	9	8	7	9	180.000	27.82360	2	; C3-C9-C8-N7, prebuilt X-c2-c2-X
3	9	8	121	9	180.000	27.82360	2	; C3-C9-C8-H121, prebuilt X-c2-c2-X
3	9	10	11	9	0.000	0.00000	2	; C3-C9-C10-C11, prebuilt X-c2-c3-X
3	9	10	122	9	0.000	0.00000	2	; C3-C9-C10-H122, prebuilt X-c2-c3-X
3	9	10	123	9	0.000	0.00000	2	; C3-C9-C10-H123, prebuilt X-c2-c3-X
4	3	2	117	9	180.000	15.16700	2	; C4-C3-C2-H117, prebuilt X-ca-ca-X
4	3	9	8	9	180.000	2.92880	2	; C4-C3-C9-C8, prebuilt X-c2-ca-X
4	3	9	10	9	180.000	2.92880	2	; C4-C3-C9-C10, prebuilt X-c2-ca-X
4	5	6	119	9	180.000	15.16700	2	; C4-C5-C6-H119, prebuilt X-ca-ca-X
4	7	8	9	9	180.000	2.61500	2	; C4-N7-C8-C9, prebuilt X-c2-na-X
4	7	8	121	9	180.000	2.61500	2	; C4-N7-C8-H121, prebuilt X-c2-na-X
5	4	3	9	9	180.000	15.16700	2	; C5-C4-C3-C9, prebuilt X-ca-ca-X

5	4	7	8	9	180.000	1.25520	2	; C5-C4-N7-C8, prebuilt X-ca-na-X
5	4	7	120	9	180.000	1.25520	2	; C5-C4-N7-H120, prebuilt X-ca-na-X
5	6	1	116	9	180.000	15.16700	2	; C5-C6-C1-H116, prebuilt X-ca-ca-X
6	1	2	117	9	180.000	15.16700	2	; C6-C1-C2-H117, prebuilt X-ca-ca-X
6	5	4	7	9	180.000	15.16700	2	; C6-C5-C4-N7, prebuilt X-ca-ca-X
7	4	3	9	9	180.000	15.16700	2	; N7-C4-C3-C9, prebuilt X-ca-ca-X
7	4	5	118	9	180.000	15.16700	2	; N7-C4-C5-H118, prebuilt X-ca-ca-X
7	8	9	10	9	180.000	27.82360	2	; N7-C8-C9-C10, prebuilt X-c2-c2-X
8	9	10	11	9	0.000	0.00000	2	; C8-C9-C10-C11, prebuilt X-c2-c3-X
8	9	10	122	9	180.000	1.58992	3	; C8-C9-C10-H122, prebuilt hc-c3-c2-c2
8	9	10	122	9	0.000	0.00000	2	; C8-C9-C10-H122, prebuilt hc-c3-c2-c2
8	9	10	122	9	0.000	4.81160	1	; C8-C9-C10-H122, prebuilt hc-c3-c2-c2
8	9	10	123	9	180.000	1.58992	3	; C8-C9-C10-H123, prebuilt hc-c3-c2-c2
8	9	10	123	9	0.000	0.00000	2	; C8-C9-C10-H123, prebuilt hc-c3-c2-c2
8	9	10	123	9	0.000	4.81160	1	; C8-C9-C10-H123, prebuilt hc-c3-c2-c2
9	3	2	117	9	180.000	15.16700	2	; C9-C3-C2-H117, prebuilt X-ca-ca-X
9	8	7	120	9	180.000	2.61500	2	; C9-C8-N7-H120, prebuilt X-c2-na-X
9	10	11	12	9	0.000	0.65084	3	; C9-C10-C11-N12, prebuilt X-c3-c3-X
9	10	11	24	9	0.000	0.65084	3	; C9-C10-C11-C24, prebuilt X-c3-c3-X
9	10	11	124	9	0.000	0.65084	3	; C9-C10-C11-H124, prebuilt X-c3-c3-X
10	9	8	121	9	180.000	27.82360	2	; C10-C9-C8-H121, prebuilt X-c2-c2-X

10	11	12	13	9	180.000	2.09200	4	; C10-C11-N12-C13, prebuilt c3-c3-n-c
10	11	12	13	9	180.000	0.62760	3	; C10-C11-N12-C13, prebuilt c3-c3-n-c
10	11	12	13	9	0.000	0.00000	2	; C10-C11-N12-C13, prebuilt c3-c3-n-c
10	11	12	13	9	0.000	2.21752	1	; C10-C11-N12-C13, prebuilt c3-c3-n-c
10	11	12	125	9	0.000	0.00000	2	; C10-C11-N12-H125, prebuilt X-c3-n-X
10	11	24	25	9	0.000	0.41840	4	; C10-C11-C24-N25, prebuilt c3-c3-c-n
10	11	24	25	9	0.000	0.29288	2	; C10-C11-C24-N25, prebuilt c3-c3-c-n
10	11	24	115	9	180.000	0.00000	2	; C10-C11-C24-O115, prebuilt X-c-c3-X
11	12	13	14	9	180.000	10.46000	2	; C11-N12-C13-O14, prebuilt X-c-n-X
11	12	13	15	9	0.000	0.00000	2	; C11-N12-C13-C15, prebuilt c3-c-n-c3
11	12	13	15	9	180.000	6.27600	1	; C11-N12-C13-C15, prebuilt c3-c-n-c3
11	24	25	26	9	0.000	0.00000	2	; C11-C24-N25-C26, prebuilt c3-c-n-c3
11	24	25	26	9	180.000	6.27600	1	; C11-C24-N25-C26, prebuilt c3-c-n-c3
11	24	25	145	9	180.000	10.46000	2	; C11-C24-N25-H145, prebuilt X-c-n-X
12	11	10	122	9	0.000	0.65084	3	; N12-C11-C10-H122, prebuilt X-c3-c3-X
12	11	10	123	9	0.000	0.65084	3	; N12-C11-C10-H123, prebuilt X-c3-c3-X
12	11	24	25	9	180.000	7.11280	1	; N12-C11-C24-N25, prebuilt n-c3-c-n
12	11	24	25	9	180.000	8.36800	2	; N12-C11-C24-N25, prebuilt n-c3-c-n
12	11	24	115	9	180.000	0.00000	2	; N12-C11-C24-O115, prebuilt X-c-c3-X
12	13	15	16	9	0.000	0.41840	4	; N12-C13-C15-C16, prebuilt c3-c3-c-n
12	13	15	16	9	0.000	0.29288	2	; N12-C13-C15-C16, prebuilt c3-c3-c-n

12	13	15	126	9	180.000	0.00000	2	; N12-C13-C15-H126, prebuilt X-c-c3-X
12	13	15	127	9	180.000	0.00000	2	; N12-C13-C15-H127, prebuilt X-c-c3-X
13	12	11	24	9	180.000	3.55640	2	; C13-N12-C11-C24, prebuilt c-n-c3-c
13	12	11	24	9	0.000	3.34720	1	; C13-N12-C11-C24, prebuilt c-n-c3-c
13	12	11	124	9	0.000	0.00000	2	; C13-N12-C11-H124, prebuilt X-c3-n-X
13	15	16	17	9	0.000	0.65084	3	; C13-C15-C16-C17, prebuilt X-c3-c3-X
13	15	16	128	9	0.000	0.65084	3	; C13-C15-C16-H128, prebuilt X-c3-c3-X
13	15	16	129	9	0.000	0.65084	3	; C13-C15-C16-H129, prebuilt X-c3-c3-X
14	13	12	125	9	180.000	10.46000	2	; O14-C13-N12-H125, prebuilt hn-n-c-o
14	13	12	125	9	0.000	8.36800	1	; O14-C13-N12-H125, prebuilt hn-n-c-o
14	13	15	16	9	180.000	0.00000	2	; O14-C13-C15-C16, prebuilt X-c-c3-X
14	13	15	126	9	0.000	3.34720	1	; O14-C13-C15-H126, prebuilt hc-c3-c-o
14	13	15	126	9	0.000	0.00000	2	; O14-C13-C15-H126, prebuilt hc-c3-c-o
14	13	15	126	9	180.000	0.33472	3	; O14-C13-C15-H126, prebuilt hc-c3-c-o
14	13	15	127	9	0.000	3.34720	1	; O14-C13-C15-H127, prebuilt hc-c3-c-o
14	13	15	127	9	0.000	0.00000	2	; O14-C13-C15-H127, prebuilt hc-c3-c-o
14	13	15	127	9	180.000	0.33472	3	; O14-C13-C15-H127, prebuilt hc-c3-c-o
15	13	12	125	9	180.000	10.46000	2	; C15-C13-N12-H125, prebuilt X-c-n-X
15	16	17	18	9	0.000	0.75312	3	; C15-C16-C17-C18, prebuilt c3-c3-c3-c3
15	16	17	18	9	180.000	1.04600	2	; C15-C16-C17-C18, prebuilt c3-c3-c3-c3
15	16	17	18	9	180.000	0.83680	1	; C15-C16-C17-C18, prebuilt c3-c3-c3-c3

15	16	17	130	9	0.000	0.66944	3	; C15-C16-C17-H130, prebuilt hc-c3-c3-c3
15	16	17	131	9	0.000	0.66944	3	; C15-C16-C17-H131, prebuilt hc-c3-c3-c3
16	17	18	19	9	0.000	0.75312	3	; C16-C17-C18-C19, prebuilt c3-c3-c3-c3
16	17	18	19	9	180.000	1.04600	2	; C16-C17-C18-C19, prebuilt c3-c3-c3-c3
16	17	18	19	9	180.000	0.83680	1	; C16-C17-C18-C19, prebuilt c3-c3-c3-c3
16	17	18	132	9	0.000	0.66944	3	; C16-C17-C18-H132, prebuilt hc-c3-c3-c3
16	17	18	133	9	0.000	0.66944	3	; C16-C17-C18-H133, prebuilt hc-c3-c3-c3
17	16	15	126	9	0.000	0.66944	3	; C17-C16-C15-H126, prebuilt hc-c3-c3-c3
17	16	15	127	9	0.000	0.66944	3	; C17-C16-C15-H127, prebuilt hc-c3-c3-c3
17	18	19	20	9	0.000	0.75312	3	; C17-C18-C19-C20, prebuilt c3-c3-c3-c3
17	18	19	20	9	180.000	1.04600	2	; C17-C18-C19-C20, prebuilt c3-c3-c3-c3
17	18	19	20	9	180.000	0.83680	1	; C17-C18-C19-C20, prebuilt c3-c3-c3-c3
17	18	19	134	9	0.000	0.66944	3	; C17-C18-C19-H134, prebuilt hc-c3-c3-c3
17	18	19	135	9	0.000	0.66944	3	; C17-C18-C19-H135, prebuilt hc-c3-c3-c3
18	17	16	128	9	0.000	0.66944	3	; C18-C17-C16-H128, prebuilt hc-c3-c3-c3
18	17	16	129	9	0.000	0.66944	3	; C18-C17-C16-H129, prebuilt hc-c3-c3-c3
18	19	20	21	9	0.000	0.75312	3	; C18-C19-C20-C21, prebuilt c3-c3-c3-c3
18	19	20	21	9	180.000	1.04600	2	; C18-C19-C20-C21, prebuilt c3-c3-c3-c3
18	19	20	21	9	180.000	0.83680	1	; C18-C19-C20-C21, prebuilt c3-c3-c3-c3
18	19	20	136	9	0.000	0.66944	3	; C18-C19-C20-H136, prebuilt hc-c3-c3-c3
18	19	20	137	9	0.000	0.66944	3	; C18-C19-C20-H137, prebuilt hc-c3-c3-c3

19	18	17	130	9	0.000	0.66944	3	; C19-C18-C17-H130, prebuilt hc-c3-c3-c3
19	18	17	131	9	0.000	0.66944	3	; C19-C18-C17-H131, prebuilt hc-c3-c3-c3
19	20	21	22	9	0.000	0.75312	3	; C19-C20-C21-C22, prebuilt c3-c3-c3-c3
19	20	21	22	9	180.000	1.04600	2	; C19-C20-C21-C22, prebuilt c3-c3-c3-c3
19	20	21	22	9	180.000	0.83680	1	; C19-C20-C21-C22, prebuilt c3-c3-c3-c3
19	20	21	138	9	0.000	0.66944	3	; C19-C20-C21-H138, prebuilt hc-c3-c3-c3
19	20	21	139	9	0.000	0.66944	3	; C19-C20-C21-H139, prebuilt hc-c3-c3-c3
20	19	18	132	9	0.000	0.66944	3	; C20-C19-C18-H132, prebuilt hc-c3-c3-c3
20	19	18	133	9	0.000	0.66944	3	; C20-C19-C18-H133, prebuilt hc-c3-c3-c3
20	21	22	23	9	0.000	0.75312	3	; C20-C21-C22-C23, prebuilt c3-c3-c3-c3
20	21	22	23	9	180.000	1.04600	2	; C20-C21-C22-C23, prebuilt c3-c3-c3-c3
20	21	22	23	9	180.000	0.83680	1	; C20-C21-C22-C23, prebuilt c3-c3-c3-c3
20	21	22	140	9	0.000	0.66944	3	; C20-C21-C22-H140, prebuilt hc-c3-c3-c3
20	21	22	141	9	0.000	0.66944	3	; C20-C21-C22-H141, prebuilt hc-c3-c3-c3
21	20	19	134	9	0.000	0.66944	3	; C21-C20-C19-H134, prebuilt hc-c3-c3-c3
21	20	19	135	9	0.000	0.66944	3	; C21-C20-C19-H135, prebuilt hc-c3-c3-c3
21	22	23	142	9	0.000	0.66944	3	; C21-C22-C23-H142, prebuilt hc-c3-c3-c3
21	22	23	143	9	0.000	0.66944	3	; C21-C22-C23-H143, prebuilt hc-c3-c3-c3
21	22	23	144	9	0.000	0.66944	3	; C21-C22-C23-H144, prebuilt hc-c3-c3-c3
22	21	20	136	9	0.000	0.66944	3	; C22-C21-C20-H136, prebuilt hc-c3-c3-c3
22	21	20	137	9	0.000	0.66944	3	; C22-C21-C20-H137, prebuilt hc-c3-c3-c3

23	22	21	138	9	0.000	0.66944	3	; C23-C22-C21-H138, prebuilt hc-c3-c3-c3
23	22	21	139	9	0.000	0.66944	3	; C23-C22-C21-H139, prebuilt hc-c3-c3-c3
24	11	10	122	9	0.000	0.65084	3	; C24-C11-C10-H122, prebuilt X-c3-c3-X
24	11	10	123	9	0.000	0.65084	3	; C24-C11-C10-H123, prebuilt X-c3-c3-X
24	11	12	125	9	0.000	0.00000	2	; C24-C11-N12-H125, prebuilt X-c3-n-X
24	25	26	27	9	180.000	3.55640	2	; C24-N25-C26-C27, prebuilt c-n-c3-c
24	25	26	27	9	0.000	3.34720	1	; C24-N25-C26-C27, prebuilt c-n-c3-c
24	25	26	111	9	180.000	2.09200	4	; C24-N25-C26-C111, prebuilt c3-c3-n-c
24	25	26	111	9	180.000	0.62760	3	; C24-N25-C26-C111, prebuilt c3-c3-n-c
24	25	26	111	9	0.000	0.00000	2	; C24-N25-C26-C111, prebuilt c3-c3-n-c
24	25	26	111	9	0.000	2.21752	1	; C24-N25-C26-C111, prebuilt c3-c3-n-c
24	25	26	146	9	0.000	0.00000	2	; C24-N25-C26-H146, prebuilt X-c3-n-X
25	24	11	124	9	180.000	0.00000	2	; N25-C24-C11-H124, prebuilt X-c-c3-X
25	26	27	28	9	180.000	0.00000	2	; N25-C26-C27-O28, prebuilt X-c-c3-X
25	26	27	29	9	180.000	7.11280	1	; N25-C26-C27-N29, prebuilt n-c3-c-n
25	26	27	29	9	180.000	8.36800	2	; N25-C26-C27-N29, prebuilt n-c3-c-n
25	26	111	112	9	0.000	0.65084	3	; N25-C26-C111-C112, prebuilt X-c3-c3-X
25	26	111	213	9	0.000	0.65084	3	; N25-C26-C111-H213, prebuilt X-c3-c3-X
25	26	111	214	9	0.000	0.65084	3	; N25-C26-C111-H214, prebuilt X-c3-c3-X
26	25	24	115	9	180.000	10.46000	2	; C26-N25-C24-O115, prebuilt X-c-n-X
26	27	29	30	9	0.000	0.00000	2	; C26-C27-N29-C30, prebuilt c3-c-n-c3

26	27	29	30	9	180.000	6.27600	1	; C26-C27-N29-C30, prebuilt c3-c-n-c3
26	27	29	147	9	180.000	10.46000	2	; C26-C27-N29-H147, prebuilt X-c-n-X
26	111	112	113	9	0.000	0.41840	4	; C26-C111-C112-N113, prebuilt c3-c3-c-n
26	111	112	113	9	0.000	0.29288	2	; C26-C111-C112-N113, prebuilt c3-c3-c-n
26	111	112	114	9	180.000	0.00000	2	; C26-C111-C112-O114, prebuilt X-c-c3-X
27	26	25	145	9	0.000	0.00000	2	; C27-C26-N25-H145, prebuilt X-c3-n-X
27	26	111	112	9	0.000	0.65084	3	; C27-C26-C111-C112, prebuilt X-c3-c3-X
27	26	111	213	9	0.000	0.65084	3	; C27-C26-C111-H213, prebuilt X-c3-c3-X
27	26	111	214	9	0.000	0.65084	3	; C27-C26-C111-H214, prebuilt X-c3-c3-X
27	29	30	31	9	180.000	3.55640	2	; C27-N29-C30-C31, prebuilt c-n-c3-c
27	29	30	31	9	0.000	3.34720	1	; C27-N29-C30-C31, prebuilt c-n-c3-c
27	29	30	107	9	180.000	2.09200	4	; C27-N29-C30-C107, prebuilt c3-c3-n-c
27	29	30	107	9	180.000	0.62760	3	; C27-N29-C30-C107, prebuilt c3-c3-n-c
27	29	30	107	9	0.000	0.00000	2	; C27-N29-C30-C107, prebuilt c3-c3-n-c
27	29	30	107	9	0.000	2.21752	1	; C27-N29-C30-C107, prebuilt c3-c3-n-c
27	29	30	148	9	0.000	0.00000	2	; C27-N29-C30-H148, prebuilt X-c3-n-X
28	27	26	111	9	180.000	0.00000	2	; O28-C27-C26-C111, prebuilt X-c-c3-X
28	27	26	146	9	0.000	3.34720	1	; O28-C27-C26-H146, prebuilt h1-c3-c-o
28	27	26	146	9	0.000	0.00000	2	; O28-C27-C26-H146, prebuilt h1-c3-c-o
28	27	26	146	9	180.000	0.33472	3	; O28-C27-C26-H146, prebuilt h1-c3-c-o
28	27	29	30	9	180.000	10.46000	2	; O28-C27-N29-C30, prebuilt X-c-n-X

28	27	29	147	9	180.000	10.46000	2	; O28-C27-N29-H147, prebuilt hn-n-c-o
28	27	29	147	9	0.000	8.36800	1	; O28-C27-N29-H147, prebuilt hn-n-c-o
29	27	26	111	9	0.000	0.41840	4	; N29-C27-C26-C111, prebuilt c3-c3-c-n
29	27	26	111	9	0.000	0.29288	2	; N29-C27-C26-C111, prebuilt c3-c3-c-n
29	27	26	146	9	180.000	0.00000	2	; N29-C27-C26-H146, prebuilt X-c-c3-X
29	30	31	32	9	180.000	7.11280	1	; N29-C30-C31-N32, prebuilt n-c3-c-n
29	30	31	32	9	180.000	8.36800	2	; N29-C30-C31-N32, prebuilt n-c3-c-n
29	30	31	106	9	180.000	0.00000	2	; N29-C30-C31-O106, prebuilt X-c-c3-X
29	30	107	108	9	0.000	0.65084	3	; N29-C30-C107-C108, prebuilt X-c3-c3-X
29	30	107	210	9	0.000	0.65084	3	; N29-C30-C107-H210, prebuilt X-c3-c3-X
29	30	107	211	9	0.000	0.65084	3	; N29-C30-C107-H211, prebuilt X-c3-c3-X
30	31	32	33	9	0.000	0.00000	2	; C30-C31-N32-C33, prebuilt c3-c-n-c3
30	31	32	33	9	180.000	6.27600	1	; C30-C31-N32-C33, prebuilt c3-c-n-c3
30	31	32	149	9	180.000	10.46000	2	; C30-C31-N32-H149, prebuilt X-c-n-X
30	107	108	109	9	180.000	0.00000	2	; C30-C107-C108-O109, prebuilt X-c-c3-X
30	107	108	110	9	180.000	0.00000	2	; C30-C107-C108-O110, prebuilt X-c-c3-X
31	30	29	147	9	0.000	0.00000	2	; C31-C30-N29-H147, prebuilt X-c3-n-X
31	30	107	108	9	0.000	0.65084	3	; C31-C30-C107-C108, prebuilt X-c3-c3-X
31	30	107	210	9	0.000	0.65084	3	; C31-C30-C107-H210, prebuilt X-c3-c3-X
31	30	107	211	9	0.000	0.65084	3	; C31-C30-C107-H211, prebuilt X-c3-c3-X
31	32	33	34	9	180.000	3.55640	2	; C31-N32-C33-C34, prebuilt c-n-c3-c

31	32	33	34	9	0.000	3.34720	1	; C31-N32-C33-C34, prebuilt c-n-c3-c
31	32	33	65	9	180.000	2.09200	4	; C31-N32-C33-C65, prebuilt c3-c3-n-c
31	32	33	65	9	180.000	0.62760	3	; C31-N32-C33-C65, prebuilt c3-c3-n-c
31	32	33	65	9	0.000	0.00000	2	; C31-N32-C33-C65, prebuilt c3-c3-n-c
31	32	33	65	9	0.000	2.21752	1	; C31-N32-C33-C65, prebuilt c3-c3-n-c
31	32	33	150	9	0.000	0.00000	2	; C31-N32-C33-H150, prebuilt X-c3-n-X
32	31	30	107	9	0.000	0.41840	4	; N32-C31-C30-C107, prebuilt c3-c3-c-n
32	31	30	107	9	0.000	0.29288	2	; N32-C31-C30-C107, prebuilt c3-c3-c-n
32	31	30	148	9	180.000	0.00000	2	; N32-C31-C30-H148, prebuilt X-c-c3-X
32	33	34	35	9	180.000	0.00000	2	; N32-C33-C34-O35, prebuilt X-c-c3-X
32	33	34	36	9	180.000	7.11280	1	; N32-C33-C34-N36, prebuilt n-c3-c-n
32	33	34	36	9	180.000	8.36800	2	; N32-C33-C34-N36, prebuilt n-c3-c-n
32	33	65	64	9	0.000	0.65084	3	; N32-C33-C65-O64, prebuilt X-c3-c3-X
32	33	65	66	9	0.000	0.65084	3	; N32-C33-C65-C66, prebuilt X-c3-c3-X
32	33	65	171	9	0.000	0.65084	3	; N32-C33-C65-H171, prebuilt X-c3-c3-X
33	32	31	106	9	180.000	10.46000	2	; C33-N32-C31-O106, prebuilt X-c-n-X
33	34	36	37	9	0.000	0.00000	2	; C33-C34-N36-C37, prebuilt c3-c-n-c3
33	34	36	37	9	180.000	6.27600	1	; C33-C34-N36-C37, prebuilt c3-c-n-c3
33	34	36	151	9	180.000	10.46000	2	; C33-C34-N36-H151, prebuilt X-c-n-X
33	65	64	63	9	0.000	1.60247	3	; C33-C65-O64-C63, prebuilt c3-c3-os-c
33	65	64	63	9	180.000	3.34720	1	; C33-C65-O64-C63, prebuilt c3-c3-os-c

33	65	66	172	9	0.000	0.66944	3	; C33-C65-C66-H172, prebuilt hc-c3-c3-c3
33	65	66	173	9	0.000	0.66944	3	; C33-C65-C66-H173, prebuilt hc-c3-c3-c3
33	65	66	174	9	0.000	0.66944	3	; C33-C65-C66-H174, prebuilt hc-c3-c3-c3
34	33	32	149	9	0.000	0.00000	2	; C34-C33-N32-H149, prebuilt X-c3-n-X
34	33	65	64	9	0.000	0.65084	3	; C34-C33-C65-O64, prebuilt X-c3-c3-X
34	33	65	66	9	0.000	0.65084	3	; C34-C33-C65-C66, prebuilt X-c3-c3-X
34	33	65	171	9	0.000	0.65084	3	; C34-C33-C65-H171, prebuilt X-c3-c3-X
34	36	37	38	9	180.000	3.55640	2	; C34-N36-C37-C38, prebuilt c-n-c3-c
34	36	37	38	9	0.000	3.34720	1	; C34-N36-C37-C38, prebuilt c-n-c3-c
34	36	37	152	9	0.000	0.00000	2	; C34-N36-C37-H152, prebuilt X-c3-n-X
34	36	37	153	9	0.000	0.00000	2	; C34-N36-C37-H153, prebuilt X-c3-n-X
35	34	33	65	9	180.000	0.00000	2	; O35-C34-C33-C65, prebuilt X-c-c3-X
35	34	33	150	9	0.000	3.34720	1	; O35-C34-C33-H150, prebuilt h1-c3-c-o
35	34	33	150	9	0.000	0.00000	2	; O35-C34-C33-H150, prebuilt h1-c3-c-o
35	34	33	150	9	180.000	0.33472	3	; O35-C34-C33-H150, prebuilt h1-c3-c-o
35	34	36	37	9	180.000	10.46000	2	; O35-C34-N36-C37, prebuilt X-c-n-X
35	34	36	151	9	180.000	10.46000	2	; O35-C34-N36-H151, prebuilt hn-n-c-o
35	34	36	151	9	0.000	8.36800	1	; O35-C34-N36-H151, prebuilt hn-n-c-o
36	34	33	65	9	0.000	0.41840	4	; N36-C34-C33-C65, prebuilt c3-c3-c-n
36	34	33	65	9	0.000	0.29288	2	; N36-C34-C33-C65, prebuilt c3-c3-c-n
36	34	33	150	9	180.000	0.00000	2	; N36-C34-C33-H150, prebuilt X-c-c3-X

36	37	38	39	9	180.000	7.11280	1	; N36-C37-C38-N39, prebuilt n-c3-c-n
36	37	38	39	9	180.000	8.36800	2	; N36-C37-C38-N39, prebuilt n-c3-c-n
36	37	38	105	9	180.000	0.00000	2	; N36-C37-C38-O105, prebuilt X-c-c3-X
37	38	39	40	9	0.000	0.00000	2	; C37-C38-N39-C40, prebuilt c3-c-n-c3
37	38	39	40	9	180.000	6.27600	1	; C37-C38-N39-C40, prebuilt c3-c-n-c3
37	38	39	154	9	180.000	10.46000	2	; C37-C38-N39-H154, prebuilt X-c-n-X
38	37	36	151	9	0.000	0.00000	2	; C38-C37-N36-H151, prebuilt X-c3-n-X
38	39	40	41	9	180.000	3.55640	2	; C38-N39-C40-C41, prebuilt c-n-c3-c
38	39	40	41	9	0.000	3.34720	1	; C38-N39-C40-C41, prebuilt c-n-c3-c
38	39	40	101	9	180.000	2.09200	4	; C38-N39-C40-C101, prebuilt c3-c3-n-c
38	39	40	101	9	180.000	0.62760	3	; C38-N39-C40-C101, prebuilt c3-c3-n-c
38	39	40	101	9	0.000	0.00000	2	; C38-N39-C40-C101, prebuilt c3-c3-n-c
38	39	40	101	9	0.000	2.21752	1	; C38-N39-C40-C101, prebuilt c3-c3-n-c
38	39	40	155	9	0.000	0.00000	2	; C38-N39-C40-H155, prebuilt X-c3-n-X
39	38	37	152	9	180.000	0.00000	2	; N39-C38-C37-H152, prebuilt X-c-c3-X
39	38	37	153	9	180.000	0.00000	2	; N39-C38-C37-H153, prebuilt X-c-c3-X
39	40	41	42	9	180.000	0.00000	2	; N39-C40-C41-O42, prebuilt X-c-c3-X
39	40	41	43	9	180.000	7.11280	1	; N39-C40-C41-N43, prebuilt n-c3-c-n
39	40	41	43	9	180.000	8.36800	2	; N39-C40-C41-N43, prebuilt n-c3-c-n
39	40	101	102	9	0.000	0.65084	3	; N39-C40-C101-C102, prebuilt X-c3-c3-X
39	40	101	202	9	0.000	0.65084	3	; N39-C40-C101-H202, prebuilt X-c3-c3-X

39	40	101	203	9	0.000	0.65084	3	; N39-C40-C101-H203, prebuilt X-c3-c3-X
40	39	38	105	9	180.000	10.46000	2	; C40-N39-C38-O105, prebuilt X-c-n-X
40	41	43	44	9	0.000	0.00000	2	; C40-C41-N43-C44, prebuilt c3-c-n-c3
40	41	43	44	9	180.000	6.27600	1	; C40-C41-N43-C44, prebuilt c3-c-n-c3
40	41	43	156	9	180.000	10.46000	2	; C40-C41-N43-H156, prebuilt X-c-n-X
40	101	102	103	9	0.000	0.75312	3	; C40-C101-C102-C103, prebuilt c3-c3-c3-c3
40	101	102	103	9	180.000	1.04600	2	; C40-C101-C102-C103, prebuilt c3-c3-c3-c3
40	101	102	103	9	180.000	0.83680	1	; C40-C101-C102-C103, prebuilt c3-c3-c3-c3
40	101	102	204	9	0.000	0.66944	3	; C40-C101-C102-H204, prebuilt hc-c3-c3-c3
40	101	102	205	9	0.000	0.66944	3	; C40-C101-C102-H205, prebuilt hc-c3-c3-c3
41	40	39	154	9	0.000	0.00000	2	; C41-C40-N39-H154, prebuilt X-c3-n-X
41	40	101	102	9	0.000	0.65084	3	; C41-C40-C101-C102, prebuilt X-c3-c3-X
41	40	101	202	9	0.000	0.65084	3	; C41-C40-C101-H202, prebuilt X-c3-c3-X
41	40	101	203	9	0.000	0.65084	3	; C41-C40-C101-H203, prebuilt X-c3-c3-X
41	43	44	45	9	180.000	3.55640	2	; C41-N43-C44-C45, prebuilt c-n-c3-c
41	43	44	45	9	0.000	3.34720	1	; C41-N43-C44-C45, prebuilt c-n-c3-c
41	43	44	97	9	180.000	2.09200	4	; C41-N43-C44-C97, prebuilt c3-c3-n-c
41	43	44	97	9	180.000	0.62760	3	; C41-N43-C44-C97, prebuilt c3-c3-n-c
41	43	44	97	9	0.000	0.00000	2	; C41-N43-C44-C97, prebuilt c3-c3-n-c
41	43	44	97	9	0.000	2.21752	1	; C41-N43-C44-C97, prebuilt c3-c3-n-c
41	43	44	157	9	0.000	0.00000	2	; C41-N43-C44-H157, prebuilt X-c3-n-X

42	41	40	101	9	180.000	0.00000	2	; O42-C41-C40-C101, prebuilt X-c-c3-X
42	41	40	155	9	0.000	3.34720	1	; O42-C41-C40-H155, prebuilt h1-c3-c-o
42	41	40	155	9	0.000	0.00000	2	; O42-C41-C40-H155, prebuilt h1-c3-c-o
42	41	40	155	9	180.000	0.33472	3	; O42-C41-C40-H155, prebuilt h1-c3-c-o
42	41	43	44	9	180.000	10.46000	2	; O42-C41-N43-C44, prebuilt X-c-n-X
42	41	43	156	9	180.000	10.46000	2	; O42-C41-N43-H156, prebuilt hn-n-c-o
42	41	43	156	9	0.000	8.36800	1	; O42-C41-N43-H156, prebuilt hn-n-c-o
43	41	40	101	9	0.000	0.41840	4	; N43-C41-C40-C101, prebuilt c3-c3-c-n
43	41	40	101	9	0.000	0.29288	2	; N43-C41-C40-C101, prebuilt c3-c3-c-n
43	41	40	155	9	180.000	0.00000	2	; N43-C41-C40-H155, prebuilt X-c-c3-X
43	44	45	46	9	180.000	7.11280	1	; N43-C44-C45-N46, prebuilt n-c3-c-n
43	44	45	46	9	180.000	8.36800	2	; N43-C44-C45-N46, prebuilt n-c3-c-n
43	44	45	96	9	180.000	0.00000	2	; N43-C44-C45-O96, prebuilt X-c-c3-X
43	44	97	98	9	0.000	0.65084	3	; N43-C44-C97-C98, prebuilt X-c3-c3-X
43	44	97	199	9	0.000	0.65084	3	; N43-C44-C97-H199, prebuilt X-c3-c3-X
43	44	97	200	9	0.000	0.65084	3	; N43-C44-C97-H200, prebuilt X-c3-c3-X
44	45	46	47	9	0.000	0.00000	2	; C44-C45-N46-C47, prebuilt c3-c-n-c3
44	45	46	47	9	180.000	6.27600	1	; C44-C45-N46-C47, prebuilt c3-c-n-c3
44	45	46	158	9	180.000	10.46000	2	; C44-C45-N46-H158, prebuilt X-c-n-X
44	97	98	99	9	180.000	0.00000	2	; C44-C97-C98-O99, prebuilt X-c-c3-X
44	97	98	100	9	180.000	0.00000	2	; C44-C97-C98-O100, prebuilt X-c-c3-X

45	44	43	156	9	0.000	0.00000	2	; C45-C44-N43-H156, prebuilt X-c3-n-X
45	44	97	98	9	0.000	0.65084	3	; C45-C44-C97-C98, prebuilt X-c3-c3-X
45	44	97	199	9	0.000	0.65084	3	; C45-C44-C97-H199, prebuilt X-c3-c3-X
45	44	97	200	9	0.000	0.65084	3	; C45-C44-C97-H200, prebuilt X-c3-c3-X
45	46	47	48	9	180.000	3.55640	2	; C45-N46-C47-C48, prebuilt c-n-c3-c
45	46	47	48	9	0.000	3.34720	1	; C45-N46-C47-C48, prebuilt c-n-c3-c
45	46	47	95	9	180.000	2.09200	4	; C45-N46-C47-C95, prebuilt c3-c3-n-c
45	46	47	95	9	180.000	0.62760	3	; C45-N46-C47-C95, prebuilt c3-c3-n-c
45	46	47	95	9	0.000	0.00000	2	; C45-N46-C47-C95, prebuilt c3-c3-n-c
45	46	47	95	9	0.000	2.21752	1	; C45-N46-C47-C95, prebuilt c3-c3-n-c
45	46	47	159	9	0.000	0.00000	2	; C45-N46-C47-H159, prebuilt X-c3-n-X
46	45	44	97	9	0.000	0.41840	4	; N46-C45-C44-C97, prebuilt c3-c3-c-n
46	45	44	97	9	0.000	0.29288	2	; N46-C45-C44-C97, prebuilt c3-c3-c-n
46	45	44	157	9	180.000	0.00000	2	; N46-C45-C44-H157, prebuilt X-c-c3-X
46	47	48	49	9	180.000	7.11280	1	; N46-C47-C48-N49, prebuilt n-c3-c-n
46	47	48	49	9	180.000	8.36800	2	; N46-C47-C48-N49, prebuilt n-c3-c-n
46	47	48	94	9	180.000	0.00000	2	; N46-C47-C48-O94, prebuilt X-c-c3-X
46	47	95	196	9	0.000	0.65084	3	; N46-C47-C95-H196, prebuilt X-c3-c3-X
46	47	95	197	9	0.000	0.65084	3	; N46-C47-C95-H197, prebuilt X-c3-c3-X
46	47	95	198	9	0.000	0.65084	3	; N46-C47-C95-H198, prebuilt X-c3-c3-X
47	46	45	96	9	180.000	10.46000	2	; C47-N46-C45-O96, prebuilt X-c-n-X

47	48	49	50	9	0.000	0.00000	2	; C47-C48-N49-C50, prebuilt c3-c-n-c3
47	48	49	50	9	180.000	6.27600	1	; C47-C48-N49-C50, prebuilt c3-c-n-c3
47	48	49	160	9	180.000	10.46000	2	; C47-C48-N49-H160, prebuilt X-c-n-X
48	47	46	158	9	0.000	0.00000	2	; C48-C47-N46-H158, prebuilt X-c3-n-X
48	47	95	196	9	0.000	0.65084	3	; C48-C47-C95-H196, prebuilt X-c3-c3-X
48	47	95	197	9	0.000	0.65084	3	; C48-C47-C95-H197, prebuilt X-c3-c3-X
48	47	95	198	9	0.000	0.65084	3	; C48-C47-C95-H198, prebuilt X-c3-c3-X
48	49	50	51	9	180.000	3.55640	2	; C48-N49-C50-C51, prebuilt c-n-c3-c
48	49	50	51	9	0.000	3.34720	1	; C48-N49-C50-C51, prebuilt c-n-c3-c
48	49	50	90	9	180.000	2.09200	4	; C48-N49-C50-C90, prebuilt c3-c3-n-c
48	49	50	90	9	180.000	0.62760	3	; C48-N49-C50-C90, prebuilt c3-c3-n-c
48	49	50	90	9	0.000	0.00000	2	; C48-N49-C50-C90, prebuilt c3-c3-n-c
48	49	50	90	9	0.000	2.21752	1	; C48-N49-C50-C90, prebuilt c3-c3-n-c
48	49	50	161	9	0.000	0.00000	2	; C48-N49-C50-H161, prebuilt X-c3-n-X
49	48	47	95	9	0.000	0.41840	4	; N49-C48-C47-C95, prebuilt c3-c3-c-n
49	48	47	95	9	0.000	0.29288	2	; N49-C48-C47-C95, prebuilt c3-c3-c-n
49	48	47	159	9	180.000	0.00000	2	; N49-C48-C47-H159, prebuilt X-c-c3-X
49	50	51	52	9	180.000	7.11280	1	; N49-C50-C51-N52, prebuilt n-c3-c-n
49	50	51	52	9	180.000	8.36800	2	; N49-C50-C51-N52, prebuilt n-c3-c-n
49	50	51	89	9	180.000	0.00000	2	; N49-C50-C51-O89, prebuilt X-c-c3-X
49	50	90	91	9	0.000	0.65084	3	; N49-C50-C90-C91, prebuilt X-c3-c3-X

49	50	90	193	9	0.000	0.65084	3	; N49-C50-C90-H193, prebuilt X-c3-c3-X
49	50	90	194	9	0.000	0.65084	3	; N49-C50-C90-H194, prebuilt X-c3-c3-X
50	49	48	94	9	180.000	10.46000	2	; C50-N49-C48-O94, prebuilt X-c-n-X
50	51	52	53	9	0.000	0.00000	2	; C50-C51-N52-C53, prebuilt c3-c-n-c3
50	51	52	53	9	180.000	6.27600	1	; C50-C51-N52-C53, prebuilt c3-c-n-c3
50	51	52	162	9	180.000	10.46000	2	; C50-C51-N52-H162, prebuilt X-c-n-X
50	90	91	92	9	180.000	0.00000	2	; C50-C90-C91-O92, prebuilt X-c-c3-X
50	90	91	93	9	180.000	0.00000	2	; C50-C90-C91-O93, prebuilt X-c-c3-X
51	50	49	160	9	0.000	0.00000	2	; C51-C50-N49-H160, prebuilt X-c3-n-X
51	50	90	91	9	0.000	0.65084	3	; C51-C50-C90-C91, prebuilt X-c3-c3-X
51	50	90	193	9	0.000	0.65084	3	; C51-C50-C90-H193, prebuilt X-c3-c3-X
51	50	90	194	9	0.000	0.65084	3	; C51-C50-C90-H194, prebuilt X-c3-c3-X
51	52	53	54	9	180.000	3.55640	2	; C51-N52-C53-C54, prebuilt c-n-c3-c
51	52	53	54	9	0.000	3.34720	1	; C51-N52-C53-C54, prebuilt c-n-c3-c
51	52	53	163	9	0.000	0.00000	2	; C51-N52-C53-H163, prebuilt X-c3-n-X
51	52	53	164	9	0.000	0.00000	2	; C51-N52-C53-H164, prebuilt X-c3-n-X
52	51	50	90	9	0.000	0.41840	4	; N52-C51-C50-C90, prebuilt c3-c3-c-n
52	51	50	90	9	0.000	0.29288	2	; N52-C51-C50-C90, prebuilt c3-c3-c-n
52	51	50	161	9	180.000	0.00000	2	; N52-C51-C50-H161, prebuilt X-c-c3-X
52	53	54	55	9	180.000	7.11280	1	; N52-C53-C54-N55, prebuilt n-c3-c-n
52	53	54	55	9	180.000	8.36800	2	; N52-C53-C54-N55, prebuilt n-c3-c-n

https://doi.org/10.3390/pharmaceutics18060657

52	53	54	88	9	180.000	0.00000	2	; N52-C53-C54-O88, prebuilt X-c-c3-X
53	52	51	89	9	180.000	10.46000	2	; C53-N52-C51-O89, prebuilt X-c-n-X
53	54	55	56	9	0.000	0.00000	2	; C53-C54-N55-C56, prebuilt c3-c-n-c3
53	54	55	56	9	180.000	6.27600	1	; C53-C54-N55-C56, prebuilt c3-c-n-c3
53	54	55	165	9	180.000	10.46000	2	; C53-C54-N55-H165, prebuilt X-c-n-X
54	53	52	162	9	0.000	0.00000	2	; C54-C53-N52-H162, prebuilt X-c3-n-X
54	55	56	57	9	180.000	3.55640	2	; C54-N55-C56-C57, prebuilt c-n-c3-c
54	55	56	57	9	0.000	3.34720	1	; C54-N55-C56-C57, prebuilt c-n-c3-c
54	55	56	86	9	180.000	2.09200	4	; C54-N55-C56-C86, prebuilt c3-c3-n-c
54	55	56	86	9	180.000	0.62760	3	; C54-N55-C56-C86, prebuilt c3-c3-n-c
54	55	56	86	9	0.000	0.00000	2	; C54-N55-C56-C86, prebuilt c3-c3-n-c
54	55	56	86	9	0.000	2.21752	1	; C54-N55-C56-C86, prebuilt c3-c3-n-c
54	55	56	166	9	0.000	0.00000	2	; C54-N55-C56-H166, prebuilt X-c3-n-X
55	54	53	163	9	180.000	0.00000	2	; N55-C54-C53-H163, prebuilt X-c-c3-X
55	54	53	164	9	180.000	0.00000	2	; N55-C54-C53-H164, prebuilt X-c-c3-X
55	56	57	58	9	180.000	7.11280	1	; N55-C56-C57-N58, prebuilt n-c3-c-n
55	56	57	58	9	180.000	8.36800	2	; N55-C56-C57-N58, prebuilt n-c3-c-n
55	56	57	85	9	180.000	0.00000	2	; N55-C56-C57-O85, prebuilt X-c-c3-X
55	56	86	87	9	0.000	0.65084	3	; N55-C56-C86-O87, prebuilt X-c3-c3-X
55	56	86	190	9	0.000	0.65084	3	; N55-C56-C86-H190, prebuilt X-c3-c3-X
55	56	86	191	9	0.000	0.65084	3	; N55-C56-C86-H191, prebuilt X-c3-c3-X

56	55	54	88	9	180.000	10.46000	2	; C56-N55-C54-O88, prebuilt X-c-n-X
56	57	58	59	9	0.000	0.00000	2	; C56-C57-N58-C59, prebuilt c3-c-n-c3
56	57	58	59	9	180.000	6.27600	1	; C56-C57-N58-C59, prebuilt c3-c-n-c3
56	57	58	167	9	180.000	10.46000	2	; C56-C57-N58-H167, prebuilt X-c-n-X
56	86	87	192	9	0.000	0.66944	3	; C56-C86-O87-H192, prebuilt ho-oh-c3-c3
56	86	87	192	9	0.000	1.04600	1	; C56-C86-O87-H192, prebuilt ho-oh-c3-c3
57	56	55	165	9	0.000	0.00000	2	; C57-C56-N55-H165, prebuilt X-c3-n-X
57	56	86	87	9	0.000	0.65084	3	; C57-C56-C86-O87, prebuilt X-c3-c3-X
57	56	86	190	9	0.000	0.65084	3	; C57-C56-C86-H190, prebuilt X-c3-c3-X
57	56	86	191	9	0.000	0.65084	3	; C57-C56-C86-H191, prebuilt X-c3-c3-X
57	58	59	60	9	180.000	3.55640	2	; C57-N58-C59-C60, prebuilt c-n-c3-c
57	58	59	60	9	0.000	3.34720	1	; C57-N58-C59-C60, prebuilt c-n-c3-c
57	58	59	79	9	180.000	2.09200	4	; C57-N58-C59-C79, prebuilt c3-c3-n-c
57	58	59	79	9	180.000	0.62760	3	; C57-N58-C59-C79, prebuilt c3-c3-n-c
57	58	59	79	9	0.000	0.00000	2	; C57-N58-C59-C79, prebuilt c3-c3-n-c
57	58	59	79	9	0.000	2.21752	1	; C57-N58-C59-C79, prebuilt c3-c3-n-c
57	58	59	168	9	0.000	0.00000	2	; C57-N58-C59-H168, prebuilt X-c3-n-X
58	57	56	86	9	0.000	0.41840	4	; N58-C57-C56-C86, prebuilt c3-c3-c-n
58	57	56	86	9	0.000	0.29288	2	; N58-C57-C56-C86, prebuilt c3-c3-c-n
58	57	56	166	9	180.000	0.00000	2	; N58-C57-C56-H166, prebuilt X-c-c3-X
58	59	60	61	9	180.000	7.11280	1	; N58-C59-C60-N61, prebuilt n-c3-c-n

https://doi.org/10.3390/pharmaceutics18060657

58	59	60	61	9	180.000	8.36800	2	; N58-C59-C60-N61, prebuilt n-c3-c-n
58	59	60	78	9	180.000	0.00000	2	; N58-C59-C60-O78, prebuilt X-c-c3-X
58	59	79	80	9	0.000	0.65084	3	; N58-C59-C79-C80, prebuilt X-c3-c3-X
58	59	79	81	9	0.000	0.65084	3	; N58-C59-C79-C81, prebuilt X-c3-c3-X
58	59	79	183	9	0.000	0.65084	3	; N58-C59-C79-H183, prebuilt X-c3-c3-X
59	58	57	85	9	180.000	10.46000	2	; C59-N58-C57-O85, prebuilt X-c-n-X
59	60	61	62	9	0.000	0.00000	2	; C59-C60-N61-C62, prebuilt c3-c-n-c3
59	60	61	62	9	180.000	6.27600	1	; C59-C60-N61-C62, prebuilt c3-c-n-c3
59	60	61	169	9	180.000	10.46000	2	; C59-C60-N61-H169, prebuilt X-c-n-X
59	79	80	184	9	0.000	0.66944	3	; C59-C79-C80-H184, prebuilt hc-c3-c3-c3
59	79	80	185	9	0.000	0.66944	3	; C59-C79-C80-H185, prebuilt hc-c3-c3-c3
59	79	80	186	9	0.000	0.66944	3	; C59-C79-C80-H186, prebuilt hc-c3-c3-c3
59	79	81	82	9	0.000	0.65084	3	; C59-C79-C81-C82, prebuilt X-c3-c3-X
59	79	81	187	9	0.000	0.66944	3	; C59-C79-C81-H187, prebuilt hc-c3-c3-c3
59	79	81	188	9	0.000	0.66944	3	; C59-C79-C81-H188, prebuilt hc-c3-c3-c3
60	59	58	167	9	0.000	0.00000	2	; C60-C59-N58-H167, prebuilt X-c3-n-X
60	59	79	80	9	0.000	0.65084	3	; C60-C59-C79-C80, prebuilt X-c3-c3-X
60	59	79	81	9	0.000	0.65084	3	; C60-C59-C79-C81, prebuilt X-c3-c3-X
60	59	79	183	9	0.000	0.65084	3	; C60-C59-C79-H183, prebuilt X-c3-c3-X
60	61	62	63	9	180.000	3.55640	2	; C60-N61-C62-C63, prebuilt c-n-c3-c
60	61	62	63	9	0.000	3.34720	1	; C60-N61-C62-C63, prebuilt c-n-c3-c

60	61	62	68	9	180.000	2.09200	4	; C60-N61-C62-C68, prebuilt c3-c3-n-c
60	61	62	68	9	180.000	0.62760	3	; C60-N61-C62-C68, prebuilt c3-c3-n-c
60	61	62	68	9	0.000	0.00000	2	; C60-N61-C62-C68, prebuilt c3-c3-n-c
60	61	62	68	9	0.000	2.21752	1	; C60-N61-C62-C68, prebuilt c3-c3-n-c
60	61	62	170	9	0.000	0.00000	2	; C60-N61-C62-H170, prebuilt X-c3-n-X
61	60	59	79	9	0.000	0.41840	4	; N61-C60-C59-C79, prebuilt c3-c3-c-n
61	60	59	79	9	0.000	0.29288	2	; N61-C60-C59-C79, prebuilt c3-c3-c-n
61	60	59	168	9	180.000	0.00000	2	; N61-C60-C59-H168, prebuilt X-c-c3-X
61	62	63	64	9	180.000	0.00000	2	; N61-C62-C63-O64, prebuilt X-c-c3-X
61	62	63	67	9	180.000	0.00000	2	; N61-C62-C63-O67, prebuilt X-c-c3-X
61	62	68	69	9	0.000	0.65084	3	; N61-C62-C68-C69, prebuilt X-c3-c3-X
61	62	68	175	9	0.000	0.65084	3	; N61-C62-C68-H175, prebuilt X-c3-c3-X
61	62	68	176	9	0.000	0.65084	3	; N61-C62-C68-H176, prebuilt X-c3-c3-X
62	61	60	78	9	180.000	10.46000	2	; C62-N61-C60-O78, prebuilt X-c-n-X
62	63	64	65	9	180.000	11.29680	2	; C62-C63-O64-C65, prebuilt c3-c-os-c3
62	63	64	65	9	0.000	0.00000	1	; C62-C63-O64-C65, prebuilt c3-c-os-c3
62	63	64	65	9	0.000	4.81160	3	; C62-C63-O64-C65, prebuilt c3-c-os-c3
62	68	69	70	9	180.000	0.00000	2	; C62-C68-C69-C70, prebuilt X-c-c3-X
62	68	69	77	9	180.000	0.00000	2	; C62-C68-C69-O77, prebuilt X-c-c3-X
63	62	61	169	9	0.000	0.00000	2	; C63-C62-N61-H169, prebuilt X-c3-n-X
63	62	68	69	9	0.000	0.65084	3	; C63-C62-C68-C69, prebuilt X-c3-c3-X

63	62	68	175	9	0.000	0.65084	3	; C63-C62-C68-H175, prebuilt X-c3-c3-X
63	62	68	176	9	0.000	0.65084	3	; C63-C62-C68-H176, prebuilt X-c3-c3-X
63	64	65	66	9	0.000	1.60247	3	; C63-O64-C65-C66, prebuilt c3-c3-os-c
63	64	65	66	9	180.000	3.34720	1	; C63-O64-C65-C66, prebuilt c3-c3-os-c
63	64	65	171	9	0.000	1.60387	3	; C63-O64-C65-H171, prebuilt X-c3-os-X
64	63	62	68	9	180.000	0.00000	2	; O64-C63-C62-C68, prebuilt X-c-c3-X
64	63	62	170	9	180.000	0.00000	2	; O64-C63-C62-H170, prebuilt X-c-c3-X
64	65	33	150	9	0.000	0.00000	3	; O64-C65-C33-H150, prebuilt h1-c3-c3-os
64	65	33	150	9	0.000	1.04600	1	; O64-C65-C33-H150, prebuilt h1-c3-c3-os
64	65	66	172	9	0.000	0.00000	3	; O64-C65-C66-H172, prebuilt hc-c3-c3-os
64	65	66	172	9	0.000	1.04600	1	; O64-C65-C66-H172, prebuilt hc-c3-c3-os
64	65	66	173	9	0.000	0.00000	3	; O64-C65-C66-H173, prebuilt hc-c3-c3-os
64	65	66	173	9	0.000	1.04600	1	; O64-C65-C66-H173, prebuilt hc-c3-c3-os
64	65	66	174	9	0.000	0.00000	3	; O64-C65-C66-H174, prebuilt hc-c3-c3-os
64	65	66	174	9	0.000	1.04600	1	; O64-C65-C66-H174, prebuilt hc-c3-c3-os
65	33	32	149	9	0.000	0.00000	2	; C65-C33-N32-H149, prebuilt X-c3-n-X
65	64	63	67	9	180.000	11.29680	2	; C65-O64-C63-O67, prebuilt o-c-os-c3
65	64	63	67	9	180.000	5.85760	1	; C65-O64-C63-O67, prebuilt o-c-os-c3
66	65	33	150	9	0.000	0.65084	3	; C66-C65-C33-H150, prebuilt X-c3-c3-X
67	63	62	68	9	180.000	0.00000	2	; O67-C63-C62-C68, prebuilt X-c-c3-X
67	63	62	170	9	0.000	3.34720	1	; O67-C63-C62-H170, prebuilt h1-c3-c-o

67	63	62	170	9	0.000	0.00000	2	; O67-C63-C62-H170, prebuilt h1-c3-c-o
67	63	62	170	9	180.000	0.33472	3	; O67-C63-C62-H170, prebuilt h1-c3-c-o
68	62	61	169	9	0.000	0.00000	2	; C68-C62-N61-H169, prebuilt X-c3-n-X
68	69	70	71	9	180.000	4.18400	2	; C68-C69-C70-C71, prebuilt X-c-ca-X
68	69	70	75	9	180.000	4.18400	2	; C68-C69-C70-C75, prebuilt X-c-ca-X
69	68	62	170	9	0.000	0.65084	3	; C69-C68-C62-H170, prebuilt X-c3-c3-X
69	70	71	72	9	180.000	15.16700	2	; C69-C70-C71-C72, prebuilt X-ca-ca-X
69	70	71	76	9	180.000	15.16700	2	; C69-C70-C71-N76, prebuilt X-ca-ca-X
69	70	75	74	9	180.000	15.16700	2	; C69-C70-C75-C74, prebuilt X-ca-ca-X
69	70	75	180	9	180.000	15.16700	2	; C69-C70-C75-H180, prebuilt X-ca-ca-X
70	69	68	175	9	180.000	0.00000	2	; C70-C69-C68-H175, prebuilt X-c-c3-X
70	69	68	176	9	180.000	0.00000	2	; C70-C69-C68-H176, prebuilt X-c-c3-X
70	71	72	73	9	180.000	15.16700	2	; C70-C71-C72-C73, prebuilt X-ca-ca-X
70	71	72	177	9	180.000	15.16700	2	; C70-C71-C72-H177, prebuilt X-ca-ca-X
70	71	76	181	9	180.000	4.39320	2	; C70-C71-N76-H181, prebuilt X-ca-nh-X
70	71	76	182	9	180.000	4.39320	2	; C70-C71-N76-H182, prebuilt X-ca-nh-X
70	75	74	73	9	180.000	15.16700	2	; C70-C75-C74-C73, prebuilt X-ca-ca-X
70	75	74	179	9	180.000	15.16700	2	; C70-C75-C74-H179, prebuilt X-ca-ca-X
71	70	69	77	9	180.000	4.18400	2	; C71-C70-C69-O77, prebuilt X-c-ca-X
71	70	75	74	9	180.000	15.16700	2	; C71-C70-C75-C74, prebuilt X-ca-ca-X
71	70	75	180	9	180.000	15.16700	2	; C71-C70-C75-H180, prebuilt X-ca-ca-X

71	72	73	74	9	180.000	15.16700	2	; C71-C72-C73-C74, prebuilt X-ca-ca-X
71	72	73	178	9	180.000	15.16700	2	; C71-C72-C73-H178, prebuilt X-ca-ca-X
72	71	70	75	9	180.000	15.16700	2	; C72-C71-C70-C75, prebuilt X-ca-ca-X
72	71	76	181	9	180.000	4.39320	2	; C72-C71-N76-H181, prebuilt X-ca-nh-X
72	71	76	182	9	180.000	4.39320	2	; C72-C71-N76-H182, prebuilt X-ca-nh-X
72	73	74	75	9	180.000	15.16700	2	; C72-C73-C74-C75, prebuilt X-ca-ca-X
72	73	74	179	9	180.000	15.16700	2	; C72-C73-C74-H179, prebuilt X-ca-ca-X
73	72	71	76	9	180.000	15.16700	2	; C73-C72-C71-N76, prebuilt X-ca-ca-X
73	74	75	180	9	180.000	15.16700	2	; C73-C74-C75-H180, prebuilt X-ca-ca-X
74	73	72	177	9	180.000	15.16700	2	; C74-C73-C72-H177, prebuilt X-ca-ca-X
75	70	69	77	9	180.000	4.18400	2	; C75-C70-C69-O77, prebuilt X-c-ca-X
75	70	71	76	9	180.000	15.16700	2	; C75-C70-C71-N76, prebuilt X-ca-ca-X
75	74	73	178	9	180.000	15.16700	2	; C75-C74-C73-H178, prebuilt X-ca-ca-X
76	71	72	177	9	180.000	15.16700	2	; N76-C71-C72-H177, prebuilt X-ca-ca-X
77	69	68	175	9	0.000	3.34720	1	; O77-C69-C68-H175, prebuilt hc-c3-c-o
77	69	68	175	9	0.000	0.00000	2	; O77-C69-C68-H175, prebuilt hc-c3-c-o
77	69	68	175	9	180.000	0.33472	3	; O77-C69-C68-H175, prebuilt hc-c3-c-o
77	69	68	176	9	0.000	3.34720	1	; O77-C69-C68-H176, prebuilt hc-c3-c-o
77	69	68	176	9	0.000	0.00000	2	; O77-C69-C68-H176, prebuilt hc-c3-c-o
77	69	68	176	9	180.000	0.33472	3	; O77-C69-C68-H176, prebuilt hc-c3-c-o
78	60	59	79	9	180.000	0.00000	2	; O78-C60-C59-C79, prebuilt X-c-c3-X

78	60	59	168	9	0.000	3.34720	1	; O78-C60-C59-H168, prebuilt h1-c3-c-o
78	60	59	168	9	0.000	0.00000	2	; O78-C60-C59-H168, prebuilt h1-c3-c-o
78	60	59	168	9	180.000	0.33472	3	; O78-C60-C59-H168, prebuilt h1-c3-c-o
78	60	61	169	9	180.000	10.46000	2	; O78-C60-N61-H169, prebuilt hn-n-c-o
78	60	61	169	9	0.000	8.36800	1	; O78-C60-N61-H169, prebuilt hn-n-c-o
79	59	58	167	9	0.000	0.00000	2	; C79-C59-N58-H167, prebuilt X-c3-n-X
79	81	82	83	9	180.000	0.00000	2	; C79-C81-C82-O83, prebuilt X-c-c3-X
79	81	82	84	9	180.000	0.00000	2	; C79-C81-C82-O84, prebuilt X-c-c3-X
80	79	59	168	9	0.000	0.65084	3	; C80-C79-C59-H168, prebuilt X-c3-c3-X
80	79	81	82	9	0.000	0.65084	3	; C80-C79-C81-C82, prebuilt X-c3-c3-X
80	79	81	187	9	0.000	0.66944	3	; C80-C79-C81-H187, prebuilt hc-c3-c3-c3
80	79	81	188	9	0.000	0.66944	3	; C80-C79-C81-H188, prebuilt hc-c3-c3-c3
81	79	59	168	9	0.000	0.65084	3	; C81-C79-C59-H168, prebuilt X-c3-c3-X
81	79	80	184	9	0.000	0.66944	3	; C81-C79-C80-H184, prebuilt hc-c3-c3-c3
81	79	80	185	9	0.000	0.66944	3	; C81-C79-C80-H185, prebuilt hc-c3-c3-c3
81	79	80	186	9	0.000	0.66944	3	; C81-C79-C80-H186, prebuilt hc-c3-c3-c3
81	82	83	189	9	180.000	9.62320	2	; C81-C82-O83-H189, prebuilt X-c-oh-X
82	81	79	183	9	0.000	0.65084	3	; C82-C81-C79-H183, prebuilt X-c3-c3-X
83	82	81	187	9	180.000	0.00000	2	; O83-C82-C81-H187, prebuilt X-c-c3-X
83	82	81	188	9	180.000	0.00000	2	; O83-C82-C81-H188, prebuilt X-c-c3-X
84	82	81	187	9	0.000	3.34720	1	; O84-C82-C81-H187, prebuilt hc-c3-c-o

84	82	81	187	9	0.000	0.00000	2	; O84-C82-C81-H187, prebuilt hc-c3-c-o
84	82	81	187	9	180.000	0.33472	3	; O84-C82-C81-H187, prebuilt hc-c3-c-o
84	82	81	188	9	0.000	3.34720	1	; O84-C82-C81-H188, prebuilt hc-c3-c-o
84	82	81	188	9	0.000	0.00000	2	; O84-C82-C81-H188, prebuilt hc-c3-c-o
84	82	81	188	9	180.000	0.33472	3	; O84-C82-C81-H188, prebuilt hc-c3-c-o
84	82	83	189	9	180.000	9.62320	2	; O84-C82-O83-H189, prebuilt ho-oh-c-o
84	82	83	189	9	0.000	7.94960	1	; O84-C82-O83-H189, prebuilt ho-oh-c-o
85	57	56	86	9	180.000	0.00000	2	; O85-C57-C56-C86, prebuilt X-c-c3-X
85	57	56	166	9	0.000	3.34720	1	; O85-C57-C56-H166, prebuilt h1-c3-c-o
85	57	56	166	9	0.000	0.00000	2	; O85-C57-C56-H166, prebuilt h1-c3-c-o
85	57	56	166	9	180.000	0.33472	3	; O85-C57-C56-H166, prebuilt h1-c3-c-o
85	57	58	167	9	180.000	10.46000	2	; O85-C57-N58-H167, prebuilt hn-n-c-o
85	57	58	167	9	0.000	8.36800	1	; O85-C57-N58-H167, prebuilt hn-n-c-o
86	56	55	165	9	0.000	0.00000	2	; C86-C56-N55-H165, prebuilt X-c3-n-X
87	86	56	166	9	0.000	0.00000	3	; O87-C86-C56-H166, prebuilt h1-c3-c3-oh
87	86	56	166	9	0.000	1.04600	1	; O87-C86-C56-H166, prebuilt h1-c3-c3-oh
88	54	53	163	9	0.000	3.34720	1	; O88-C54-C53-H163, prebuilt h1-c3-c-o
88	54	53	163	9	0.000	0.00000	2	; O88-C54-C53-H163, prebuilt h1-c3-c-o
88	54	53	163	9	180.000	0.33472	3	; O88-C54-C53-H163, prebuilt h1-c3-c-o
88	54	53	164	9	0.000	3.34720	1	; O88-C54-C53-H164, prebuilt h1-c3-c-o
88	54	53	164	9	0.000	0.00000	2	; O88-C54-C53-H164, prebuilt h1-c3-c-o

88	54	53	164	9	180.000	0.33472	3	; O88-C54-C53-H164, prebuilt h1-c3-c-o
88	54	55	165	9	180.000	10.46000	2	; O88-C54-N55-H165, prebuilt hn-n-c-o
88	54	55	165	9	0.000	8.36800	1	; O88-C54-N55-H165, prebuilt hn-n-c-o
89	51	50	90	9	180.000	0.00000	2	; O89-C51-C50-C90, prebuilt X-c-c3-X
89	51	50	161	9	0.000	3.34720	1	; O89-C51-C50-H161, prebuilt h1-c3-c-o
89	51	50	161	9	0.000	0.00000	2	; O89-C51-C50-H161, prebuilt h1-c3-c-o
89	51	50	161	9	180.000	0.33472	3	; O89-C51-C50-H161, prebuilt h1-c3-c-o
89	51	52	162	9	180.000	10.46000	2	; O89-C51-N52-H162, prebuilt hn-n-c-o
89	51	52	162	9	0.000	8.36800	1	; O89-C51-N52-H162, prebuilt hn-n-c-o
90	50	49	160	9	0.000	0.00000	2	; C90-C50-N49-H160, prebuilt X-c3-n-X
90	91	93	195	9	180.000	9.62320	2	; C90-C91-O93-H195, prebuilt X-c-oh-X
91	90	50	161	9	0.000	0.65084	3	; C91-C90-C50-H161, prebuilt X-c3-c3-X
92	91	90	193	9	0.000	3.34720	1	; O92-C91-C90-H193, prebuilt hc-c3-c-o
92	91	90	193	9	0.000	0.00000	2	; O92-C91-C90-H193, prebuilt hc-c3-c-o
92	91	90	193	9	180.000	0.33472	3	; O92-C91-C90-H193, prebuilt hc-c3-c-o
92	91	90	194	9	0.000	3.34720	1	; O92-C91-C90-H194, prebuilt hc-c3-c-o
92	91	90	194	9	0.000	0.00000	2	; O92-C91-C90-H194, prebuilt hc-c3-c-o
92	91	90	194	9	180.000	0.33472	3	; O92-C91-C90-H194, prebuilt hc-c3-c-o
92	91	93	195	9	180.000	9.62320	2	; O92-C91-O93-H195, prebuilt ho-oh-c-o
92	91	93	195	9	0.000	7.94960	1	; O92-C91-O93-H195, prebuilt ho-oh-c-o
93	91	90	193	9	180.000	0.00000	2	; O93-C91-C90-H193, prebuilt X-c-c3-X

93	91	90	194	9	180.000	0.00000	2	; O93-C91-C90-H194, prebuilt X-c-c3-X
94	48	47	95	9	180.000	0.00000	2	; O94-C48-C47-C95, prebuilt X-c-c3-X
94	48	47	159	9	0.000	3.34720	1	; O94-C48-C47-H159, prebuilt h1-c3-c-o
94	48	47	159	9	0.000	0.00000	2	; O94-C48-C47-H159, prebuilt h1-c3-c-o
94	48	47	159	9	180.000	0.33472	3	; O94-C48-C47-H159, prebuilt h1-c3-c-o
94	48	49	160	9	180.000	10.46000	2	; O94-C48-N49-H160, prebuilt hn-n-c-o
94	48	49	160	9	0.000	8.36800	1	; O94-C48-N49-H160, prebuilt hn-n-c-o
95	47	46	158	9	0.000	0.00000	2	; C95-C47-N46-H158, prebuilt X-c3-n-X
96	45	44	97	9	180.000	0.00000	2	; O96-C45-C44-C97, prebuilt X-c-c3-X
96	45	44	157	9	0.000	3.34720	1	; O96-C45-C44-H157, prebuilt h1-c3-c-o
96	45	44	157	9	0.000	0.00000	2	; O96-C45-C44-H157, prebuilt h1-c3-c-o
96	45	44	157	9	180.000	0.33472	3	; O96-C45-C44-H157, prebuilt h1-c3-c-o
96	45	46	158	9	180.000	10.46000	2	; O96-C45-N46-H158, prebuilt hn-n-c-o
96	45	46	158	9	0.000	8.36800	1	; O96-C45-N46-H158, prebuilt hn-n-c-o
97	44	43	156	9	0.000	0.00000	2	; C97-C44-N43-H156, prebuilt X-c3-n-X
97	98	99	201	9	180.000	9.62320	2	; C97-C98-O99-H201, prebuilt X-c-oh-X
98	97	44	157	9	0.000	0.65084	3	; C98-C97-C44-H157, prebuilt X-c3-c3-X
99	98	97	199	9	180.000	0.00000	2	; O99-C98-C97-H199, prebuilt X-c-c3-X
99	98	97	200	9	180.000	0.00000	2	; O99-C98-C97-H200, prebuilt X-c-c3-X
100	98	97	199	9	0.000	3.34720	1	; O100-C98-C97-H199, prebuilt hc-c3-c-o
100	98	97	199	9	0.000	0.00000	2	; O100-C98-C97-H199, prebuilt hc-c3-c-o

100	98	97	199	9	180.000	0.33472	3	; O100-C98-C97-H199, prebuilt hc-c3-c-o
100	98	97	200	9	0.000	3.34720	1	; O100-C98-C97-H200, prebuilt hc-c3-c-o
100	98	97	200	9	0.000	0.00000	2	; O100-C98-C97-H200, prebuilt hc-c3-c-o
100	98	97	200	9	180.000	0.33472	3	; O100-C98-C97-H200, prebuilt hc-c3-c-o
100	98	99	201	9	180.000	9.62320	2	; O100-C98-O99-H201, prebuilt ho-oh-c-o
100	98	99	201	9	0.000	7.94960	1	; O100-C98-O99-H201, prebuilt ho-oh-c-o
101	40	39	154	9	0.000	0.00000	2	; C101-C40-N39-H154, prebuilt X-c3-n-X
101	102	103	104	9	0.000	0.65084	3	; C101-C102-C103-N104, prebuilt X-c3-c3-X
101	102	103	206	9	0.000	0.65084	3	; C101-C102-C103-H206, prebuilt X-c3-c3-X
101	102	103	207	9	0.000	0.65084	3	; C101-C102-C103-H207, prebuilt X-c3-c3-X
102	101	40	155	9	0.000	0.65084	3	; C102-C101-C40-H155, prebuilt X-c3-c3-X
102	103	104	208	9	0.000	1.25520	3	; C102-C103-N104-H208, prebuilt X-c3-n3-X
102	103	104	209	9	0.000	1.25520	3	; C102-C103-N104-H209, prebuilt X-c3-n3-X
103	102	101	202	9	0.000	0.66944	3	; C103-C102-C101-H202, prebuilt hc-c3-c3-c3
103	102	101	203	9	0.000	0.66944	3	; C103-C102-C101-H203, prebuilt hc-c3-c3-c3
104	103	102	204	9	0.000	0.65084	3	; N104-C103-C102-H204, prebuilt X-c3-c3-X
104	103	102	205	9	0.000	0.65084	3	; N104-C103-C102-H205, prebuilt X-c3-c3-X
105	38	37	152	9	0.000	3.34720	1	; O105-C38-C37-H152, prebuilt h1-c3-c-o
105	38	37	152	9	0.000	0.00000	2	; O105-C38-C37-H152, prebuilt h1-c3-c-o
105	38	37	152	9	180.000	0.33472	3	; O105-C38-C37-H152, prebuilt h1-c3-c-o
105	38	37	153	9	0.000	3.34720	1	; O105-C38-C37-H153, prebuilt h1-c3-c-o

105	38	37	153	9	0.000	0.00000	2	; O105-C38-C37-H153, prebuilt h1-c3-c-o
105	38	37	153	9	180.000	0.33472	3	; O105-C38-C37-H153, prebuilt h1-c3-c-o
105	38	39	154	9	180.000	10.46000	2	; O105-C38-N39-H154, prebuilt hn-n-c-o
105	38	39	154	9	0.000	8.36800	1	; O105-C38-N39-H154, prebuilt hn-n-c-o
106	31	30	107	9	180.000	0.00000	2	; O106-C31-C30-C107, prebuilt X-c-c3-X
106	31	30	148	9	0.000	3.34720	1	; O106-C31-C30-H148, prebuilt h1-c3-c-o
106	31	30	148	9	0.000	0.00000	2	; O106-C31-C30-H148, prebuilt h1-c3-c-o
106	31	30	148	9	180.000	0.33472	3	; O106-C31-C30-H148, prebuilt h1-c3-c-o
106	31	32	149	9	180.000	10.46000	2	; O106-C31-N32-H149, prebuilt hn-n-c-o
106	31	32	149	9	0.000	8.36800	1	; O106-C31-N32-H149, prebuilt hn-n-c-o
107	30	29	147	9	0.000	0.00000	2	; C107-C30-N29-H147, prebuilt X-c3-n-X
107	108	110	212	9	180.000	9.62320	2	; C107-C108-O110-H212, prebuilt X-c-oh-X
108	107	30	148	9	0.000	0.65084	3	; C108-C107-C30-H148, prebuilt X-c3-c3-X
109	108	107	210	9	0.000	3.34720	1	; O109-C108-C107-H210, prebuilt hc-c3-c-o
109	108	107	210	9	0.000	0.00000	2	; O109-C108-C107-H210, prebuilt hc-c3-c-o
109	108	107	210	9	180.000	0.33472	3	; O109-C108-C107-H210, prebuilt hc-c3-c-o
109	108	107	211	9	0.000	3.34720	1	; O109-C108-C107-H211, prebuilt hc-c3-c-o
109	108	107	211	9	0.000	0.00000	2	; O109-C108-C107-H211, prebuilt hc-c3-c-o
109	108	107	211	9	180.000	0.33472	3	; O109-C108-C107-H211, prebuilt hc-c3-c-o
109	108	110	212	9	180.000	9.62320	2	; O109-C108-O110-H212, prebuilt ho-oh-c-o
109	108	110	212	9	0.000	7.94960	1	; O109-C108-O110-H212, prebuilt ho-oh-c-o

110	108	107	210	9	180.000	0.00000	2	; O110-C108-C107-H210, prebuilt X-c-c3-X
110	108	107	211	9	180.000	0.00000	2	; O110-C108-C107-H211, prebuilt X-c-c3-X
111	26	25	145	9	0.000	0.00000	2	; C111-C26-N25-H145, prebuilt X-c3-n-X
111	112	113	215	9	180.000	10.46000	2	; C111-C112-N113-H215, prebuilt X-c-n-X
111	112	113	216	9	180.000	10.46000	2	; C111-C112-N113-H216, prebuilt X-c-n-X
112	111	26	146	9	0.000	0.65084	3	; C112-C111-C26-H146, prebuilt X-c3-c3-X
113	112	111	213	9	180.000	0.00000	2	; N113-C112-C111-H213, prebuilt X-c-c3-X
113	112	111	214	9	180.000	0.00000	2	; N113-C112-C111-H214, prebuilt X-c-c3-X
114	112	111	213	9	0.000	3.34720	1	; O114-C112-C111-H213, prebuilt hc-c3-c-o
114	112	111	213	9	0.000	0.00000	2	; O114-C112-C111-H213, prebuilt hc-c3-c-o
114	112	111	213	9	180.000	0.33472	3	; O114-C112-C111-H213, prebuilt hc-c3-c-o
114	112	111	214	9	0.000	3.34720	1	; O114-C112-C111-H214, prebuilt hc-c3-c-o
114	112	111	214	9	0.000	0.00000	2	; O114-C112-C111-H214, prebuilt hc-c3-c-o
114	112	111	214	9	180.000	0.33472	3	; O114-C112-C111-H214, prebuilt hc-c3-c-o
114	112	113	215	9	180.000	10.46000	2	; O114-C112-N113-H215, prebuilt hn-n-c-o
114	112	113	215	9	0.000	8.36800	1	; O114-C112-N113-H215, prebuilt hn-n-c-o
114	112	113	216	9	180.000	10.46000	2	; O114-C112-N113-H216, prebuilt hn-n-c-o
114	112	113	216	9	0.000	8.36800	1	; O114-C112-N113-H216, prebuilt hn-n-c-o
115	24	11	124	9	0.000	3.34720	1	; O115-C24-C11-H124, prebuilt h1-c3-c-o
115	24	11	124	9	0.000	0.00000	2	; O115-C24-C11-H124, prebuilt h1-c3-c-o
115	24	11	124	9	180.000	0.33472	3	; O115-C24-C11-H124, prebuilt h1-c3-c-o

115	24	25	145	9	180.000	10.46000	2	; O115-C24-N25-H145, prebuilt hn-n-c-o
115	24	25	145	9	0.000	8.36800	1	; O115-C24-N25-H145, prebuilt hn-n-c-o
116	1	2	117	9	180.000	15.16700	2	; H116-C1-C2-H117, prebuilt X-ca-ca-X
116	1	6	119	9	180.000	15.16700	2	; H116-C1-C6-H119, prebuilt X-ca-ca-X
118	5	6	119	9	180.000	15.16700	2	; H118-C5-C6-H119, prebuilt X-ca-ca-X
120	7	8	121	9	180.000	2.61500	2	; H120-N7-C8-H121, prebuilt X-c2-na-X
122	10	11	124	9	0.000	0.65084	3	; H122-C10-C11-H124, prebuilt X-c3-c3-X
123	10	11	124	9	0.000	0.65084	3	; H123-C10-C11-H124, prebuilt X-c3-c3-X
124	11	12	125	9	0.000	0.00000	2	; H124-C11-N12-H125, prebuilt X-c3-n-X
126	15	16	128	9	0.000	0.62760	3	; H126-C15-C16-H128, prebuilt hc-c3-c3-hc
126	15	16	129	9	0.000	0.62760	3	; H126-C15-C16-H129, prebuilt hc-c3-c3-hc
127	15	16	128	9	0.000	0.62760	3	; H127-C15-C16-H128, prebuilt hc-c3-c3-hc
127	15	16	129	9	0.000	0.62760	3	; H127-C15-C16-H129, prebuilt hc-c3-c3-hc
128	16	17	130	9	0.000	0.62760	3	; H128-C16-C17-H130, prebuilt hc-c3-c3-hc
128	16	17	131	9	0.000	0.62760	3	; H128-C16-C17-H131, prebuilt hc-c3-c3-hc
129	16	17	130	9	0.000	0.62760	3	; H129-C16-C17-H130, prebuilt hc-c3-c3-hc
129	16	17	131	9	0.000	0.62760	3	; H129-C16-C17-H131, prebuilt hc-c3-c3-hc
130	17	18	132	9	0.000	0.62760	3	; H130-C17-C18-H132, prebuilt hc-c3-c3-hc
130	17	18	133	9	0.000	0.62760	3	; H130-C17-C18-H133, prebuilt hc-c3-c3-hc
131	17	18	132	9	0.000	0.62760	3	; H131-C17-C18-H132, prebuilt hc-c3-c3-hc
131	17	18	133	9	0.000	0.62760	3	; H131-C17-C18-H133, prebuilt hc-c3-c3-hc

132	18	19	134	9	0.000	0.62760	3	; H132-C18-C19-H134, prebuilt hc-c3-c3-hc
132	18	19	135	9	0.000	0.62760	3	; H132-C18-C19-H135, prebuilt hc-c3-c3-hc
133	18	19	134	9	0.000	0.62760	3	; H133-C18-C19-H134, prebuilt hc-c3-c3-hc
133	18	19	135	9	0.000	0.62760	3	; H133-C18-C19-H135, prebuilt hc-c3-c3-hc
134	19	20	136	9	0.000	0.62760	3	; H134-C19-C20-H136, prebuilt hc-c3-c3-hc
134	19	20	137	9	0.000	0.62760	3	; H134-C19-C20-H137, prebuilt hc-c3-c3-hc
135	19	20	136	9	0.000	0.62760	3	; H135-C19-C20-H136, prebuilt hc-c3-c3-hc
135	19	20	137	9	0.000	0.62760	3	; H135-C19-C20-H137, prebuilt hc-c3-c3-hc
136	20	21	138	9	0.000	0.62760	3	; H136-C20-C21-H138, prebuilt hc-c3-c3-hc
136	20	21	139	9	0.000	0.62760	3	; H136-C20-C21-H139, prebuilt hc-c3-c3-hc
137	20	21	138	9	0.000	0.62760	3	; H137-C20-C21-H138, prebuilt hc-c3-c3-hc
137	20	21	139	9	0.000	0.62760	3	; H137-C20-C21-H139, prebuilt hc-c3-c3-hc
138	21	22	140	9	0.000	0.62760	3	; H138-C21-C22-H140, prebuilt hc-c3-c3-hc
138	21	22	141	9	0.000	0.62760	3	; H138-C21-C22-H141, prebuilt hc-c3-c3-hc
139	21	22	140	9	0.000	0.62760	3	; H139-C21-C22-H140, prebuilt hc-c3-c3-hc
139	21	22	141	9	0.000	0.62760	3	; H139-C21-C22-H141, prebuilt hc-c3-c3-hc
140	22	23	142	9	0.000	0.62760	3	; H140-C22-C23-H142, prebuilt hc-c3-c3-hc
140	22	23	143	9	0.000	0.62760	3	; H140-C22-C23-H143, prebuilt hc-c3-c3-hc
140	22	23	144	9	0.000	0.62760	3	; H140-C22-C23-H144, prebuilt hc-c3-c3-hc
141	22	23	142	9	0.000	0.62760	3	; H141-C22-C23-H142, prebuilt hc-c3-c3-hc
141	22	23	143	9	0.000	0.62760	3	; H141-C22-C23-H143, prebuilt hc-c3-c3-hc

141	22	23	144	9	0.000	0.62760	3	; H141-C22-C23-H144, prebuilt hc-c3-c3-hc
145	25	26	146	9	0.000	0.00000	2	; H145-N25-C26-H146, prebuilt X-c3-n-X
146	26	111	213	9	0.000	0.65084	3	; H146-C26-C111-H213, prebuilt X-c3-c3-X
146	26	111	214	9	0.000	0.65084	3	; H146-C26-C111-H214, prebuilt X-c3-c3-X
147	29	30	148	9	0.000	0.00000	2	; H147-N29-C30-H148, prebuilt X-c3-n-X
148	30	107	210	9	0.000	0.65084	3	; H148-C30-C107-H210, prebuilt X-c3-c3-X
148	30	107	211	9	0.000	0.65084	3	; H148-C30-C107-H211, prebuilt X-c3-c3-X
149	32	33	150	9	0.000	0.00000	2	; H149-N32-C33-H150, prebuilt X-c3-n-X
150	33	65	171	9	0.000	0.65084	3	; H150-C33-C65-H171, prebuilt X-c3-c3-X
151	36	37	152	9	0.000	0.00000	2	; H151-N36-C37-H152, prebuilt X-c3-n-X
151	36	37	153	9	0.000	0.00000	2	; H151-N36-C37-H153, prebuilt X-c3-n-X
154	39	40	155	9	0.000	0.00000	2	; H154-N39-C40-H155, prebuilt X-c3-n-X
155	40	101	202	9	0.000	0.65084	3	; H155-C40-C101-H202, prebuilt X-c3-c3-X
155	40	101	203	9	0.000	0.65084	3	; H155-C40-C101-H203, prebuilt X-c3-c3-X
156	43	44	157	9	0.000	0.00000	2	; H156-N43-C44-H157, prebuilt X-c3-n-X
157	44	97	199	9	0.000	0.65084	3	; H157-C44-C97-H199, prebuilt X-c3-c3-X
157	44	97	200	9	0.000	0.65084	3	; H157-C44-C97-H200, prebuilt X-c3-c3-X
158	46	47	159	9	0.000	0.00000	2	; H158-N46-C47-H159, prebuilt X-c3-n-X
159	47	95	196	9	0.000	0.65084	3	; H159-C47-C95-H196, prebuilt X-c3-c3-X
159	47	95	197	9	0.000	0.65084	3	; H159-C47-C95-H197, prebuilt X-c3-c3-X
159	47	95	198	9	0.000	0.65084	3	; H159-C47-C95-H198, prebuilt X-c3-c3-X

160	49	50	161	9	0.000	0.00000	2	; H160-N49-C50-H161, prebuilt X-c3-n-X
161	50	90	193	9	0.000	0.65084	3	; H161-C50-C90-H193, prebuilt X-c3-c3-X
161	50	90	194	9	0.000	0.65084	3	; H161-C50-C90-H194, prebuilt X-c3-c3-X
162	52	53	163	9	0.000	0.00000	2	; H162-N52-C53-H163, prebuilt X-c3-n-X
162	52	53	164	9	0.000	0.00000	2	; H162-N52-C53-H164, prebuilt X-c3-n-X
165	55	56	166	9	0.000	0.00000	2	; H165-N55-C56-H166, prebuilt X-c3-n-X
166	56	86	190	9	0.000	0.65084	3	; H166-C56-C86-H190, prebuilt X-c3-c3-X
166	56	86	191	9	0.000	0.65084	3	; H166-C56-C86-H191, prebuilt X-c3-c3-X
167	58	59	168	9	0.000	0.00000	2	; H167-N58-C59-H168, prebuilt X-c3-n-X
168	59	79	183	9	0.000	0.65084	3	; H168-C59-C79-H183, prebuilt X-c3-c3-X
169	61	62	170	9	0.000	0.00000	2	; H169-N61-C62-H170, prebuilt X-c3-n-X
170	62	68	175	9	0.000	0.65084	3	; H170-C62-C68-H175, prebuilt X-c3-c3-X
170	62	68	176	9	0.000	0.65084	3	; H170-C62-C68-H176, prebuilt X-c3-c3-X
171	65	66	172	9	0.000	0.65084	3	; H171-C65-C66-H172, prebuilt X-c3-c3-X
171	65	66	173	9	0.000	0.65084	3	; H171-C65-C66-H173, prebuilt X-c3-c3-X
171	65	66	174	9	0.000	0.65084	3	; H171-C65-C66-H174, prebuilt X-c3-c3-X
177	72	73	178	9	180.000	15.16700	2	; H177-C72-C73-H178, prebuilt X-ca-ca-X
178	73	74	179	9	180.000	15.16700	2	; H178-C73-C74-H179, prebuilt X-ca-ca-X
179	74	75	180	9	180.000	15.16700	2	; H179-C74-C75-H180, prebuilt X-ca-ca-X
183	79	80	184	9	0.000	0.62760	3	; H183-C79-C80-H184, prebuilt hc-c3-c3-hc
183	79	80	185	9	0.000	0.62760	3	; H183-C79-C80-H185, prebuilt hc-c3-c3-hc

https://doi.org/10.3390/pharmaceutics18060657

183	79	80	186	9	0.000	0.62760	3	; H183-C79-C80-H186, prebuilt hc-c3-c3-hc
183	79	81	187	9	0.000	0.62760	3	; H183-C79-C81-H187, prebuilt hc-c3-c3-hc
183	79	81	188	9	0.000	0.62760	3	; H183-C79-C81-H188, prebuilt hc-c3-c3-hc
190	86	87	192	9	0.000	0.69733	3	; H190-C86-O87-H192, prebuilt X-c3-oh-X
191	86	87	192	9	0.000	0.69733	3	; H191-C86-O87-H192, prebuilt X-c3-oh-X
202	101	102	204	9	0.000	0.62760	3	; H202-C101-C102-H204, prebuilt hc-c3-c3-hc
202	101	102	205	9	0.000	0.62760	3	; H202-C101-C102-H205, prebuilt hc-c3-c3-hc
203	101	102	204	9	0.000	0.62760	3	; H203-C101-C102-H204, prebuilt hc-c3-c3-hc
203	101	102	205	9	0.000	0.62760	3	; H203-C101-C102-H205, prebuilt hc-c3-c3-hc
204	102	103	206	9	0.000	0.65084	3	; H204-C102-C103-H206, prebuilt X-c3-c3-X
204	102	103	207	9	0.000	0.65084	3	; H204-C102-C103-H207, prebuilt X-c3-c3-X
205	102	103	206	9	0.000	0.65084	3	; H205-C102-C103-H206, prebuilt X-c3-c3-X
205	102	103	207	9	0.000	0.65084	3	; H205-C102-C103-H207, prebuilt X-c3-c3-X
206	103	104	208	9	0.000	1.25520	3	; H206-C103-N104-H208, prebuilt X-c3-n3-X
206	103	104	209	9	0.000	1.25520	3	; H206-C103-N104-H209, prebuilt X-c3-n3-X
207	103	104	208	9	0.000	1.25520	3	; H207-C103-N104-H208, prebuilt X-c3-n3-X
207	103	104	209	9	0.000	1.25520	3	; H207-C103-N104-H209, prebuilt X-c3-n3-X
[pairs] ; Yielded based on rotatable dihedrals								
; atom_i	atom_j	functype						
1	4	1	; C1-C2-C3-C4					
1	9	1	; C1-C2-C3-C9					
1	118	1	; C1-C6-C5-H118					
2	5	1	; C2-C1-C6-C5					
2	119	1	; C2-C1-C6-H119					
2	7	1	; C2-C3-C4-N7					

2	8	1	; C2-C3-C9-C8
2	10	1	; C2-C3-C9-C10
3	6	1	; C3-C2-C1-C6
3	116	1	; C3-C2-C1-H116
3	118	1	; C3-C4-C5-H118
3	120	1	; C3-C4-N7-H120
3	121	1	; C3-C9-C8-H121
3	11	1	; C3-C9-C10-C11
3	122	1	; C3-C9-C10-H122
3	123	1	; C3-C9-C10-H123
4	117	1	; C4-C3-C2-H117
4	10	1	; C4-C3-C9-C10
4	119	1	; C4-C5-C6-H119
4	121	1	; C4-N7-C8-H121
5	9	1	; C5-C4-C3-C9
5	8	1	; C5-C4-N7-C8
5	120	1	; C5-C4-N7-H120
5	116	1	; C5-C6-C1-H116
6	117	1	; C6-C1-C2-H117
6	7	1	; C6-C5-C4-N7
7	118	1	; N7-C4-C5-H118
7	10	1	; N7-C8-C9-C10
8	11	1	; C8-C9-C10-C11
8	122	1	; C8-C9-C10-H122
8	123	1	; C8-C9-C10-H123
9	117	1	; C9-C3-C2-H117
9	120	1	; C9-C8-N7-H120
9	12	1	; C9-C10-C11-N12
9	24	1	; C9-C10-C11-C24
9	124	1	; C9-C10-C11-H124
10	121	1	; C10-C9-C8-H121
10	13	1	; C10-C11-N12-C13
10	125	1	; C10-C11-N12-H125
10	25	1	; C10-C11-C24-N25
10	115	1	; C10-C11-C24-O115
11	14	1	; C11-N12-C13-O14
11	15	1	; C11-N12-C13-C15
11	26	1	; C11-C24-N25-C26
11	145	1	; C11-C24-N25-H145
12	122	1	; N12-C11-C10-H122
12	123	1	; N12-C11-C10-H123
12	25	1	; N12-C11-C24-N25

https://doi.org/10.3390/pharmaceutics18060657

12	115	1	; N12-C11-C24-O115
12	16	1	; N12-C13-C15-C16
12	126	1	; N12-C13-C15-H126
12	127	1	; N12-C13-C15-H127
13	24	1	; C13-N12-C11-C24
13	124	1	; C13-N12-C11-H124
13	17	1	; C13-C15-C16-C17
13	128	1	; C13-C15-C16-H128
13	129	1	; C13-C15-C16-H129
14	125	1	; O14-C13-N12-H125
14	16	1	; O14-C13-C15-C16
14	126	1	; O14-C13-C15-H126
14	127	1	; O14-C13-C15-H127
15	125	1	; C15-C13-N12-H125
15	18	1	; C15-C16-C17-C18
15	130	1	; C15-C16-C17-H130
15	131	1	; C15-C16-C17-H131
16	19	1	; C16-C17-C18-C19
16	132	1	; C16-C17-C18-H132
16	133	1	; C16-C17-C18-H133
17	126	1	; C17-C16-C15-H126
17	127	1	; C17-C16-C15-H127
17	20	1	; C17-C18-C19-C20
17	134	1	; C17-C18-C19-H134
17	135	1	; C17-C18-C19-H135
18	128	1	; C18-C17-C16-H128
18	129	1	; C18-C17-C16-H129
18	21	1	; C18-C19-C20-C21
18	136	1	; C18-C19-C20-H136
18	137	1	; C18-C19-C20-H137
19	130	1	; C19-C18-C17-H130
19	131	1	; C19-C18-C17-H131
19	22	1	; C19-C20-C21-C22
19	138	1	; C19-C20-C21-H138
19	139	1	; C19-C20-C21-H139
20	132	1	; C20-C19-C18-H132
20	133	1	; C20-C19-C18-H133
20	23	1	; C20-C21-C22-C23
20	140	1	; C20-C21-C22-H140
20	141	1	; C20-C21-C22-H141
21	134	1	; C21-C20-C19-H134
21	135	1	; C21-C20-C19-H135

21	142	1	; C21-C22-C23-H142
21	143	1	; C21-C22-C23-H143
21	144	1	; C21-C22-C23-H144
22	136	1	; C22-C21-C20-H136
22	137	1	; C22-C21-C20-H137
23	138	1	; C23-C22-C21-H138
23	139	1	; C23-C22-C21-H139
24	122	1	; C24-C11-C10-H122
24	123	1	; C24-C11-C10-H123
24	125	1	; C24-C11-N12-H125
24	27	1	; C24-N25-C26-C27
24	111	1	; C24-N25-C26-C111
24	146	1	; C24-N25-C26-H146
25	124	1	; N25-C24-C11-H124
25	28	1	; N25-C26-C27-O28
25	29	1	; N25-C26-C27-N29
25	112	1	; N25-C26-C111-C112
25	213	1	; N25-C26-C111-H213
25	214	1	; N25-C26-C111-H214
26	115	1	; C26-N25-C24-O115
26	30	1	; C26-C27-N29-C30
26	147	1	; C26-C27-N29-H147
26	113	1	; C26-C111-C112-N113
26	114	1	; C26-C111-C112-O114
27	145	1	; C27-C26-N25-H145
27	112	1	; C27-C26-C111-C112
27	213	1	; C27-C26-C111-H213
27	214	1	; C27-C26-C111-H214
27	31	1	; C27-N29-C30-C31
27	107	1	; C27-N29-C30-C107
27	148	1	; C27-N29-C30-H148
28	111	1	; O28-C27-C26-C111
28	146	1	; O28-C27-C26-H146
28	30	1	; O28-C27-N29-C30
28	147	1	; O28-C27-N29-H147
29	111	1	; N29-C27-C26-C111
29	146	1	; N29-C27-C26-H146
29	32	1	; N29-C30-C31-N32
29	106	1	; N29-C30-C31-O106
29	108	1	; N29-C30-C107-C108
29	210	1	; N29-C30-C107-H210
29	211	1	; N29-C30-C107-H211

30	33	1	; C30-C31-N32-C33
30	149	1	; C30-C31-N32-H149
30	109	1	; C30-C107-C108-O109
30	110	1	; C30-C107-C108-O110
31	147	1	; C31-C30-N29-H147
31	108	1	; C31-C30-C107-C108
31	210	1	; C31-C30-C107-H210
31	211	1	; C31-C30-C107-H211
31	34	1	; C31-N32-C33-C34
31	65	1	; C31-N32-C33-C65
31	150	1	; C31-N32-C33-H150
32	107	1	; N32-C31-C30-C107
32	148	1	; N32-C31-C30-H148
32	35	1	; N32-C33-C34-O35
32	36	1	; N32-C33-C34-N36
32	64	1	; N32-C33-C65-O64
32	66	1	; N32-C33-C65-C66
32	171	1	; N32-C33-C65-H171
33	106	1	; C33-N32-C31-O106
33	37	1	; C33-C34-N36-C37
33	151	1	; C33-C34-N36-H151
33	63	1	; C33-C65-O64-C63
33	172	1	; C33-C65-C66-H172
33	173	1	; C33-C65-C66-H173
33	174	1	; C33-C65-C66-H174
34	149	1	; C34-C33-N32-H149
34	64	1	; C34-C33-C65-O64
34	66	1	; C34-C33-C65-C66
34	171	1	; C34-C33-C65-H171
34	38	1	; C34-N36-C37-C38
34	152	1	; C34-N36-C37-H152
34	153	1	; C34-N36-C37-H153
35	65	1	; O35-C34-C33-C65
35	150	1	; O35-C34-C33-H150
35	37	1	; O35-C34-N36-C37
35	151	1	; O35-C34-N36-H151
36	65	1	; N36-C34-C33-C65
36	150	1	; N36-C34-C33-H150
36	39	1	; N36-C37-C38-N39
36	105	1	; N36-C37-C38-O105
37	40	1	; C37-C38-N39-C40
37	154	1	; C37-C38-N39-H154

38	151	1	; C38-C37-N36-H151
38	41	1	; C38-N39-C40-C41
38	101	1	; C38-N39-C40-C101
38	155	1	; C38-N39-C40-H155
39	152	1	; N39-C38-C37-H152
39	153	1	; N39-C38-C37-H153
39	42	1	; N39-C40-C41-O42
39	43	1	; N39-C40-C41-N43
39	102	1	; N39-C40-C101-C102
39	202	1	; N39-C40-C101-H202
39	203	1	; N39-C40-C101-H203
40	105	1	; C40-N39-C38-O105
40	44	1	; C40-C41-N43-C44
40	156	1	; C40-C41-N43-H156
40	103	1	; C40-C101-C102-C103
40	204	1	; C40-C101-C102-H204
40	205	1	; C40-C101-C102-H205
41	154	1	; C41-C40-N39-H154
41	102	1	; C41-C40-C101-C102
41	202	1	; C41-C40-C101-H202
41	203	1	; C41-C40-C101-H203
41	45	1	; C41-N43-C44-C45
41	97	1	; C41-N43-C44-C97
41	157	1	; C41-N43-C44-H157
42	101	1	; O42-C41-C40-C101
42	155	1	; O42-C41-C40-H155
42	44	1	; O42-C41-N43-C44
42	156	1	; O42-C41-N43-H156
43	101	1	; N43-C41-C40-C101
43	155	1	; N43-C41-C40-H155
43	46	1	; N43-C44-C45-N46
43	96	1	; N43-C44-C45-O96
43	98	1	; N43-C44-C97-C98
43	199	1	; N43-C44-C97-H199
43	200	1	; N43-C44-C97-H200
44	47	1	; C44-C45-N46-C47
44	158	1	; C44-C45-N46-H158
44	99	1	; C44-C97-C98-O99
44	100	1	; C44-C97-C98-O100
45	156	1	; C45-C44-N43-H156
45	98	1	; C45-C44-C97-C98
45	199	1	; C45-C44-C97-H199

45	200	1	; C45-C44-C97-H200
45	48	1	; C45-N46-C47-C48
45	95	1	; C45-N46-C47-C95
45	159	1	; C45-N46-C47-H159
46	97	1	; N46-C45-C44-C97
46	157	1	; N46-C45-C44-H157
46	49	1	; N46-C47-C48-N49
46	94	1	; N46-C47-C48-O94
46	196	1	; N46-C47-C95-H196
46	197	1	; N46-C47-C95-H197
46	198	1	; N46-C47-C95-H198
47	96	1	; C47-N46-C45-O96
47	50	1	; C47-C48-N49-C50
47	160	1	; C47-C48-N49-H160
48	158	1	; C48-C47-N46-H158
48	196	1	; C48-C47-C95-H196
48	197	1	; C48-C47-C95-H197
48	198	1	; C48-C47-C95-H198
48	51	1	; C48-N49-C50-C51
48	90	1	; C48-N49-C50-C90
48	161	1	; C48-N49-C50-H161
49	95	1	; N49-C48-C47-C95
49	159	1	; N49-C48-C47-H159
49	52	1	; N49-C50-C51-N52
49	89	1	; N49-C50-C51-O89
49	91	1	; N49-C50-C90-C91
49	193	1	; N49-C50-C90-H193
49	194	1	; N49-C50-C90-H194
50	94	1	; C50-N49-C48-O94
50	53	1	; C50-C51-N52-C53
50	162	1	; C50-C51-N52-H162
50	92	1	; C50-C90-C91-O92
50	93	1	; C50-C90-C91-O93
51	160	1	; C51-C50-N49-H160
51	91	1	; C51-C50-C90-C91
51	193	1	; C51-C50-C90-H193
51	194	1	; C51-C50-C90-H194
51	54	1	; C51-N52-C53-C54
51	163	1	; C51-N52-C53-H163
51	164	1	; C51-N52-C53-H164
52	90	1	; N52-C51-C50-C90
52	161	1	; N52-C51-C50-H161

52	55	1	; N52-C53-C54-N55
52	88	1	; N52-C53-C54-O88
53	89	1	; C53-N52-C51-O89
53	56	1	; C53-C54-N55-C56
53	165	1	; C53-C54-N55-H165
54	162	1	; C54-C53-N52-H162
54	57	1	; C54-N55-C56-C57
54	86	1	; C54-N55-C56-C86
54	166	1	; C54-N55-C56-H166
55	163	1	; N55-C54-C53-H163
55	164	1	; N55-C54-C53-H164
55	58	1	; N55-C56-C57-N58
55	85	1	; N55-C56-C57-O85
55	87	1	; N55-C56-C86-O87
55	190	1	; N55-C56-C86-H190
55	191	1	; N55-C56-C86-H191
56	88	1	; C56-N55-C54-O88
56	59	1	; C56-C57-N58-C59
56	167	1	; C56-C57-N58-H167
56	192	1	; C56-C86-O87-H192
57	165	1	; C57-C56-N55-H165
57	87	1	; C57-C56-C86-O87
57	190	1	; C57-C56-C86-H190
57	191	1	; C57-C56-C86-H191
57	60	1	; C57-N58-C59-C60
57	79	1	; C57-N58-C59-C79
57	168	1	; C57-N58-C59-H168
58	86	1	; N58-C57-C56-C86
58	166	1	; N58-C57-C56-H166
58	61	1	; N58-C59-C60-N61
58	78	1	; N58-C59-C60-O78
58	80	1	; N58-C59-C79-C80
58	81	1	; N58-C59-C79-C81
58	183	1	; N58-C59-C79-H183
59	85	1	; C59-N58-C57-O85
59	62	1	; C59-C60-N61-C62
59	169	1	; C59-C60-N61-H169
59	184	1	; C59-C79-C80-H184
59	185	1	; C59-C79-C80-H185
59	186	1	; C59-C79-C80-H186
59	82	1	; C59-C79-C81-C82
59	187	1	; C59-C79-C81-H187

59	188	1	; C59-C79-C81-H188
60	167	1	; C60-C59-N58-H167
60	80	1	; C60-C59-C79-C80
60	81	1	; C60-C59-C79-C81
60	183	1	; C60-C59-C79-H183
60	63	1	; C60-N61-C62-C63
60	68	1	; C60-N61-C62-C68
60	170	1	; C60-N61-C62-H170
61	79	1	; N61-C60-C59-C79
61	168	1	; N61-C60-C59-H168
61	64	1	; N61-C62-C63-O64
61	67	1	; N61-C62-C63-O67
61	69	1	; N61-C62-C68-C69
61	175	1	; N61-C62-C68-H175
61	176	1	; N61-C62-C68-H176
62	78	1	; C62-N61-C60-O78
62	65	1	; C62-C63-O64-C65
62	70	1	; C62-C68-C69-C70
62	77	1	; C62-C68-C69-O77
63	169	1	; C63-C62-N61-H169
63	69	1	; C63-C62-C68-C69
63	175	1	; C63-C62-C68-H175
63	176	1	; C63-C62-C68-H176
63	66	1	; C63-O64-C65-C66
63	171	1	; C63-O64-C65-H171
64	68	1	; O64-C63-C62-C68
64	170	1	; O64-C63-C62-H170
64	150	1	; O64-C65-C33-H150
64	172	1	; O64-C65-C66-H172
64	173	1	; O64-C65-C66-H173
64	174	1	; O64-C65-C66-H174
65	149	1	; C65-C33-N32-H149
65	67	1	; C65-O64-C63-O67
66	150	1	; C66-C65-C33-H150
67	68	1	; O67-C63-C62-C68
67	170	1	; O67-C63-C62-H170
68	169	1	; C68-C62-N61-H169
68	71	1	; C68-C69-C70-C71
68	75	1	; C68-C69-C70-C75
69	170	1	; C69-C68-C62-H170
69	72	1	; C69-C70-C71-C72
69	76	1	; C69-C70-C71-N76

69	74	1	; C69-C70-C75-C74
69	180	1	; C69-C70-C75-H180
70	175	1	; C70-C69-C68-H175
70	176	1	; C70-C69-C68-H176
70	73	1	; C70-C71-C72-C73
70	177	1	; C70-C71-C72-H177
70	181	1	; C70-C71-N76-H181
70	182	1	; C70-C71-N76-H182
70	179	1	; C70-C75-C74-H179
71	77	1	; C71-C70-C69-O77
71	74	1	; C71-C70-C75-C74
71	180	1	; C71-C70-C75-H180
71	178	1	; C71-C72-C73-H178
72	75	1	; C72-C71-C70-C75
72	181	1	; C72-C71-N76-H181
72	182	1	; C72-C71-N76-H182
72	179	1	; C72-C73-C74-H179
73	76	1	; C73-C72-C71-N76
73	180	1	; C73-C74-C75-H180
74	177	1	; C74-C73-C72-H177
75	77	1	; C75-C70-C69-O77
75	76	1	; C75-C70-C71-N76
75	178	1	; C75-C74-C73-H178
76	177	1	; N76-C71-C72-H177
77	175	1	; O77-C69-C68-H175
77	176	1	; O77-C69-C68-H176
78	79	1	; O78-C60-C59-C79
78	168	1	; O78-C60-C59-H168
78	169	1	; O78-C60-N61-H169
79	167	1	; C79-C59-N58-H167
79	83	1	; C79-C81-C82-O83
79	84	1	; C79-C81-C82-O84
80	168	1	; C80-C79-C59-H168
80	82	1	; C80-C79-C81-C82
80	187	1	; C80-C79-C81-H187
80	188	1	; C80-C79-C81-H188
81	168	1	; C81-C79-C59-H168
81	184	1	; C81-C79-C80-H184
81	185	1	; C81-C79-C80-H185
81	186	1	; C81-C79-C80-H186
81	189	1	; C81-C82-O83-H189
82	183	1	; C82-C81-C79-H183

83	187	1	; O83-C82-C81-H187
83	188	1	; O83-C82-C81-H188
84	187	1	; O84-C82-C81-H187
84	188	1	; O84-C82-C81-H188
84	189	1	; O84-C82-O83-H189
85	86	1	; O85-C57-C56-C86
85	166	1	; O85-C57-C56-H166
85	167	1	; O85-C57-N58-H167
86	165	1	; C86-C56-N55-H165
87	166	1	; O87-C86-C56-H166
88	163	1	; O88-C54-C53-H163
88	164	1	; O88-C54-C53-H164
88	165	1	; O88-C54-N55-H165
89	90	1	; O89-C51-C50-C90
89	161	1	; O89-C51-C50-H161
89	162	1	; O89-C51-N52-H162
90	160	1	; C90-C50-N49-H160
90	195	1	; C90-C91-O93-H195
91	161	1	; C91-C90-C50-H161
92	193	1	; O92-C91-C90-H193
92	194	1	; O92-C91-C90-H194
92	195	1	; O92-C91-O93-H195
93	193	1	; O93-C91-C90-H193
93	194	1	; O93-C91-C90-H194
94	95	1	; O94-C48-C47-C95
94	159	1	; O94-C48-C47-H159
94	160	1	; O94-C48-N49-H160
95	158	1	; C95-C47-N46-H158
96	97	1	; O96-C45-C44-C97
96	157	1	; O96-C45-C44-H157
96	158	1	; O96-C45-N46-H158
97	156	1	; C97-C44-N43-H156
97	201	1	; C97-C98-O99-H201
98	157	1	; C98-C97-C44-H157
99	199	1	; O99-C98-C97-H199
99	200	1	; O99-C98-C97-H200
100	199	1	; O100-C98-C97-H199
100	200	1	; O100-C98-C97-H200
100	201	1	; O100-C98-O99-H201
101	154	1	; C101-C40-N39-H154
101	104	1	; C101-C102-C103-N104
101	206	1	; C101-C102-C103-H206

101	207	1	; C101-C102-C103-H207
102	155	1	; C102-C101-C40-H155
102	208	1	; C102-C103-N104-H208
102	209	1	; C102-C103-N104-H209
103	202	1	; C103-C102-C101-H202
103	203	1	; C103-C102-C101-H203
104	204	1	; N104-C103-C102-H204
104	205	1	; N104-C103-C102-H205
105	152	1	; O105-C38-C37-H152
105	153	1	; O105-C38-C37-H153
105	154	1	; O105-C38-N39-H154
106	107	1	; O106-C31-C30-C107
106	148	1	; O106-C31-C30-H148
106	149	1	; O106-C31-N32-H149
107	147	1	; C107-C30-N29-H147
107	212	1	; C107-C108-O110-H212
108	148	1	; C108-C107-C30-H148
109	210	1	; O109-C108-C107-H210
109	211	1	; O109-C108-C107-H211
109	212	1	; O109-C108-O110-H212
110	210	1	; O110-C108-C107-H210
110	211	1	; O110-C108-C107-H211
111	145	1	; C111-C26-N25-H145
111	215	1	; C111-C112-N113-H215
111	216	1	; C111-C112-N113-H216
112	146	1	; C112-C111-C26-H146
113	213	1	; N113-C112-C111-H213
113	214	1	; N113-C112-C111-H214
114	213	1	; O114-C112-C111-H213
114	214	1	; O114-C112-C111-H214
114	215	1	; O114-C112-N113-H215
114	216	1	; O114-C112-N113-H216
115	124	1	; O115-C24-C11-H124
115	145	1	; O115-C24-N25-H145
116	117	1	; H116-C1-C2-H117
116	119	1	; H116-C1-C6-H119
118	119	1	; H118-C5-C6-H119
120	121	1	; H120-N7-C8-H121
122	124	1	; H122-C10-C11-H124
123	124	1	; H123-C10-C11-H124
124	125	1	; H124-C11-N12-H125
126	128	1	; H126-C15-C16-H128

126	129	1	; H126-C15-C16-H129
127	128	1	; H127-C15-C16-H128
127	129	1	; H127-C15-C16-H129
128	130	1	; H128-C16-C17-H130
128	131	1	; H128-C16-C17-H131
129	130	1	; H129-C16-C17-H130
129	131	1	; H129-C16-C17-H131
130	132	1	; H130-C17-C18-H132
130	133	1	; H130-C17-C18-H133
131	132	1	; H131-C17-C18-H132
131	133	1	; H131-C17-C18-H133
132	134	1	; H132-C18-C19-H134
132	135	1	; H132-C18-C19-H135
133	134	1	; H133-C18-C19-H134
133	135	1	; H133-C18-C19-H135
134	136	1	; H134-C19-C20-H136
134	137	1	; H134-C19-C20-H137
135	136	1	; H135-C19-C20-H136
135	137	1	; H135-C19-C20-H137
136	138	1	; H136-C20-C21-H138
136	139	1	; H136-C20-C21-H139
137	138	1	; H137-C20-C21-H138
137	139	1	; H137-C20-C21-H139
138	140	1	; H138-C21-C22-H140
138	141	1	; H138-C21-C22-H141
139	140	1	; H139-C21-C22-H140
139	141	1	; H139-C21-C22-H141
140	142	1	; H140-C22-C23-H142
140	143	1	; H140-C22-C23-H143
140	144	1	; H140-C22-C23-H144
141	142	1	; H141-C22-C23-H142
141	143	1	; H141-C22-C23-H143
141	144	1	; H141-C22-C23-H144
145	146	1	; H145-N25-C26-H146
146	213	1	; H146-C26-C111-H213
146	214	1	; H146-C26-C111-H214
147	148	1	; H147-N29-C30-H148
148	210	1	; H148-C30-C107-H210
148	211	1	; H148-C30-C107-H211
149	150	1	; H149-N32-C33-H150
150	171	1	; H150-C33-C65-H171
151	152	1	; H151-N36-C37-H152

151	153	1	; H151-N36-C37-H153
154	155	1	; H154-N39-C40-H155
155	202	1	; H155-C40-C101-H202
155	203	1	; H155-C40-C101-H203
156	157	1	; H156-N43-C44-H157
157	199	1	; H157-C44-C97-H199
157	200	1	; H157-C44-C97-H200
158	159	1	; H158-N46-C47-H159
159	196	1	; H159-C47-C95-H196
159	197	1	; H159-C47-C95-H197
159	198	1	; H159-C47-C95-H198
160	161	1	; H160-N49-C50-H161
161	193	1	; H161-C50-C90-H193
161	194	1	; H161-C50-C90-H194
162	163	1	; H162-N52-C53-H163
162	164	1	; H162-N52-C53-H164
165	166	1	; H165-N55-C56-H166
166	190	1	; H166-C56-C86-H190
166	191	1	; H166-C56-C86-H191
167	168	1	; H167-N58-C59-H168
168	183	1	; H168-C59-C79-H183
169	170	1	; H169-N61-C62-H170
170	175	1	; H170-C62-C68-H175
170	176	1	; H170-C62-C68-H176
171	172	1	; H171-C65-C66-H172
171	173	1	; H171-C65-C66-H173
171	174	1	; H171-C65-C66-H174
177	178	1	; H177-C72-C73-H178
178	179	1	; H178-C73-C74-H179
179	180	1	; H179-C74-C75-H180
183	184	1	; H183-C79-C80-H184
183	185	1	; H183-C79-C80-H185
183	186	1	; H183-C79-C80-H186
183	187	1	; H183-C79-C81-H187
183	188	1	; H183-C79-C81-H188
190	192	1	; H190-C86-O87-H192
191	192	1	; H191-C86-O87-H192
202	204	1	; H202-C101-C102-H204
202	205	1	; H202-C101-C102-H205
203	204	1	; H203-C101-C102-H204
203	205	1	; H203-C101-C102-H205
204	206	1	; H204-C102-C103-H206

204	207	1	; H204-C102-C103-H207					
205	206	1	; H205-C102-C103-H206					
205	207	1	; H205-C102-C103-H207					
206	208	1	; H206-C103-N104-H208					
206	209	1	; H206-C103-N104-H209					
207	208	1	; H207-C103-N104-H208					
207	209	1	; H207-C103-N104-H209					
[dihedrals] ; impropers								
; atom_i	atom_j	atom_k	atom_l	functype	phase (Deg.)	kd (kJ/mol)	pn	
2	6	1	116	4	180.000	4.60240	2	; C2-C6-C1-H116, prebuilt X-X-ca-ha
1	3	2	117	4	180.000	4.60240	2	; C1-C3-C2-H117, prebuilt X-X-ca-ha
2	4	3	9	4	180.000	4.60240	2	; C2-C4-C3-C9, prebuilt ca-ca-ca-c2
3	5	4	7	4	180.000	4.60240	2	; C3-C5-C4-N7, guess (same as GAFF X -X -ca-ha)
4	6	5	118	4	180.000	4.60240	2	; C4-C6-C5-H118, prebuilt X-X-ca-ha
1	5	6	119	4	180.000	4.60240	2	; C1-C5-C6-H119, prebuilt X-X-ca-ha
4	8	7	120	4	180.000	4.60240	2	; C4-C8-N7-H120, prebuilt X-X-na-hn
7	9	8	121	4	180.000	4.60240	2	; N7-C9-C8-H121, guess (same as GAFF X -X -ca-ha)
3	8	9	10	4	180.000	4.60240	2	; C3-C8-C9-C10, guess (same as GAFF X -X -ca-ha)
11	13	12	125	4	180.000	4.60240	2	; C11-C13-N12-H125, prebuilt c-c3-n-hn
12	14	13	15	4	180.000	43.93200	2	; N12-O14-C13-C15, prebuilt X-X-c-o
11	25	24	115	4	180.000	43.93200	2	; C11-N25-C24-O115, prebuilt X-X-c-o
24	26	25	145	4	180.000	4.60240	2	; C24-C26-N25-H145, prebuilt c-c3-n-hn
26	28	27	29	4	180.000	43.93200	2	; C26-O28-C27-N29, prebuilt X-X-c-o
27	30	29	147	4	180.000	4.60240	2	; C27-C30-N29-H147, prebuilt c-c3-n-hn
30	32	31	106	4	180.000	43.93200	2	; C30-N32-C31-O106, prebuilt X-X-c-o

31	33	32	149	4	180.000	4.60240	2	; C31-C33-N32-H149, prebuilt c-c3-n-hn
33	35	34	36	4	180.000	43.93200	2	; C33-O35-C34-N36, prebuilt X-X-c-o
34	37	36	151	4	180.000	4.60240	2	; C34-C37-N36-H151, prebuilt c-c3-n-hn
37	39	38	105	4	180.000	43.93200	2	; C37-N39-C38-O105, prebuilt X-X-c-o
38	40	39	154	4	180.000	4.60240	2	; C38-C40-N39-H154, prebuilt c-c3-n-hn
40	42	41	43	4	180.000	43.93200	2	; C40-O42-C41-N43, prebuilt X-X-c-o
41	44	43	156	4	180.000	4.60240	2	; C41-C44-N43-H156, prebuilt c-c3-n-hn
44	46	45	96	4	180.000	43.93200	2	; C44-N46-C45-O96, prebuilt X-X-c-o
45	47	46	158	4	180.000	4.60240	2	; C45-C47-N46-H158, prebuilt c-c3-n-hn
47	49	48	94	4	180.000	43.93200	2	; C47-N49-C48-O94, prebuilt X-X-c-o
48	50	49	160	4	180.000	4.60240	2	; C48-C50-N49-H160, prebuilt c-c3-n-hn
50	52	51	89	4	180.000	43.93200	2	; C50-N52-C51-O89, prebuilt X-X-c-o
51	53	52	162	4	180.000	4.60240	2	; C51-C53-N52-H162, prebuilt c-c3-n-hn
53	55	54	88	4	180.000	43.93200	2	; C53-N55-C54-O88, prebuilt X-X-c-o
54	56	55	165	4	180.000	4.60240	2	; C54-C56-N55-H165, prebuilt c-c3-n-hn
56	58	57	85	4	180.000	43.93200	2	; C56-N58-C57-O85, prebuilt X-X-c-o
57	59	58	167	4	180.000	4.60240	2	; C57-C59-N58-H167, prebuilt c-c3-n-hn
59	61	60	78	4	180.000	43.93200	2	; C59-N61-C60-O78, prebuilt X-X-c-o
60	62	61	169	4	180.000	4.60240	2	; C60-C62-N61-H169, prebuilt c-c3-n-hn
62	64	63	67	4	180.000	43.93200	2	; C62-O64-C63-O67, prebuilt X-X-c-o
68	70	69	77	4	180.000	43.93200	2	; C68-C70-C69-O77, prebuilt X-X-c-o

69	71	70	75	4	180.000	4.60240	2	; C69-C71-C70-C75, guess (same as GAFF X -X -ca-ha)
70	72	71	76	4	180.000	4.60240	2	; C70-C72-C71-N76, guess (same as GAFF X -X -ca-ha)
71	73	72	177	4	180.000	4.60240	2	; C71-C73-C72-H177, prebuilt X-X-ca-ha
72	74	73	178	4	180.000	4.60240	2	; C72-C74-C73-H178, prebuilt X-X-ca-ha
73	75	74	179	4	180.000	4.60240	2	; C73-C75-C74-H179, prebuilt X-X-ca-ha
70	74	75	180	4	180.000	4.60240	2	; C70-C74-C75-H180, prebuilt X-X-ca-ha
81	83	82	84	4	180.000	4.60240	2	; C81-O83-C82-O84, prebuilt c3-o-c-oh
90	92	91	93	4	180.000	4.60240	2	; C90-O92-C91-O93, prebuilt c3-o-c-oh
97	99	98	100	4	180.000	4.60240	2	; C97-O99-C98-O100, prebuilt c3-o-c-oh
107	109	108	110	4	180.000	4.60240	2	; C107-O109-C108-O110, prebuilt c3-o-c-oh
111	113	112	114	4	180.000	43.93200	2	; C111-N113-C112-O114, prebuilt X-X-c-o
112	215	113	216	4	180.000	4.60240	2	; C112-H215-N113-H216, prebuilt X-X-n-hn

Table S4. The MDP file for the NVT ensemble.

;define = -DPOSRES ; position restrain the protein
;Run parameters
integrator = md ; leap-frog integrator
nsteps = 2000000 ; 2 * 500000 = 1000 ps=1ns
dt = 0.002 ; 1 fs
; Output control
nstxout = 0 ; save coordinates every 1.0 ps
nstvout = 0 ; save velocities every 1.0 ps
nstenergy = 5000 ; save energies every 1.0 ps
nstlog = 5000 ; update log file every 1.0 ps
; Bond parameters
continuation = no ; first dynamics run
constraint_algorithm = lincs ; holonomic constraints
constraints = all-bonds ; all bonds (even heavy atom-H bonds) constrained
lincs_iter = 1 ; accuracy of LINCS
lincs_order = 4 ; also related to accuracy
; Neighborsearching

cutoff-scheme = Verlet
ns_type = grid ; search neighboring grid cells
nstlist = 10 ; 20 fs, largely irrelevant with Verlet
rcoulomb = 1.0 ; short-range electrostatic cutoff (in nm)
rvdw = 1.0 ; short-range van der Waals cutoff (in nm)
; Electrostatics
coulombtype = PME ; Particle Mesh Ewald for long-range electrostatics
pme_order = 4 ; cubic interpolation
fourierspacing = 0.16 ; grid spacing for FFT
; Temperature coupling is on
tcoupl = V-rescale ; modified Berendsen thermostat
tc-grps = system ; two coupling groups - more accurate
tau_t = 0.1 ; time constant, in ps
ref_t = 298.15 ; reference temperature, one for each group, in K
; Pressure coupling is off
pcoupl = no ; no pressure coupling in NVT
; Periodic boundary conditions
pbc = xyz ; 3-D PBC
; Dispersion correction
DispCorr = EnerPres ; account for cut-off vdW scheme
; Velocity generation
gen_vel = yes ; assign velocities from Maxwell distribution
gen_temp = 298.15 ; temperature for Maxwell distribution
gen_seed = -1 ; generate a random seed

Table S5. The MDP file for the NPT ensemble.

;define =
integrator = md
dt = 0.002 ; ps
nsteps = 50000000 ; 100ns
comm-grps = system
energygrps = system
;
nstxout = 0
nstvout = 0
nstfout = 0
nstlog = 1000
nstenergy = 1000
nstxout-compressed = 1000
compressed-x-grps = system
;
pbc = xyz

cutoff-scheme = Verlet
coulombtype = PME
rcoulomb = 1.0
vdwtype = cut-off
rvdw = 1.0
DispCorr = EnerPres
;
Tcoupl = V-rescale
tau_t = 0.2
tc_grps = system
ref_t = 298.15
;
Pcoupl = Berendsen
pcoupltype = isotropic
tau_p = 2
ref_p = 1.0
compressibility = 4.5e-5
;
gen_vel = no
gen_temp = 298.15
gen_seed = -1
;
freezegrps =
freezedim =
constraints = all-bonds

https://doi.org/10.3390/pharmaceutics18060657
